# Tunable microwave-assisted method for the solvent-free and catalyst-free peracetylation of natural products

**DOI:** 10.3762/bjoc.12.214

**Published:** 2016-10-20

**Authors:** Manuela Oliverio, Paola Costanzo, Monica Nardi, Carla Calandruccio, Raffaele Salerno, Antonio Procopio

**Affiliations:** 1 Department of Health Science, University Magna Graecia of Catanzaro, Viale Europa, Loc. Germaneto, 88100 Catanzaro, Italy; 2 InterRegional Center for Food Safety and Health, University Magna Graecia of Catanzaro, Viale Europa, Loc. Germaneto, 88100 Catanzaro, Italy; 3 Department of Chemistry, Università della Calabria, Cubo 12C, 87036-Arcavacata di Rende (CS), Italy

**Keywords:** catalyst-free, microwaves, peracetylation, polyhydroxylated compounds, solvent-free

## Abstract

**Background:** The peracetylation is a simple chemical modification that can be used to enhance the bioavailability of hydrophilic products and to obtain safe and stable pro-drugs.

**Results:** A totally green, solvent-free and catalyst-free microwave (MW)-assisted method for peracetylation of natural products such as oleuropein, alpha-hederin, quercetin and rutin is presented. By simply tuning the MW heating program, polyols with chemical diverse –OH groups or thermolabile functionalities can be peracetylated to improve the biological activity without degradation of the natural starting molecules. An evaluation of the process greenness was performed.

**Conclusion:** The method is potentially universally applicable for green acetylation of hydrophilic biological molecules, potentially easily scalable for industrial applications, including pharmaceutical, cosmetic and food industry.

## Introduction

Peracetylation of alcohols, phenols and amino groups is a classical protection method in multistep syntheses as well as a transient chemical modification to improve the bioavailaibility and bioactivity of hydrophilic drugs and natural polyols [1–9]. Several in vitro and in vivo studies on peracetylated derivatives of natural products demonstrated that peracetylation increases the cell intake, the intragastric absorbance and the oral bioavailability in respect to the unprotected natural compound [2–3,8–9]. It has been hypothesized that peracetylated molecules can exploit the same pathway than unprotected molecules to pass the cell membrane [5] and, once inside the cells, acetyl groups can be removed by intracellular esterases thus resulting in an augmented dose of active principle [2–3]. Moreover, peracetylation affects the pharmacokinetics by prolonging the half-life of the unprotected molecules whose hydroxy groups are unstable in neutral, slight alkaline or oxidative environments [4]. Furthermore, acetylation is a chemical modification well accepted in a biological environment, being the N-acetylation, N,O-acyl transfer and the deacetylation some of the metabolic processes mediated by cytosolic and mitochondrial acetyl-CoA dependent enzymes, naturally addressed to lower the adverse biological effects or to ameliorate the biological response of several drugs [10]. Besides, well-known commercial drugs, such as acetylsalicylic acid (Aspirin), are acetylated molecules thus demonstrating that peracetylation has been approved by the FDA (Food and Drug Administration). Even the EFSA (European Food Safety Agency) accepted peracetylation as a method to improve the solubility of natural ingredients in fatty matrix (peracetylated starches are labeled as E1420 in the Union list of Food Additives) [11].

Classically peracetylation reactions have been performed by treatment of alcohols and phenols with acid anhydrides or acid chlorides in the presence of bases [12]. Recently, the urgency to find more environmental benign methods for standard transformations, led to the optimization of methods employing preferably acetic anhydride in solvent free conditions, in presence of non-toxic homogeneous catalysts such as environmental safe Lewis acids [13–14]. Moreover, the need to easily recover and reuse the catalyst, thus reducing the work-up procedure to a simple filtration, resulted in the growing use of heterogeneous or supported catalysts [15–17], solid nanopowders or nanoparticles [18–19], non-metal-based heterogeneous acetylation catalysts [20–22], as well as natural marine clays instead of homogeneous catalysts as reaction activators [23]. Even if these methods allow a complete peracetylation of several functionalities at room temperature in good to excellent yields, it is worth noting that some of them use metal-based catalysts needing long preparation procedures, or in the case of non-metal-based catalysts, inorganic acids are employed to activate acylation. On the other hand, the increasing attention to the final product safety, strictly connected to the consumers safety and health, push the pharmaceutical and food companies to prefer methods that allows to minimize the opportunity of the final product to get in touch with chemical additives. At the best of our knowledge, only few reports exist dealing with the acetylation of hydroxy groups under catalyst-free conditions. Most of them use alternative acetylating agents [24] or alternative energy sources [25], but none of them has been applied to complex molecules or natural products. Between them a crucial report about the MW-assisted solvent-free and catalyst-free acetylation of anthranilic acid using acetic anhydride as acetylating agent, kept our attention [26]. According to this report, few minutes at maximum MW power (1000 Watt), without any temperature control, are needed to quantitative acetylate anthranilic acid. Obviously such uncontrolled conditions are not suitable for natural molecules, as they are often characterized by different moieties bonded each other by thermo or acid/base labile ester bonds; nevertheless such report furnished us the proof of principle that the rapid rise of temperature due to MW can catalyse acetylation using acetic anhydride. So, starting from this statement and trading on our experience in catalyst-free reactions [27–28] and MW-assisted chemistry [29–35], we propose here a universal MW-assisted method for peracetylation of multifunctional compounds. The method is totally green and safe as it employs food grade acetic anhydride as acetylating agent, solvent-free and catalyst-free conditions, an easy work-up procedure affording the peracetylated molecules without any chromatographic purification. The possibility to contemporary acetylate several chemically diverse –OH groups on thermolabile molecules simply tuning the heating program on the MW-oven is discussed.

## Results and Discussion

Our work started from the results reported in literature for the MW-assisted acetylation of anthranilic acid. As the water content can be a limiting factor for the acetylation equilibrium, a pre-drying procedure is often required before the use of acetic anhydride [26]. In order to optimize a cheap, safe, green and easily scalable method for industrial application we decided to use food grade acetic anhydride (Eastman) as acetylating agent, after its anhydrification by simple passing it through a bed of activated molecular sieves under nitrogen steam. Such anhydrification technique was already proposed for several organic solvents as safer alternative to classical methods using reactive metals, metal hydrides or solvent distillation [36].

**Figure 1:**
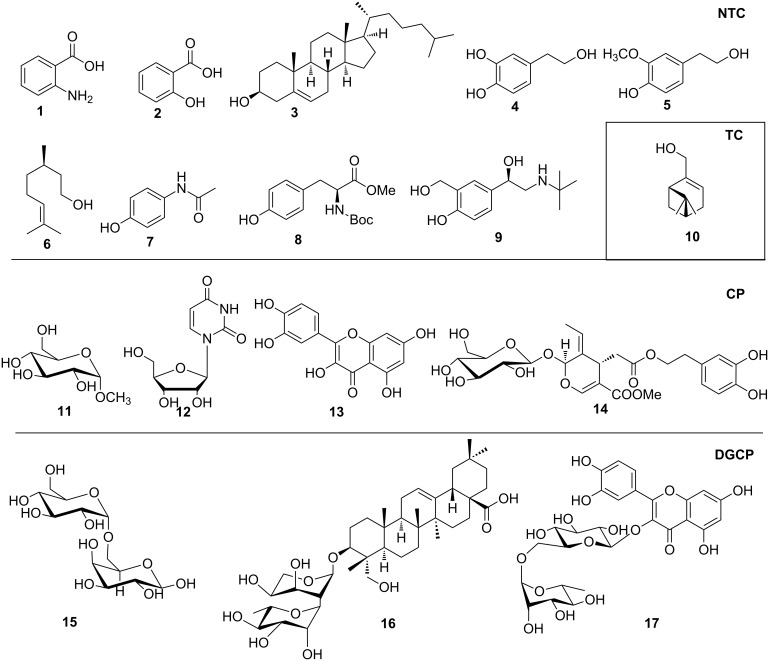
Chemical structures of bioactive substrates and their partition in subsets.

Moreover, in order to explore the versatility of the methodology we selected a set of representative molecules of different categories (Figure 1) such as pharmaceuticals (salicylic acid (**2**), paracetamol (**7**) and salbutamol (**9**)), cosmetic ingredients (cytronellol (**6**) and myrtenol (**10**)), biomolecules (cholesterol (**3**), N-Boc- tyrosine methyl ester (**8**), uridine (**12**) and methyl-α-D-glucopyranoside (**11**)) and natural antioxidant compounds in their simple (hydroxytyrosol (**4**), homovanillic alcohol (**5**), quercetin (**13**)) or glycosylated forms (oleuropein (**14**), rutin (**17**), alpha-hederin (**16**)). Because of their heterogeneity in terms of thermostability, number and reactivity of –OH groups, we decided to split the complete set in four subsets: molecules characterized by a good thermostability with up to three –OH groups (non thermolabile compounds, NTC), molecules characterized by a strong thermolability (thermolabile compounds, TC), complex molecules characterized by at least two different chemical moieties and/or more than 3 –OH groups, (complex polyols, CP) and complex molecules with a disaccharide moiety, namely carrying a huge number of chemically different –OH groups (di-glycosylated complex polyols, DGCP). To set the reaction conditions we used the compounds belonging to the NTC group (**2**–**9**, Figure 1) comparing the obtained results to the literature reported for the anthranilic acid (**1**).

All the molecules were reacted in a Synthos 3000 (Anton-Paar) microwave oven equipped with an external IR sensor for the temperature control; they were solubilized in dry acetic anhydride in a concentration of 0.1 mmol/mL without any other solvent or catalyst, in presence of the 10% w/w of activated molecular sieves to preserve dryness. Moreover, it has been reported that molecular sieves can have a role in acetylation reaction, both under classical or alternative heating mode, thanks to their soft base character and MW absorbing power, respectively [37–40]. The reaction performed without molecular sieves gave rise to worse results (data not shown), even if a catalytic role could not be proved in our case, because the reactions were activated and gave rise to moderate yields as in a typical equilibrium system.

**Scheme 1:**
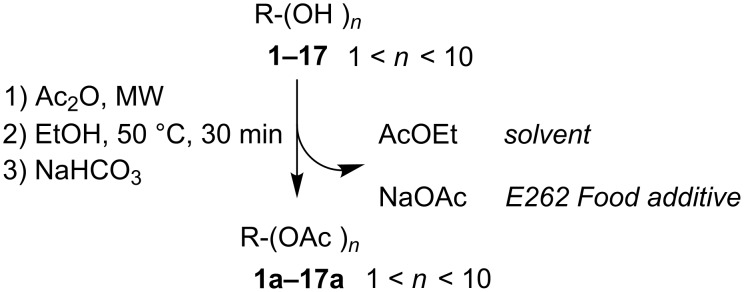
Solvent-free and catalyst-free MW-assisted acetylation protocol.

The reactions were monitored by TLC or GC–MS until the reagent disappeared and the work-up procedure was optimized in order to minimize the wastes of the process. Namely the reaction mixture was reacted with ethanol in order to eliminate the excess of acetic anhydride thus producing acetic acid and AcOEt, a common organic solvent with acceptable safety and environmental characteristics [41], recoverable by simple evaporation under reduced pressure. After evaporation, the acetic acid was neutralized by adding a saturated solution of NaHCO_3_ and the acetylated products were recovered by decantation without any other purification. The water solution of NaOAc obtained as byproduct can be reused as component for buffer solutions or as pickling agent for foods [11]. A complete scheme of the reaction protocol is depicted in Scheme 1.

Concerning the microwave settings we decided to extend the reaction time in respect to the reported acetylation of anthranilic acid [26] and to carefully monitor the microwave power, thus realizing a simple P-controlled program, corresponding to an internal temperature program, to softly reach the maximum power. An adjustment of the NCT P-program settings was performed for the other subsets, characterized by more instable or complex molecules, as it is described in Table 1.

**Table 1:** P-controlled MW programs for peracetylation of compounds listed in Figure 1 (Synthos 3000, equipped with 64-MG5 rotor).

Entry	Method	Time (min)	Power (W)	*T*_internal_ (°C)^a^	*T*_IR_ (°C)

1	NTC	0 → 5 5 → 17 17 → 20	0 → 300 300 0	25 → 100 100 100 → 25	85
2	TC	0 → 2 2 → 7 7 → 12 12 → 22 22 → 25	0 → 130 130 130 → 300 300 300 → 0	0 → 60 60 60 → 100 100 100 → 25	50 50 85 85 85
3	CP	0 → 5 5 → 10 10 → 12 12 → 62 62 → 65	0 → 300 300 300 → 400 400 0	0 → 100 100 100 → 120 120 120 → 25	85 85 105 105 105
4	DGCP	0 → 5 5 → 10 10 → 15 15 → 45 45 → 50 50 → 90 90 → 93	0 → 300 300 300 → 400 400 400 → 500 500 500 → 0	25 → 100 100 100 → 120 120 120 → 145 145 145 → 25	85 85 105 105 120 120 120

^a^ Internal reaction temperature, related to IR limit temperature by the following equation: *T*_internal_ = 1.214 × *T*_IR._ Maximum internal temperature for each category was established between many, by controlling the cleanness of the reaction profile.

**Figure 2:**
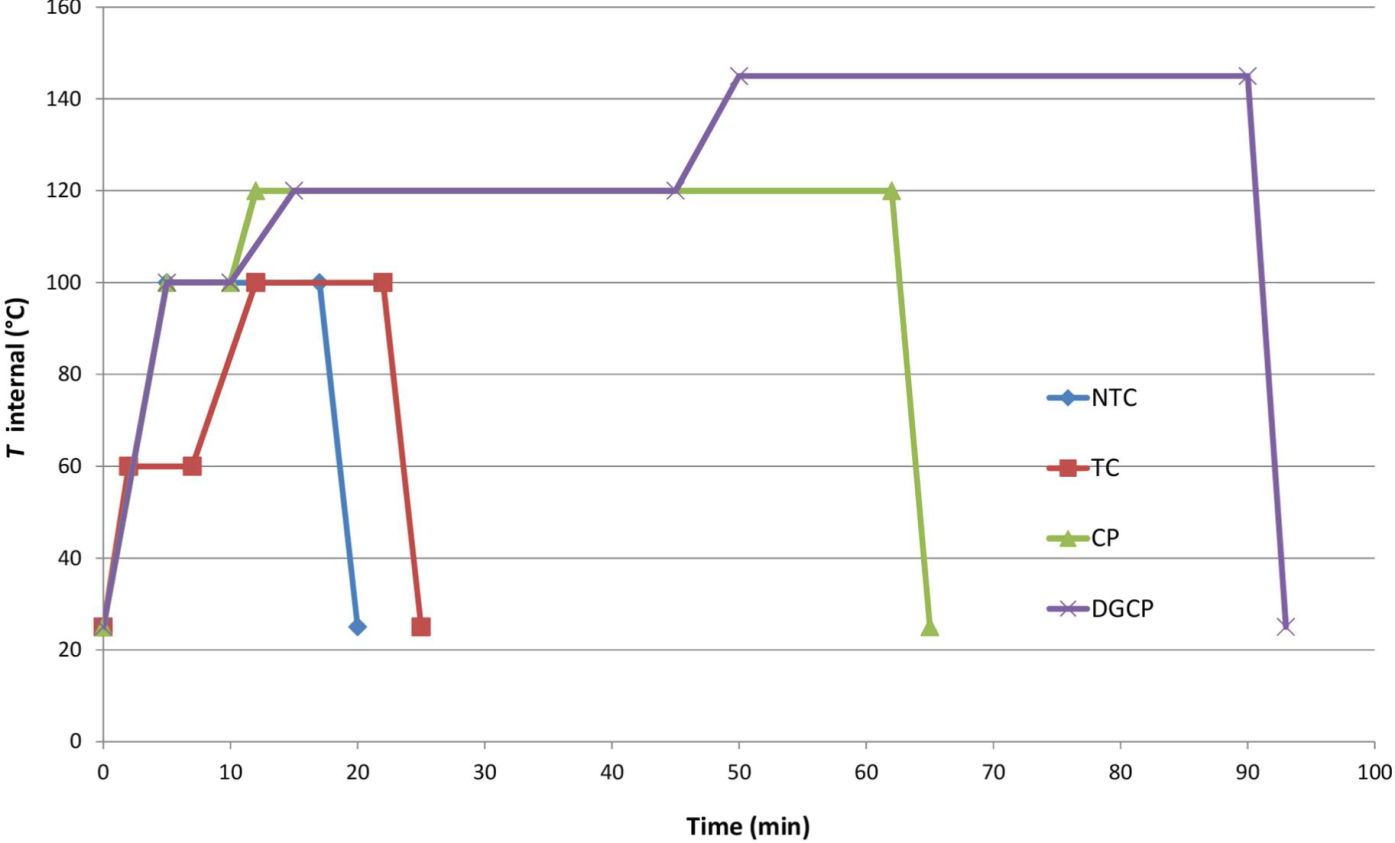
MW-assisted acetylation T-program for different subset of substrates.

The internal temperature profiles corresponding to each P-program are depicted in Figure 2.

The yields of the obtained peracetylated products (Table 2) are referred to isolated compounds, that have been fully characterized by HRMS, ^1^H and ^13^C NMR when unknown. The substrates giving rise to a mixture of inseparable acetylated derivatives were identified by LC/HRMS. In particular, UHPLC combined with an Orbitrap mass spectrometer at high resolving power, enabled the detection and the accurate mass measurement (<5 ppm error) of all the acetylated analytes in the mixture. The yields have been calculated on the peak intensities selecting the analytes having an ion current intensity value 10 fold lower than the main product. A little portion of the mixture was purified by flash chromatography for the structural characterization of the major product.

**Table 2:** Solvent free and catalyst free peracetylation MW assisted of alcohols and polyols.

Entry	Path	Product	Conv. (%)^a^	Yield (%)^b^	N° Run

1	NTC	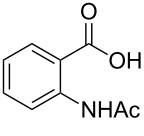 **1a**	100	100	1
2	NTC	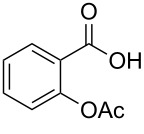 **2a**	100	100	1
3	NTC	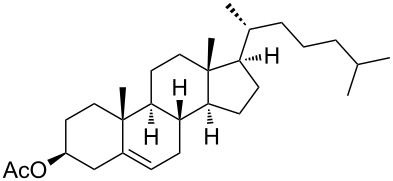 **3a**	70	62	3
4	NTC	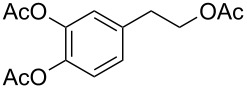 **4a**	100	100	1
5	NTC	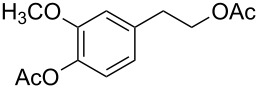 **5a**	100	100	1
6	NTC	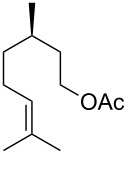 **6a**	100	100^c^	1
7	NTC	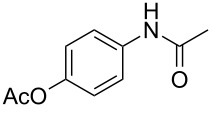 **7a**	95	93	1
8	NTC	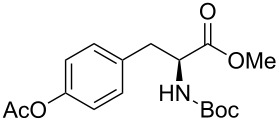 **8a**	100	95	2
9	NTC	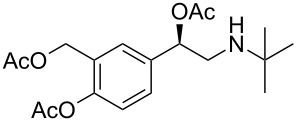 **9a**	100	30^d^	3
10	TC	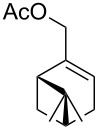 **10a**	100	100	1
11	CP	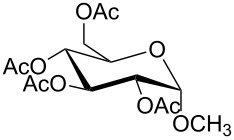 **11a**	100	70^d,e^	2
12	CP	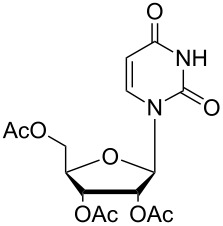 **12a**	100	92^d,e^	2
13	CP	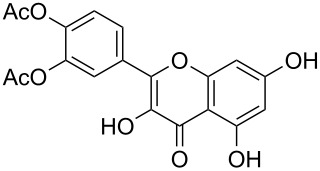 **13a**^f^	94	60^d^	1
14	CP	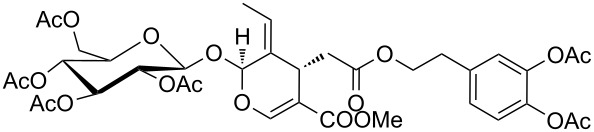 **14a**	100	100	1
15	DGCP	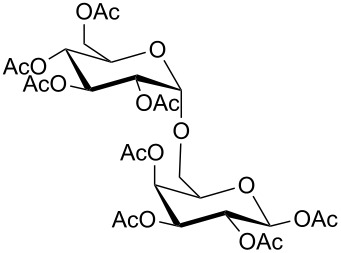 **15a**	100	50^d,e^	2
16	DGCP	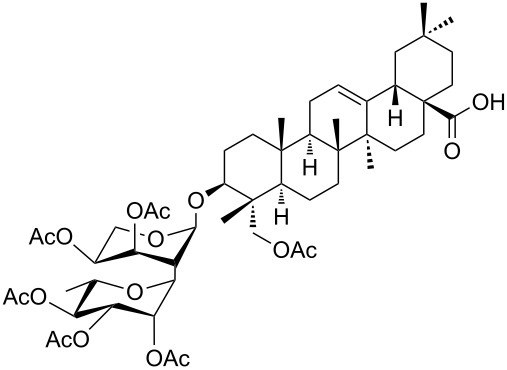 **16a**	100	85^d,e^	2
17	DGCP	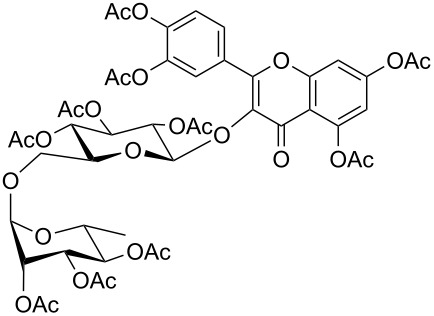 **17a**	100	45^e^	2

^a^Conversion determined by GC–MS or LC–MS and calculated as (100 − % area under the reagent peak). ^b^Isolated products. ^c^Volatile product. ^d^A mix of acetylated forms has been obtained. The yield was determined on the major product after purification ^e^Fresh Ac_2_O added before each cycle. ^f^Major isobar form from LC–MS. No attribution about the position of acetyl groups was made.

As it is reported in Table 2 we obtained good to excellent yields of acetylated products for all the substrates belonging to NTC group. More than one reaction cycle was needed when the reactant had a low solubility in acetic anhydride as in the case of cholesterol, *N*-Boc-tyrosine methyl ester and salbutamol (entries 3, 8 and 9, Table 2). Comparing to data reported in the literature [42–43], the modest yields of peracetylated product obtained in case of cholesterol **3** and salbutamol **9** were balanced by the absence of catalyst and by the reaction speed due to microwaves. The applied P-program was compatible with the *N*-Boc protecting group already present on the tyrosine methyl ester **8**. Concerning salbutamol (**9**) the reported yield was referred to the peracetylated product **9a** even if a quantitative conversion was registered and the obtained mixture of different acetylated derivatives was fully identified by GC–MS (see Supporting Information File 1). In this last case the change of the P-program was ineffective in increasing the yield of **9a** in respect to the other acetylated forms (data not shown).

As it can be argued by Figure 2 and Table 1, that the TC program (entry 2, Table 1) characterized by an intermediate step at lower temperature was necessary to avoid the degradation of TCs when exposed to the NTC program. In our case only a substrate, namely myrtenol (**10**), characterized by an allyl alcohol moiety, needed this softer program to be acetylated without degradation and/or polymerization (entry 10, Table 2).

Molecules bearing more than three –OH groups (CPs), besides *N*- or *O*- glycosidic bonds, needed a program composed of two steps with increasing temperature and power to complete (entry 3, Table 1). During the first step, where the MW conditions are very similar to NTC program, the more reactive –OH groups, such as primary or non-hindered phenolic groups, were acetylated, while a second step at higher power and temperature (Figure 2) was needed to realize the acetylation of all the –OH functionalities. The two-step temperature increase allowed to activate the reaction, limiting the exposure time of such compounds to the program higher temperature, thus preserving sensitive bonds.

**Figure 3:**
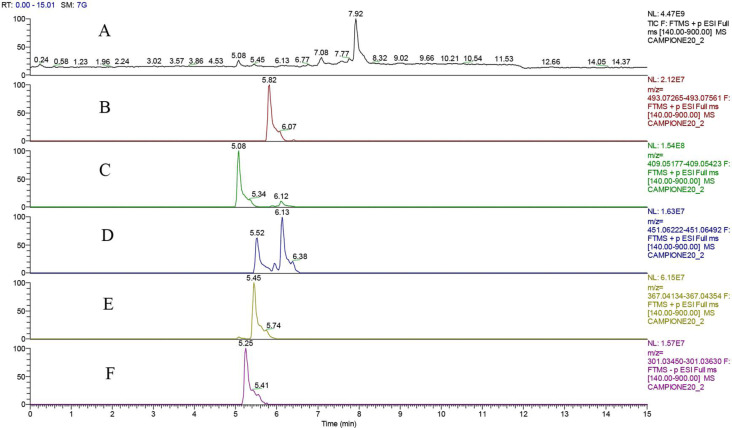
LCHRMS (*m*/*z*, [M + Na]^+^ and [M − H]^−^ only for entry F) spectrum of *O*-acetylated quercetin (reaction mix) in total ion current (TIC, entry A) and extract ion Current (XIC, entries B–F) relative to main acetylated-forms: tetra-*O*-acetylated quercetin (8% of the mixture, entry B), di-*O*-acetylated quercetin (60% of the mixture, entry C), tri-*O*-acetylated quercetin (7% of the mixture, entry D), mono-*O*-acetylated-quercetin (25% of the mixture, entry E). The conversion was estimated around 96%, because of the presence of 6% of unreacted quercetin (entry F).

All the polyols were peracetylated in good yields (entries 11–14, Table 2), even if in some cases more than one reaction cycle was necessary for complete conversion (entries 11, 12, 15, 16 and 17, Table 2). The only exception was the natural product quercetin (**13**) that, despite a quantitative conversion after the first acetylation run, surprisingly gave a complex mixture of differently acetylated forms (Figure 3), among them the di-*O*-acetylated quercetin **13a** was the major product (60% of the total reaction products, entry 13, Table 2).

As the permeability and/or bioavailability of polyols is increased, no matter if the molecules are fully or partially acetylated, we decided to identify the full mixture by LC–MS analysis. We also provided a purification of the major product in order to carry out a structural characterization and to determine the yield of isolated product. Figure 3 shows the LC–HRMS of *O*-acetylated quercetin reaction mixture (for ^1^H and ^13^C NMR spectra of the mixture see Supporting Information File 1). Chemical structures of non-fully acetylated forms, i.e., tetra-*O*-acetylated-quercetin (8% of the mixture, entry B, Figure 3), di-*O*-acetylated quercetin (60% of the mixture, entry C, Figure 3), tri-*O*-acetylated quercetin (7% of the mixture, entry D, Figure 3), mono-*O*-acetylated-quercetin (25% of the mixture, entry E, Figure 3), could not be univocally assigned, except for the major product, characterized by ^1^H NMR. The mixture of the acetylated forms can in principle work as bioactive component when used as crude reaction miture without purification.

Comparable results in terms of conversion to those collected for the CP group were obtained with the last group of molecules (DGCPs, entries 15–17, Table 2). Nevertheless, DGCP needed a MW three steps program, reaching the maximum power of 500 W (entry 4, Table 1), due to the increased number of chemical diverse –OH groups. In all cases a mixture of different acetylated forms were obtained and the prevalence of the peracetylated product was inversely proportional to the number of –OH groups within the molecule. So, good yields of peracetylated product **16a** were obtained from α-hederin (entry 16, Table 2), while medium yields were obtained for peracetylated-β-D-lactose **15a** (entry 15, Table 2) and peracetylated rutin **17a** (entry 17, Table 2) where the remaining 50% of product was constituted by a mixture of different non-fully acetylated forms, even after more than one acetylation run. Moreover, peracetylation of rutin (**17**), which is the di-glycosylated derivative of quercetin (**13**), demonstrated that an additional acetylation run could give rise also to a quercetin peracetylation.

An evaluation of the greenness of the process was performed by calculation of atom economy (AE), reaction mass efficiency (RME), mass intensity (MI) and mass productivity (MP), according to the definition summarized by Constable et al. [44] (see Supporting Information File 1). The values for all the previous parameters, calculated for the 17 performed reactions, are reported in Table 3. AE was referred to the peracetylated compound, as it was the reaction desired product.

**Table 3:** Process green chemistry metrics.

Entry	Yield (%)	AE (%)	RME (%)	MI	MP (%)

1	100	75	75	2	50
2	100	75	75	2	50
3	62	88	54	3	33
4	100	61	61	2	50
5	100	68	68	2	50
6	55	77	42	3	33
7	93	76	71	2	50
8	90	85	76	2	50
9	30	67	20	7	14
10	100	76	76	2	50
11	70	51	36	3	33
12	92	67	62	2	50
13	60	76	46	3	33
14	100	75	75	2	50
15	50	58	29	5	20
16	85	70	59	3	33
17	45	63	28	6	16

As expected, RME is a more realistic parameter than AE due to the influence of the reaction yield. Because MI and MP consider all the masses implied in the process and take into account yield, solvents and reaction auxiliaries, they are more useful parameters to evaluate the sustainability of the process from an industrial point of view [44]. For the calculation of MI and MP the reaction work-up was included as a reaction step (see Supporting Information File 1) because the products obtained by the hydrolysis of the excess of Ac_2_O used, both as reaction reagent and solvent, are useful chemicals. The calculated values were good, thus demonstrating the versatility and sustainability of the process. Our prevision shows how the process remains reasonable in terms of mass productivity, ranging from 16% to 50%.

Finally a scaled protocol extended to the maximum oven capacity was tested using oleuropein (**14**) as test compound. 30 mL vials were reacted in the Synthos 3000 XF-100 rotor, after filling each vial with a ten-fold quantity of reactants, reaching a total processed oleuropein of 8 g/reaction cycle. Table S2 in Supporting Information File 1 shows the power-controlled MW protocol, adjusted in respect to the small-scale procedure. Figure S3 reports a comparison between the temperature profiles of the small and large scale peracetylations: the two curves are comparable, even if a better stability is registered for the experiment performed in small scale. Nevertheless, the large-scale process successfully produced 10 g of peracetylated oleuropein in 65 minutes (see Supporting Information File 1).

## Conclusion

In conclusion, a solvent-free and catalyst-free MW-assisted method of peracetylation of natural polyols has been proposed. The method is high versatile such it can be applied to alcohols, phenols and polyols characterized by a huge chemical diversity and thermostability, simply tuning the MW settings. Such method is potentially universally applicable for green acetylation of hydrophilic biological molecules, thus improving their bioavailability and biological activity, no matter if they are simple polyphenols or glycosilated molecules. Thanks to the absolute absence of toxic reactants and byproducts the method is potentially easily scalable for industrial applications, including pharmaceutical, cosmetic and food industry, where the eco-compatibility of the reaction conditions, the easy waste management and the product safety are pivotal conditions for market.

## Experimental

### Materials and methods

MW-assisted reactions were performed in Synthos 3000 instrument from Anton Paar, equipped with a 64MG5 rotor and an IR probe as external control of the temperature. Using a temperature-controlled program the instrument is able to tune the power magnetron in order to reach and to maintain the fixed temperature throughout the experiment. For each run 16 positions of the rotor was occupied by 0.3–3 mL glass vials sealed with a dedicated PEEK screw-cup together with a reliable PTFE seal. Reactions were monitored by TLC using silica plates 60-F264 on alumina, commercially available from Merk. GC–MS spectra were recorded on a GC–MS Thermo Scientific workstation, formed by a Focus GC (30-m VARIAN-VF-5ms, 0.25 mm diameter capillary column, working on spitless mode, 1.2 mL/min He as carrier gas) and by an DSQ II mass detector. Chromatography was performed using a Thermo Scientific Dionex Ultimate 3000 RS, injecting directly onto a Thermo Scientific Hypersil Gold C18 column (50 × 2.1 mm, 1.9 µm particle size), equilibrated in 95% solvent A (0.1% aqueous solution of formic acid), 5% solvent B (methanol). The column and auto-sampler temperatures were maintained at 24 °C and 20 °C, respectively. The elution flow rate was 600 µL/min by linearly increasing the concentration of solvent B from 5 to 55% in 4 min, from 55% to 95% in 2 min and remaining for other 2 minutes in isocratic flow, then returning to 5% in 1 minute. At the end it was re-equilibrated for 3 minutes. The total run time, including column wash and equilibration was 15 min. A Thermo Scientific Q-ExactiveTM mass spectrometer was used for HRMS measurements using electrospray as ionization source with both negative and positive polarities, at a resolving power of 35.000 (defined as FWHM at *m*/*z* 200), IT = 100 ms, and ACG target = 500.000, by full scan analysis (mass range 140–900 amu for 2 samples and 200–1500 amu for other 2 samples). Source conditions were: spray voltage 2.9 kV, sheath gas: 30, arbitrary units, Auxiliary gas: 10, probe heater temperature: 280 °C; capillary temperature: 320 °C; S-Lens RF Level: 50. The instrument was calibrated by Thermo calibration solutions prior to the beginning the analysis. ^1^H and ^13^C spectra were recorded on a Bruker WM 300 instrument on samples dissolved in CDCl_3_. Chemical shifts are given in parts per million (ppm) from tetramethylsilane as the internal standard (0.0 ppm). Coupling constants (*J*) are given in Hertz. All new compounds were characterized by HRMS, ^1^H NMR and ^13^C NMR, while known compounds were analysed by comparison with the data coming from literature [14,45–47]. All chemicals were used as commercially available.

A request for an Italian Patent concerning the present protocol was submitted by the authors of this article (request number n° 102016000052914, registration date 23/05/2016).

### General procedure for Ac_2_O purification

The acetic anhydride (food grade, Eastman) was dried prior to use through the following procedure: a glass column under N_2_ was filled with 4 Å molecular sieves pre-activated at 350 °C overnight. The acetic anhydride was passed through the sieves 3 times and collected in a flask under N_2_. The collected anhydride was gently stirred over 20% w/w of activated molecular sieves for 48 hours, before the use.

### Optimized MW-assisted peracetylation

The substrate belonging to one of the subset reported in Table 1 (NTC, TC, CP, DGNP) (0.1 mmol) was left to react under MW heating (Synthos 3000, Anton Paar) with dry acetic anhydride (1 mL, 10 mmol) in a 3 mL vial (Rotor 64MG5 ), equipped with a magnetic stirrer in the presence of molecular sieves (10 % w/w). The microwave, equipped with IR sensor for external temperature control (IR limit calculated as follows: *T*_internal_ = 1.214 × *T*_IR_), has been set with the power programs provided for its subset as described in Table 1. At the end of the reaction, the mixture was filtered, diluted with ethanol (2 mL) and left under vigorous stirring for 30 minutes at 50 °C. The mixture was then evaporated under reduced pressure and a small amount of a saturated solution of sodium bicarbonate (3.8 mL, 10 mmol NaHCO_3_) was added. After the evolution of CO_2_, the precipitation of the peracetylated product was observed. The products were separated by simple decantation. For compounds which do not precipitate upon addition of NaHCO_3_, an extraction with AcOEt was needed. The organic phase, after drying with Na_2_SO_4_, filtration and evaporation, gave the reaction crude.

### Characterization of selected compounds

Peracetylated homovanillic alcohol (**5a**): Yellow oil; Yield 100%; MS (70 eV, IE) *m*/*z* (%): 252 [M^+^] (1), 210 [M^+^ − CH_2_=CH=O] (10), 192^+^ [M − CH_3_CO_2_H] (1), 150 [C_11_H_12_O_3_^+^ − CH_2_=CH=O] (100), 135 [C_9_H_9_O^+^ − CH_3_] (20); ^1^H NMR (300 MHz, CDCl_3_, 25 °C, TMS) δ 6.97 (d, *J*_G-H_ = 8 Hz, 1H, H_H_), 6.81 (s, 1H, H_F_), 6.80–6.78 (d, *J* = 8 Hz, 1H, H_G_), 4.31–4.25 (t, *J*_D-E_ = 7 Hz, 1H, H_D_), 3.82 (s, 3H, H_B_), 2.95–2.89 (t, *J*_D-E_ = 7 Hz, 1H, H_E_), 2.30 (s, 3H, H_A_), 2.03 (s, 3H, H_C_); ^13^C NMR (75 MHz, CDCl_3_, 25 °C, TMS) δ 171.3, 169.4, 151.3, 138.7, 137.0 123.0, 121.3, 113.4, 65.0 56.2, 35.3, 21.3, 21.0.

Acetyl salbutamol (**9a**): Yellow oil; inseparable mixture; pracetylated salbutamol (major product): Yield 30%; MS (70 eV, IE) *m*/*z* (%): 365 [M^+^] (0.5), 249 [M^+^ − CH_3_COOCH_3_-CH_2_=C=O] (10), 188 [M^+^ − 3 × CH_3_COO] (10), 146 [C_13_H_17_NO^+^ − *t*-Bu] (20), 86 [CH_2_=NH*t*-Bu^+^] (100); ^1^H NMR (300 MHz, CDCl_3_, 25 °C, TMS) δ 7.39 (d, *J*_G-I_ = 2 Hz, 1H, H_G_), 7.33–7.30 (dd, *J*_G-I_ = 2 Hz, *J*_H-I_ = 8.3 Hz, 1H, H_I_), 7.16–7.09 (d, *J*_H-I_ = 8.3 Hz, 1H, H_I_), 5.96–5.88 (dd, *J*_F-E_ = 3.4 Hz, *J*_F-E’_ = 10 Hz, 1H, H_F_), 3.84–3.72 (dd, *J*_E-E’_ = 16.3 Hz, *J*_F-E’_ = 10 Hz, 1H, H_E’_), 3.59–3.49 (dd, *J*_E-E’_ = 16.3 *J*_F-E_ = 3.4 Hz, 1H, H_E_), 2.34 (s, 3H, H_B_), 2.23 (br s, 1H, NH), 2.11 (s, 3H, H_C_), 2.08 (s, 3H, H_A_), 1.49 (s, 9H, H_D_); ^13^C NMR (75 MHz, CDCl_3_, 25 °C, TMS) δ 171.4, 170.9, 170.1, 169.5, 149.6, 136.3, 129.2, 129.1, 128.7, 127.8, 123.7, 61.5, 57.8, 51.2, 29.5, 25.8, 21.4, 21.2.

Acetylated Quercetin (**13a**): Yellow powder; inseparable mixture; di-*O*-acetylated quercetin (major product): Yield 60%; HRMS: [M + Na^+^] *m*/*z*: 451.0635 (theoretical [M + Na^+^] *m*/*z*: 451.0636); ^1^H NMR (300 MHz, CDCl_3_, 25 °C, TMS) δ 2.336 (s, 3H, Ac), 2.3381 (s, 3H, Ac), 2.3432 (s, 3H, Ac), 2.3483 (s, 3H, Ac), 6.86 (d, *J_meta_*= 2.19 Hz, 1H, H_E_), 7.34 (d, *J_meta_*= 2.19 Hz, 1H, H_A_), 7.37, (d, *J_ortho_*= 8.6 Hz, 1H, H_D_), 7.64 (d, *J_meta_*= 2.19 Hz, 1H, H_B_), 7.74 (dd,_, _*J_ortho_*= 8.6Hz, *J_meta_*= 2.19 Hz, 1H, H_C_); ^13^C NMR (75 MHz, CDCl_3_, 25 °C, TMS) δ 20.9, 21.0, 21.4, 21.5, 109.3, 114.2, 124.2, 124.3, 126.8, 128.14, 131.2, 142.6, 144.7, 150.8, 154.6, 157.2, 168.1, 168.2, 170.4.

Peracetylated α-hederine (**16a**) yellow oil: Yield 85%; HRMS: [M + Na^+^] *m*/*z*: 1025.5045 (theoretical [M + Na^+^] *m*/*z*: 1025.5080); ^1^H NMR (300 MHz, CDCl_3_, 25 °C, TMS) δ 0.73 (s, 3H, H_A_), 0.79 (s, 3H, H_B_), 0.90 (s, 3H, H_H_), 0.92 (s, 3H, H_L_), 0.95 (s, 3H, H_Q_), 1.10 (s, 6H, H_G,J’_), 1.23 (d, *J*_O-N_ = 6.6 Hz, 2H, H_O_), 1.29–1.90 (m, 20H, H_C,D,E,F,I,J,P,S,V,K,R_), 2.01 (s, 3H, Ac), 2.03 (s, 3H, Ac), 2.06 (s, 3H, Ac), 2.10 (s, 3H, Ac), 2.11 (s, 3H, Ac), 2.14 (s, 3H, Ac), 2.82 (m, 1H, H_U_), 3.85–3.98 (m, 2H, H_K_, H_E’_), 4.07–4.17 (m, 4H, 1H_E’_, H_B’,F’,L’_), 4.43 (d, *J*_N-O_ = 6.6 Hz, 2H, H_N_), 4.95–5.07 (m, 4H, H_C’,D’,G’,J’_), 5.22–5.30 (m, 3H, H_A’,T,I’_); ^13^C NMR (75 MHz, CDCl_3_, 25 °C, TMS) δ 182.8, 170.4, 170.3, 170.1, 170.0, 169.6, 143.6, 122.4, 103.5, 98.2, 81.9, 71.0, 69.5, 68.6, 67.8, 67.1, 62.6, 47.8, 46.4, 45.8, 41.9, 41.5, 41.0, 39.2, 38.3, 36.5, 33.7, 33.0, 32.4, 30.6, 27.5, 25.7, 25.4, 23.5, 23.4, 22.8, 20.9, 20.6, 17.9, 17.3, 16.9, 15.8, 12.6.

Peracetylated rutine (**17a**): Brown powder: Yield 45%; HRMS: [M + Na^+^] *m*/*z*: 1053.2470 (theoretical [M + Na^+^] *m*/*z*: 1053.2482); ^1^H NMR (300 MHz, CDCl_3_, 25 °C, TMS) δ 1.05 (d, *J*_L’K’_ = 6.25 Hz, 3H, H_L’_), 1.58 (s, 3H, Ac), 1.94 (s, 3H, Ac), 1.95 (s, 3H, Ac), 2.02 (s, 3H, Ac), 2.08 (s, 3H, Ac), 2.14 (s, 3H, Ac), 2.29 (s, 3H, PhOAc), 2.34 (s, 3H, PhOAc), 2.35 (s, 3H, PhOAc), 2.44 (s, 3H, PhOAc), 3.56–3.67 (m, 1H, H_E’_), 3.50–3.54 (m, 2H, H_F’’,J’_), 4.91–4.97 (m, 2H, H_I’,D’_), 5.05–5.09 (m, 2H, H_B’,H’_), 5.14–5.20 (m, 1H, H_C’_), 5.26 (d, *J*_F’F’’_ = 9.32 Hz, 1H, H_F’_), 5.42, (d, *J*_G’H’_ = 7.67 Hz, 1H, H_G’_), 5.41 (d, *J*_A’B’_ = 7.7 Hz, 1H, H_A’_), 6.84 (d, *J_meta_* = 2.19 Hz, 1H, H_E_), 7.31 (d, *J_meta_* = 2.19 Hz, 1H, H_A_), 7.35, (d, *J_ortho_* = 8.6 Hz, 1H, H_D_), 7.90 (d, *J_meta_*= 2.19 Hz, 1H, H_B_), 7.95 (dd_, _*J_ortho_* = 8.6 Hz, *J_meta_* = 2.19 Hz, 1H, H_C_); ^13^C NMR (75 MHz, CDCl_3_, 25 °C, TMS) δ 14.38, 17,52, 21.02 (×4), 21.42, 21.50, 23.33, 24.11, 29.27, 30.05, 30.72, 39.09, 66.68, 67.30, 68.51, 69.35, 69.72, 69.85, 71.24, 71.74, 72.90, 98.09, 99.94, 109.37, 113.77, 115.42, 123.80, 125.03, 127.57, 128.93, 137.28, 142.12, 144.44, 150.53, 154.30, 155.00, 156.96, 168.07, 168.21, 168.39, 169.59, 169.93, 170.07, 170.23, 170.41.

## Supporting Information

File 1 Scaled oleuropein peracetylation procedure, GC–MS, LC–HRMS, ^1^H and ^13^C NMR spectra of new compounds, as well as calculation for green chemistry metrics.
