# Supplementary material for: Tunable microwave-assisted method for the solvent-free and catalyst-free peracetylation of natural products
Source: Beilstein J Org Chem. 2016 Oct 20;12:2222–33. doi: 10.3762/bjoc.12.214 (PMC5082547; doi:10.3762/bjoc.12.214)
Supplement: File 1 — Scaled oleuropein peracetylation procedure, GC–MS, LC–HRMS, 1H and 13C NMR spectra of new compounds, as well as calculation for green chemistry metrics. [file Beilstein_J_Org_Chem-12-2222-s001.pdf]

Supporting Information

for

Tunable microwave-assisted method for the solvent-free and catalyst-free peracetylation of natural products

Manuela Oliverio*[1,2], Paola Costanzo[1] , Monica Nardi[3], Carla Calandruccio[1], Raffaele Salerno[2], and Antonio Procopio[1,2]

Address: [1]Department of Health Science, University Magna Graecia of Catanzaro, Viale Europa, Loc. Germaneto, 88100 Catanzaro, Italy, [2]InterRegional Center for Food Safety and Health, University Magna Graecia of Catanzaro, Viale Europa, Loc. Germaneto, 88100 Catanzaro, Italy and [3]Department of Chemistry, Università della Calabria, Cubo 12C, 87036-Arcavacata di Rende (CS), Italy

Email: Manuela Oliverio - m.oliverio@unicz.it.

*Corresponding author

Scaled oleuropein peracetylation procedure, GC–MS, LC–HRMS, ^{1}H and ^{13}C NMR spectra of new compounds, as well as calculation for green chemistry metrics

Table of contents

Experimental

Scale-up MW-assisted peracetylation of oleuropein **S3**

Scheme S1 – Table S1 **S3**

Characterization of new compounds **S4**

Green chemistry metrics **S6**

GC-EIMS spectra:

- Sample **3a** **S8**
- Sample **5a** **S9**
- Sample **6a** **S10**
- Sample **7a** **S11**
- Sample **8a** **S12**
- Sample **9a** **S13**
- Sample **10a** **S14**

LC/HRMS spectra:

- Sample **12a** **S15**
- Sample **13a** **S17**
- Sample **16a** **S19**
- Sample **17a** **S21**

^{1}H NMR spectra

- Sample **3a** **S23**
- Sample **5a** **S24**
- Sample **6a** **S25**
- Sample **7a** **S26**
- Sample **8a** **S27**
- Sample **9a** **S28**
- Sample **10a** **S29**
- Sample **12a** **S30**
- Sample **13a** **S31**
- Sample **16a** **S32**
- Sample **17a** **S33**

^{13}C NMR spectra

- Sample **3a** **S34**
- Sample **5a** **S35**
- Sample **6a** **S36**
- Sample **7a** **S37**
- Sample **8a** **S38**
- Sample **9a** **S39**
- Sample **10a** **S40**
- Sample **12a** **S41**
- Sample **13a** **S42**
- Sample **16a** **S43**
- Sample **17a** **S44**

References **S44**

Experimental

Scale-up MW-assisted peracetylation of oleuropein

For scale up reaction 8 positions of the XF100 rotor were occupied with 100 mL PTFE-TFM vessel, loaded in a ceramic pressure jacket sealed with a dedicated PTFE-TFM cap with integrated relief valve and safety disk, fixed in a PEK seal holder.

Oleuropein (1 g) was left to react under MW heating with dry acetic anhydride (10 mL) in a 100 mL vial (rotor XF-100), equipped with a magnetic stirrer in the presence of molecular sieves (10% w/w). The microwave oven, equipped with IR sensor for external temperature control has been set with the power program provided as described in Table S1.

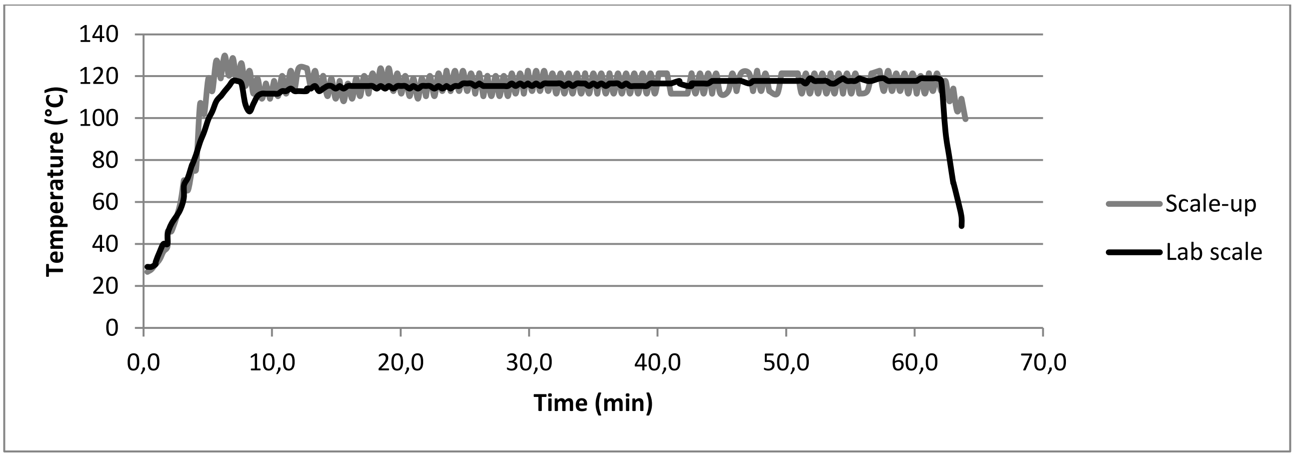

Figure S1: Temperature profiles in Scale-up and lab scale reactions

Table S1: P-controlled MW program (Synthos 3000, equipped with XF-100 rotor) for the scaled peracetilation of oleuropein

Time (min)	*P* (W)	*T* (°C) [a]	IR limit (°C)	Yield (%)
0→5	0→350	0→110	105	100
5→10	350	110		
10→12	350→400	110→120		
12→62	400	120		
62→65	0	120→50		

[a]Calculated as follows: $T_{internal} = 1.214 \times T_{IR}$

N-acetyl anthranilic acid (**1a**) Yellow powder; Yield 100%; ^{1}H NMR and ^{13}C NMR data were compared with those reported in a spectra Database [1].

Acetyl Salicylic acid (**2a**) White powder; Yield 100%; ^{1}H-NMR and ^{13}C-NMR data were compared with those reported in a spectra Database [1].

O-acetyl cholesterol (**3a**) White powder; Yield 62%; MS (70 eV, IE): m/z (%) = 368 [M^+-CH_3COO] (100), 353 [M^+- CH_3COOCH_3] (30), 247 [M^+- CH_3COOCH_3–C_9H_{13}] (30), 147 [$C_{11}H_{15}^+$] (100), 133 [$C_{11}H_{15}^+$-CH_2] (45), 119 [$C_{11}H_{15}^+$-2xCH_2] (50), 105 [$C_{11}H_{15}^+$-3xCH_2] (70), 91 [$C_{11}H_{15}^+$-4xCH_2] (60); ^{1}H NMR (300MHz, $CDCl_3$, 25°C, TMS) δ: 5.40-5.35 (br d, J=4,8 Hz, 1H, H_A), 4.67-4.54 (m, 1H, H_B), 2.36-2.28 (br d, J=7,7 Hz, 2H, H_C), 2.04 (s, 3H, H_D), 2.02-0.83 (m, 38H), 0.67 (s, 3H, H_E); ^{13}C NMR (300MHz, $CDCl_3$, 25°C, TMS) δ: 170.2, 140.2, 122.7, 57.2, 56.8, 50.7, 42.8, 40.2, 39.8, 38.5, 37.4, 37.0, 36.6, 36.0, 32.4, 32.2, 28.3, 28.2, 28.1, 24.5, 24.2, 22.8, 22.6, 21.4, 21.2, 19.4, 19.0, 12.1, 10.3.

Peracetylated Hydroxytyrosol (**4a**) Yellow Oil; Yield 100%; ^{1}H-NMR and ^{13}C-NMR data were compared with those reported in literature [2].

O-acetyl citronellol (**6a**) Yellow Oil; Yield 100%; MS (70 eV, IE): m/z (%) = 198 [M^+] (1), 138 [M^+-CH_3CO_2H] (40), 123 [$C_{10}H_{18}^+$–CH_3] (60), 109 [$C_9H_{15}^+$–CH_2] (30), 95 [$C_9H_{15}^+$–2xCH_2] (98), 81 [$C_9H_{15}^+$–3xCH_2] (100), 69 [$C_9H_{15}^+$–C_5H_9] (98);^{1}H NMR (300MHz, $CDCl_3$, 25°C, TMS) δ: 5.14-5.04 (br t, J_{CD}=6 Hz, 1H, H_C), 4.18-4.02 (m, 2H, H_E), 2.04 (s, 3H, H_L), 2.04-1.87 (m, 2H, H_D), 1.68 (s, 3H, H_B), 1.60 (s, 3H, H_A), 1.60-1.10 (m, 5H, H_F,H_I), 0.88-0.9 (d, J_{GF}=6 Hz 3H, H_G); ^{13}C NMR (300MHz, $CDCl_3$, 25°C, TMS) δ: 171.7, 135.6, 129.9, 82.4, 64.9, 63.2, 39.9, 35.8, 35.6, 35.3, 35.2, 33.8, 30.2, 30.0, 29.8, 26.7, 26.6, 25.2, 24.6, 21.3, 19.8, 19.7, 19.0.

O-acetyl paracetamol (**7a**) White powder; Yield 93%; MS (70 eV, IE): m/z (%) = 193 [M^+] (10), 151 [M^+–CH_2=C=O] (50), 109 [$C_8H_9NO_2^+$–CH_3CO] (100), 80 [$C_6H_6NO^+$-CHO] (10); ^{1}H NMR (300MHz, $CDCl_3$, 25°C, TMS) δ: 7.62-7.52 (br s, 1H, NH), 7.51-7.43 (d, J_{ortho}=8,8 Hz, 2H, H_C), 6.96-7.06 (d, J_{ortho}=8,8 Hz, 2H, H_D), 2.29 (s, 3H, H_B), 2.18 (s, 3H, H_A); ^{13}C NMR (300MHz, $CDCl_3$, 25°C, TMS) δ: 170.0, 168.6, 147.2, 135.9, 122.3, 121.2, 24.8, 21.4.

N-Boc-*O*-acetyl tyrosine methyl ester (**8a**) White powder; Yield 95%; MS (70 eV, IE): m/z (%) = 337 [M^+] (1), 264 [M^+-O*t*-But] (10), 219 [M^+-2x$COOCH_3$] (15), 178 [M^+-$COCH_3$-NH_2COO*t*-But] (100), 107 [CH_3PhO^+] (45); ^{1}H NMR (300MHz, $CDCl_3$, 25°C, TMS) δ: 7.13 (s, 2H, H_G), 7.03 (s, 2H, H_F), 5.1-4.8 (br s, 1H, NH), 4.7-4.3 (br s, 1H_D), 3.71 (s, 3H, H_A), 3.2-2.8 (br s, 2H, H_E), 2.29 (s, 3H, H_C), 1.42 (s, 9H, H_B); ^{13}C NMR (300MHz, $CDCl_3$, 25°C, TMS) δ: 172.5, 169.7, 155.4, 150.0, 134.0, 130.6, 121.9, 80.3, 54.7, 52.6, 38.0, 28.6, 21.4.

O-acetyl myrtenol (**10a**) Yellow Oil; Yield 100%; MS (70 eV, IE): m/z (%) = 152 [M^+-CH_2COH] (1), 119 [M^+-CH_2COH-CH_3] (40), 91 [119^+-CH_2=CH_2] (100); ^{1}H NMR (300MHz, $CDCl_3$, 25°C, TMS) δ: 5.59 (m, 1H, H_C), 4.51-4.36 (m, 2H, H_B), 2.45-2.08 (m, 5H, $H_{F,D,D',E,G}$), 2.05 (s, 3H, H_A), 1.29 (s, 1H, H_H), 1.21-1.16 (d, $J_{E\text{-}F'}$=8.7 Hz, 1H, $H_{F'}$), 0.84 (s, 3H, $H_{H'}$); ^{13}C NMR (300MHz, $CDCl_3$, 25°C, TMS) δ: 171.4, 143.3, 121.8, 67.4, 43.9, 41.1, 38.4, 21.8, 31.6, 26.5, 21.3.

Peracetylated Methyl-α-D-glucopyranoside (**11a**) White powder; Yield 70%; ^{1}H-NMR and ^{13}C-NMR data were compared with those reported in literature [3].

Triacetyl Uridine (**12a**) Yellow Oil; Yield 92%; HRMS: [M+Na^{+}] m/z 393.0902 (theoretical [M+Na^{+}] m/z 393.0904); ^{1}H NMR (300MHz, $CDCl_3$, 25°C, TMS) δ: 7.43-7.38 (d, $J_{A\text{-}B}$=8.2 Hz, 1H, H_A), 6.08-6.02 (d, $J_{C\text{-}D}$=4.8 Hz, 1H, H_C), 5.83-5.77 (d, $J_{A\text{-}B}$=8.2 Hz, 1H, H_B), 5.38-5.29 (m, 2H, H_G), 4.36-4.30 (m, 3H, $H_{D,E,F}$), 2.15 (s, 3H, H_H), 2.14 (s, 3H, H_I), 2.11 (s, 3H, H_L); ^{13}C NMR (300MHz, $CDCl_3$, 25°C, TMS) δ: 170.1, 169.5, 162.4, 150.4, 139.5, 103.6, 88.3, 80.5, 73.1, 70.7, 63.3 20.7, 20.4 20.3.

Peracetylated Oleuropein (**14a**) Yellow powder; Yield 100%; MS, ^{1}H-NMR e ^{13}C-NMR data were compared with those reported in literature [2].

Peracetylated β-D-lactose (**15a**) White powder; Yield 50%; ^{1}H-NMR and ^{13}C-NMR data were compared with those reported in literature [4].

Green chemistry metrics

Atom Economy (AE) and Reaction Mass Efficiency (RME) were used to evaluate the greenness of the procedure, calculated according to the report of Constable et al.[5]:

Atom economy (AE)= $\frac{FW\ all\ reaction\ products}{FW\ all\ reaction\ reagents}$ x100

Reaction Mass Efficiency (RME)= $\frac{Mass\ of\ all\ reaction\ products}{Mass\ of\ all\ reaction\ reagents}$ x100

The general reaction took into account was:

$$R\text{-}(OH)_n + n\ Ac_2O \longrightarrow R\text{-}(OAc)_n + n\ AcOH$$

Were n is the stoichiometric coefficient between reagents.

The calculations are reported in Table S2.

Table S2

FW reag tot (FW AA X stoch)	FW perac phenol	AE	yield	RME
239	179	75	1	75
240	180	75	1	75
488	428	88	0,62	54,56
460	280	61	1	61
372	252	68	1	68
258	198	77	0,55	42,35
253	193	76	0,93	70,68
397	337	85	0,9	76,5
545	365	67	0,3	20,1
254	194	76	1	76
704	362	51	0,7	35,7
550	370	67	0,92	61,64
506	386	76	0,6	45,6
1050	792	75	1	75
1158	678	58	0,5	29
1363	961	70	0,85	59,5
1630	1030	63	0,45	28,35

For the calculation of mass intensity and mass productivity, the work-up used in the general reaction was considered as a part of the full process. In this case the general reaction equation is composed by the following three steps:

a $R\text{-}(OH)_n + \text{exc } Ac_2O \longrightarrow R\text{-}(OAc)_n + (\text{exc-n})\ Ac_2O$

b $(\text{exc-n})\ Ac_2O + EtOH \longrightarrow (\text{exc-n})\ AcOEt + (\text{exc-n})\ AcOH$

c $(\text{exc-n})\ AcOH + NaHCO_3 \longrightarrow (\text{exc-n})\ NaOAc + H_2O + CO_2$

Peracetylated phenol, EtOAc and NaOAc were considered as useful products, while phenol, excess of acetic anhydride, EtOH and $NaHCO_3$ were included in the total mass calculation respectively as reagents, solvent and reaction auxiliaries, and used in the following equations:

Mass Intensity (MI)= $\frac{Mass\ of\ all\ chemicals\ used}{Mass\ of\ all\ reaction\ products}$

Mass Productivity (MP)= $\frac{1}{Mass\ Intensity}$ x100

Table S3 summarizes the calculations of the previous parameters.

Table S3

Phenol mass	ecc AA mass (reag + solv)	auxiliary mass	perac phenol mass	EtOAc mass	NaOAc mass	MI	MP
13,7	1080	2440	17,9	871,2	811,8	2	50
13,8	1080	2440	18	871,2	811,8	2	50
38,6	1080	2440	26,536	540,144	503,316	3	33
15,4	1080	2440	28	853,6	795,4	2	50
16,8	1080	2440	25,2	862,4	803,6	2	50
15,6	1080	2440	10,89	479,16	446,49	3	33
15,1	1080	2440	17,949	810,216	754,974	2	50
29,5	1080	2440	30,33	784,08	730,62	2	50
23,9	1080	2440	10,95	256,08	238,62	7	14
15,2	1080	2440	19,4	871,2	811,8	2	50
19,4	2160	2440	25,34	585,2	545,3	3	33
24,4	2160	2440	34,04	785,312	731,768	2	50
30,2	1080	2440	23,16	517,44	482,16	3	33
54	1080	2440	79,2	836	779	2	50
34,2	2160	2440	33,9	404,8	377,2	5	20
75,1	2160	2440	81,685	703,12	655,18	3	33
61	2160	2440	46,35	356,4	332,1	6	16

GC-EIMS spectra

Sample **3a**

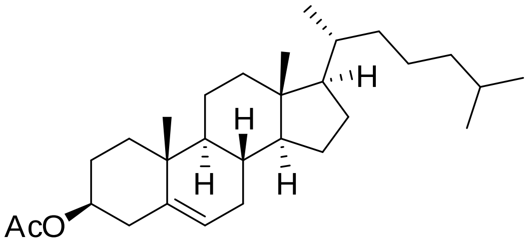

3a

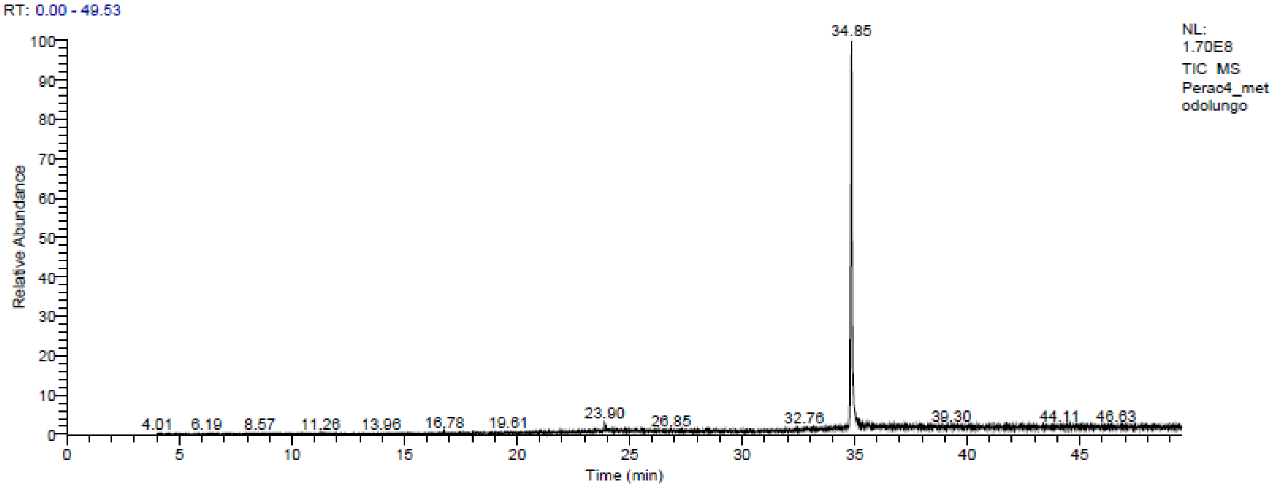

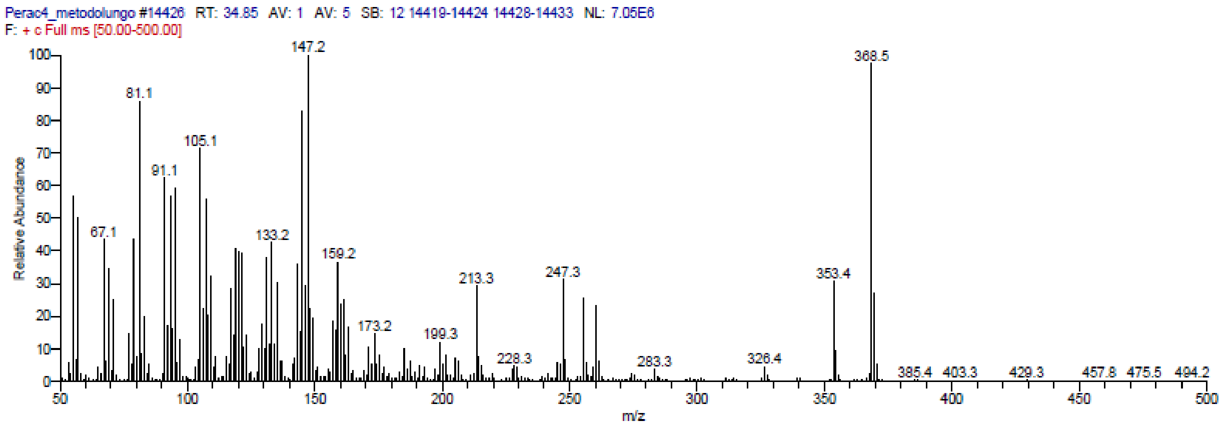

Sample **5a**

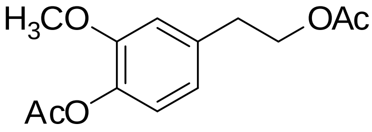

H3CO
OAc
AcO

5a

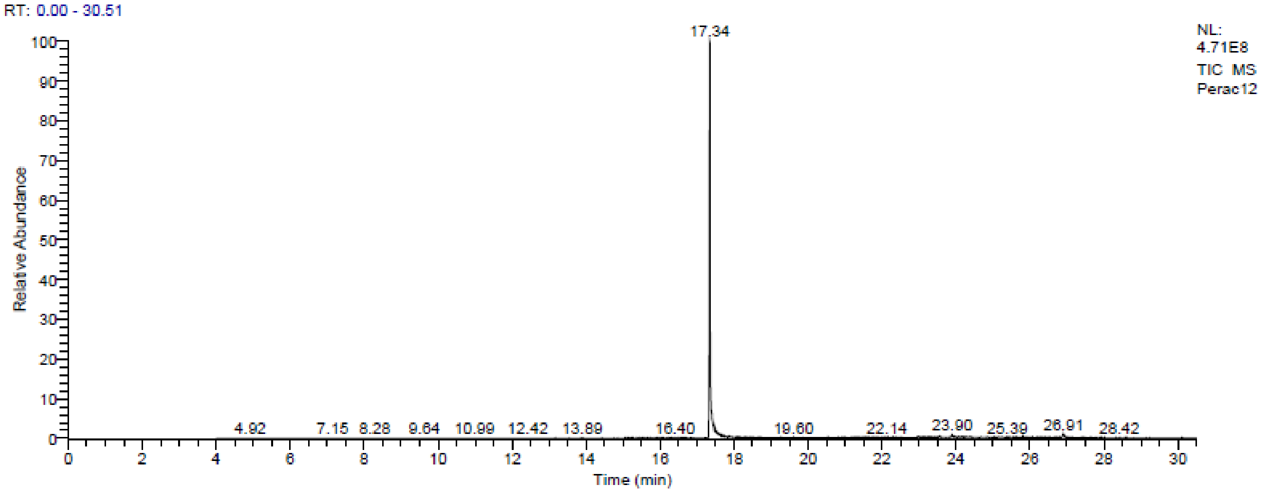

RT: 0.00 - 30.51
17.34
NL:
4.71E8
TIC MS
Perac12
Relative Abundance
4.92
7.15
8.28
9.64
10.99
12.42
13.89
16.40
19.60
22.14
23.90
25.39
26.91
28.42
Time (min)

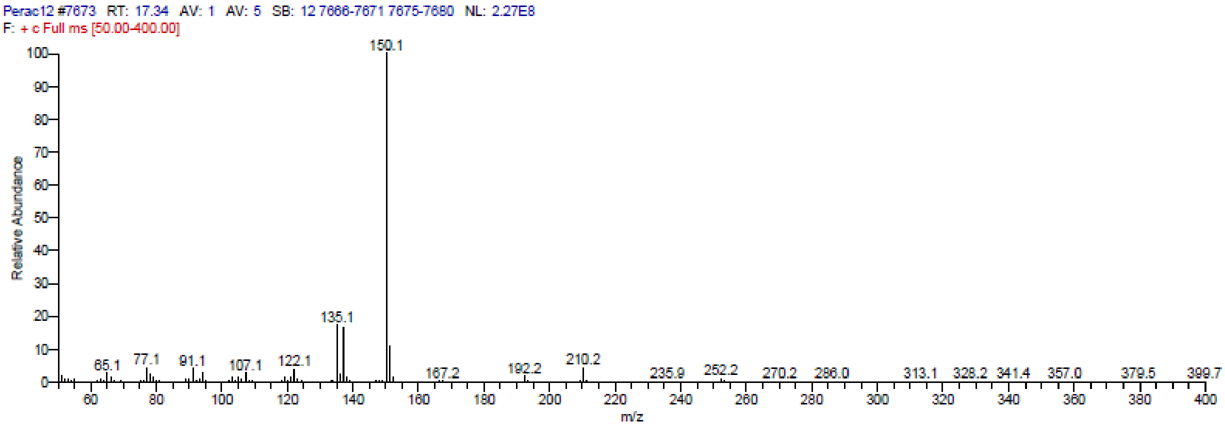

Perac12 #7673 RT: 17.34 AV: 1 AV: 5 SB: 12 7666-7671 7675-7680 NL: 2.27E8
F: + c Full ms [50.00-400.00]
150.1
Relative Abundance
135.1
65.1
77.1
91.1
107.1
122.1
167.2
192.2
210.2
235.9
252.2
270.2
286.0
313.1
328.2
341.4
357.0
379.5
399.7
m/z

Sample **6a**

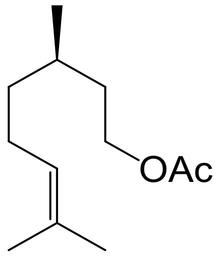
OAc

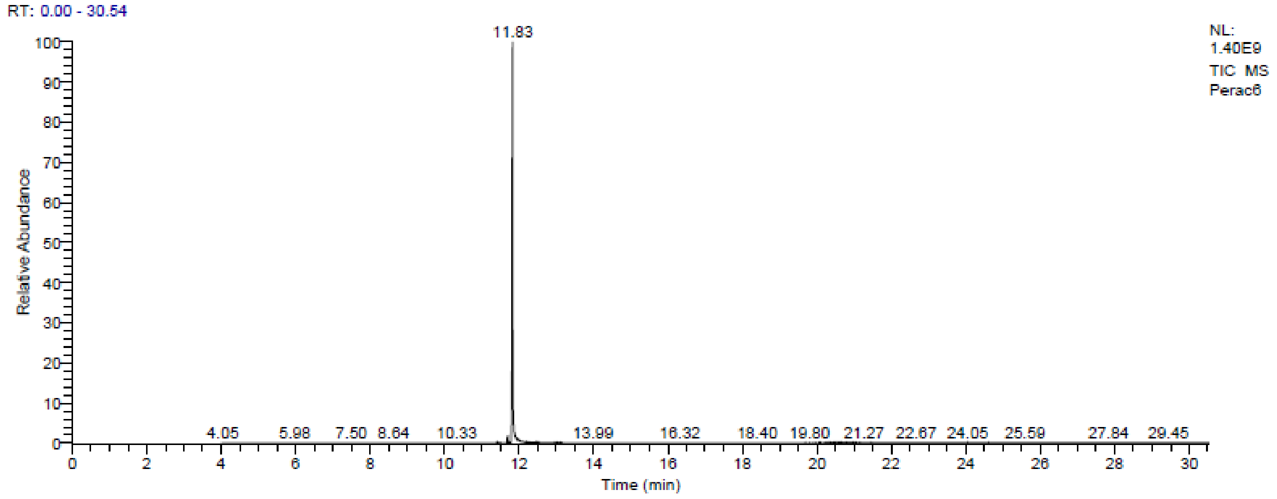
RT: 0.00 - 30.54
11.83
NL:
1.40E9
TIC MS
Perac6
Relative Abundance
4.05 5.98 7.50 8.64 10.33 13.99 16.32 18.40 19.80 21.27 22.67 24.05 25.59 27.84 29.45
Time (min)

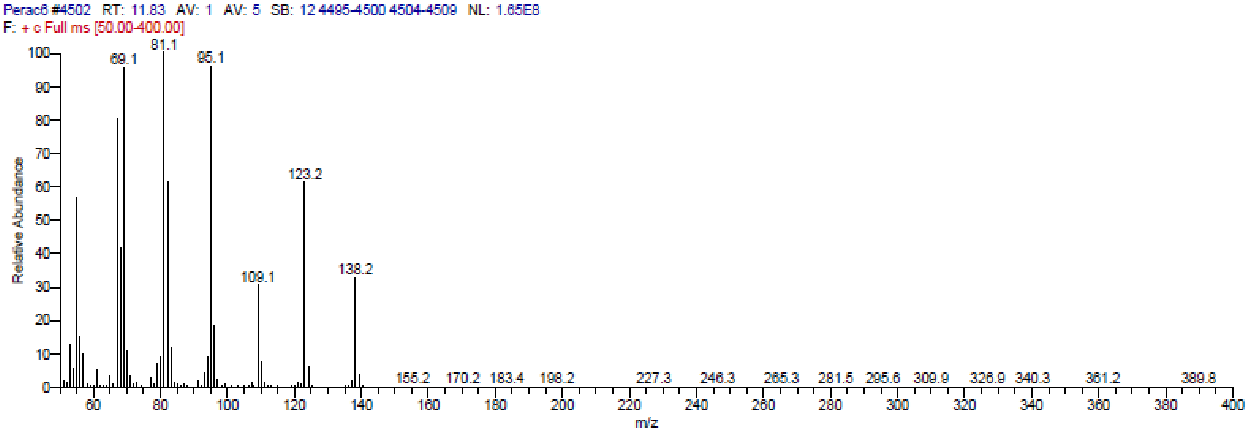
Perac6 #4502 RT: 11.83 AV: 1 AV: 5 SB: 12 4495-4500 4504-4509 NL: 1.65E8
F: + c Full ms [50.00-400.00]
69.1
81.1
95.1
123.2
109.1
138.2
155.2 170.2 183.4 198.2 227.3 246.3 265.3 281.5 295.6 309.9 326.9 340.3 361.2 389.8
Relative Abundance
m/z

Sample **7a**

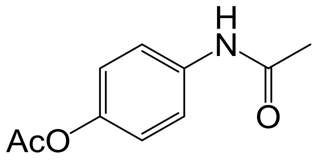
H
N
O
AcO

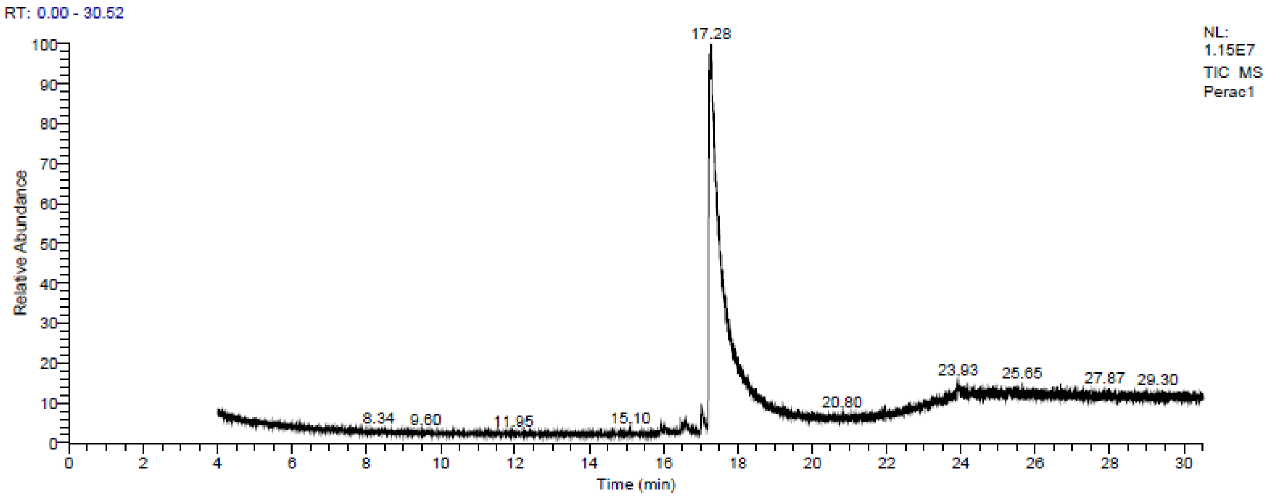
RT: 0.00 - 30.52
NL:
1.15E7
TIC MS
Perac1
17.28
8.34
9.60
11.95
15.10
20.80
23.93
25.65
27.87
29.30
Relative Abundance
Time (min)

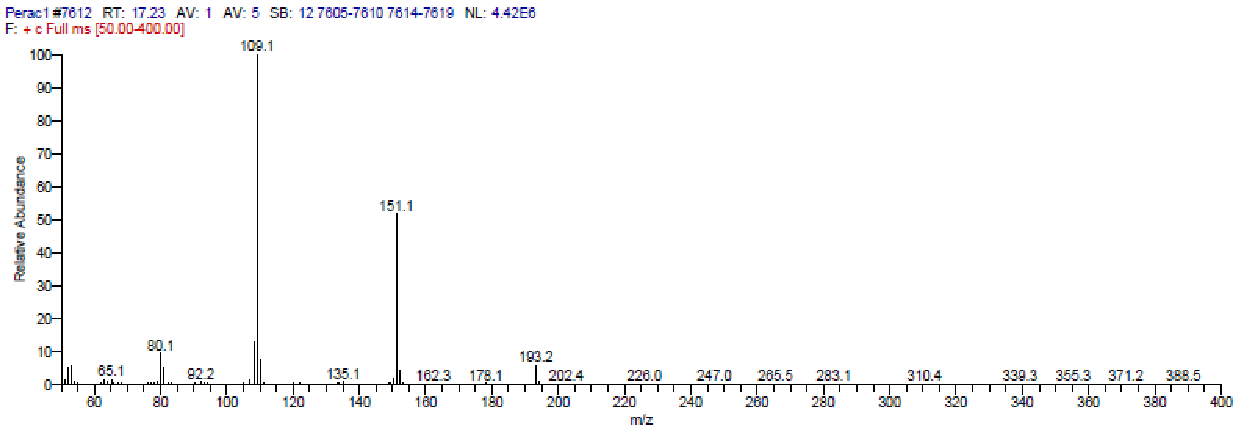
Perac1 #7612 RT: 17.23 AV: 1 AV: 5 SB: 12 7605-7610 7614-7619 NL: 4.42E6
F: + c Full ms [50.00-400.00]
109.1
151.1
80.1
65.1
92.2
135.1
162.3
178.1
193.2
202.4
226.0
247.0
265.5
283.1
310.4
339.3
355.3
371.2
388.5
Relative Abundance
m/z

Sample **8a**

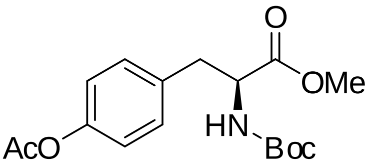

Data File:	peracet_3_1-10
Operator:	ITQ
Acquisition Date:	02/27/15 12:04:59 PM
Run Time(min):	26.50
Instrument Method:	C:\Xcalibur\METODI\Glicerol\ecocontrol_400
Current Processing Method:	N/A

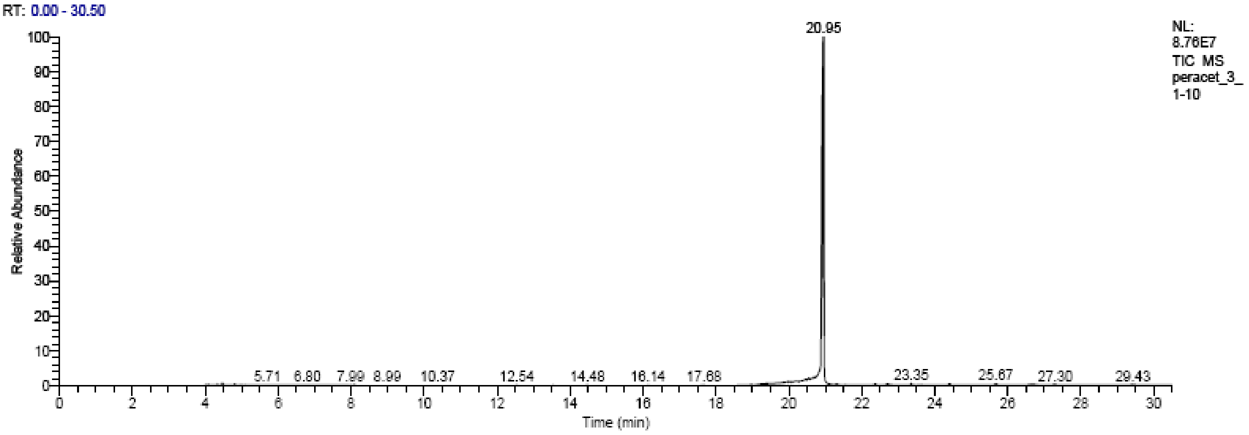

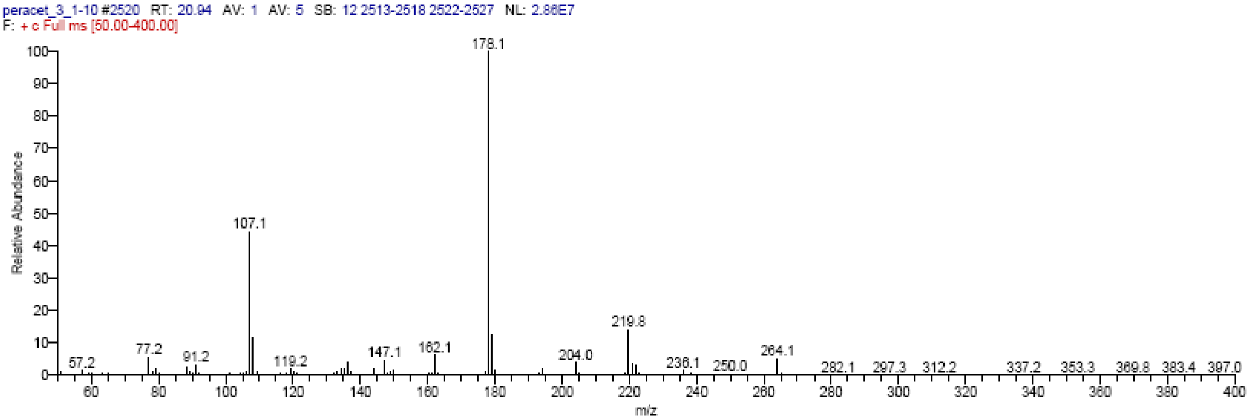

Sample **9a**

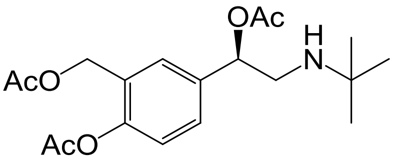

(***major product***)

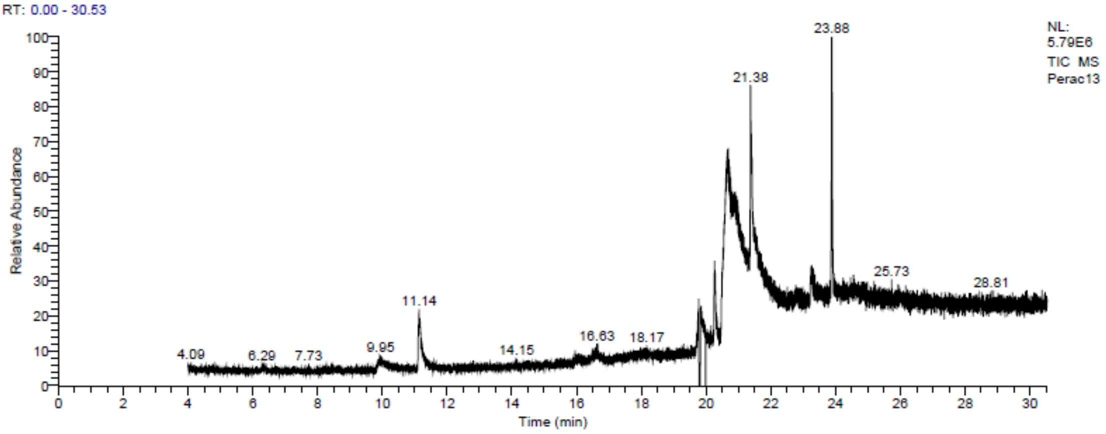

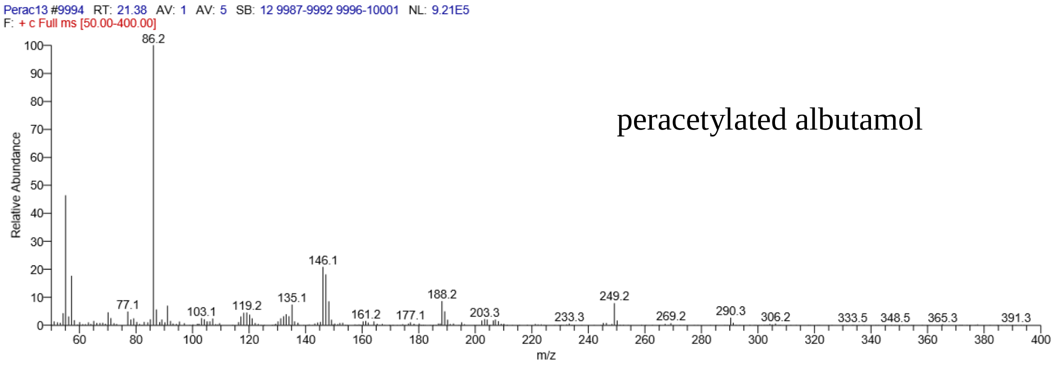

peracetylated albutamol

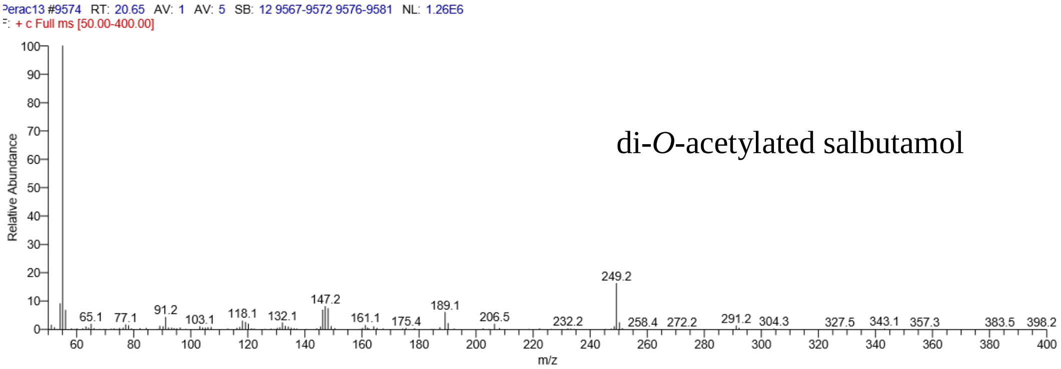

di-*O*-acetylated salbutamol

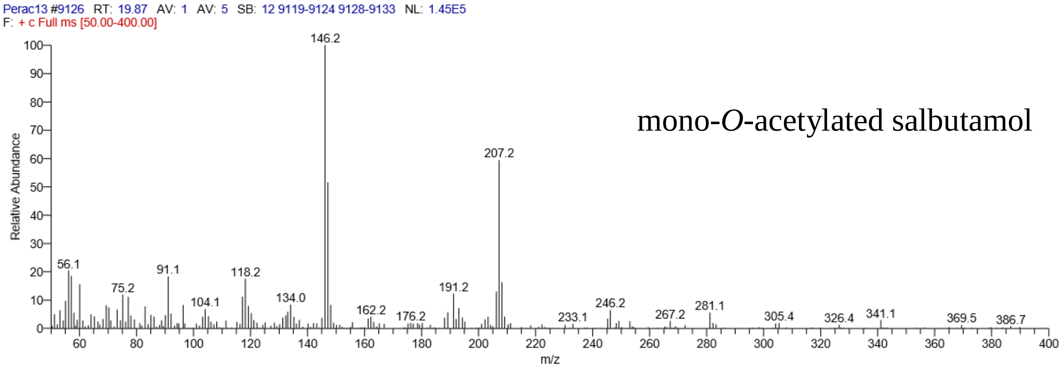

mono-*O*-acetylated salbutamol

Sample **10a**

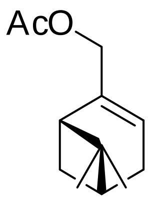
AcO

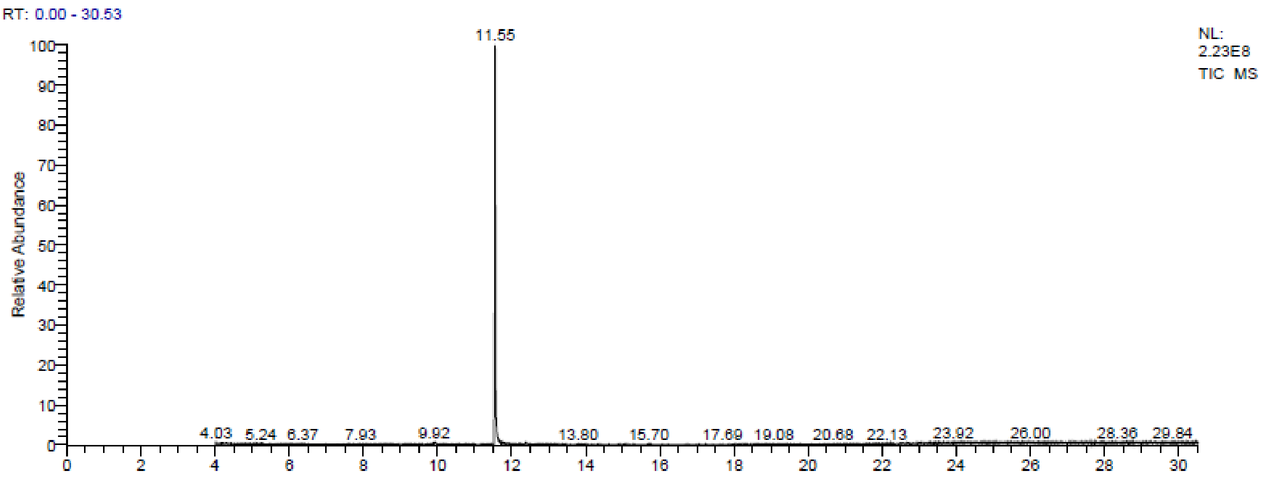
RT: 0.00 - 30.53
NL:
2.23E8
TIC MS
11.55
4.03
5.24
6.37
7.93
9.92
13.80
15.70
17.69
19.08
20.68
22.13
23.92
26.00
28.36
29.84
Relative Abundance

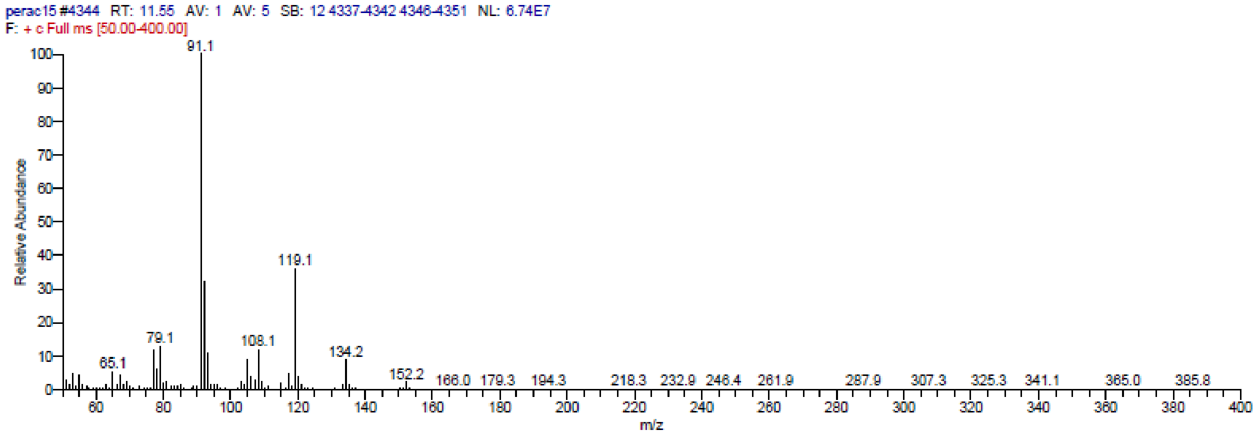
perac15 #4344 RT: 11.55 AV: 1 AV: 5 SB: 12 4337-4342 4346-4351 NL: 6.74E7
F: + c Full ms [50.00-400.00]
91.1
119.1
79.1
108.1
134.2
65.1
152.2
166.0
179.3
194.3
218.3
232.9
246.4
261.9
287.9
307.3
325.3
341.1
365.0
385.8
Relative Abundance
m/z

LC/HRMS spectra

Sample **12a**

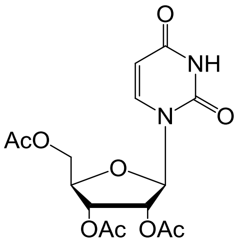

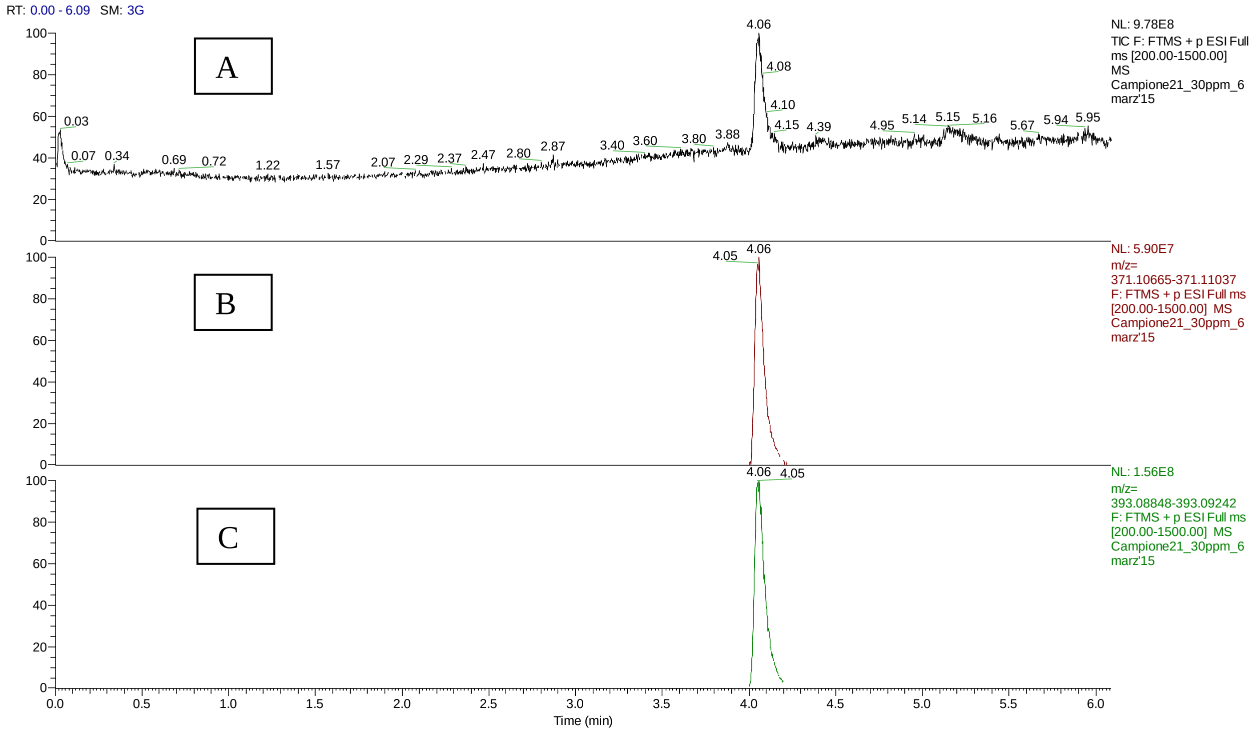

LCHRMS (*m/z*) spectrum of peracetylated uridine (isolated product) in total ion current (TIC, entry A) and extract ion current (XIC,B $[M + H]^+$) ,C ($[M + Na]^+$)).

HRMS (m/z, $[M + Na]^+$; $[M + H]^+$ accuracy (ppm)) spectra of peracetylated uridine:

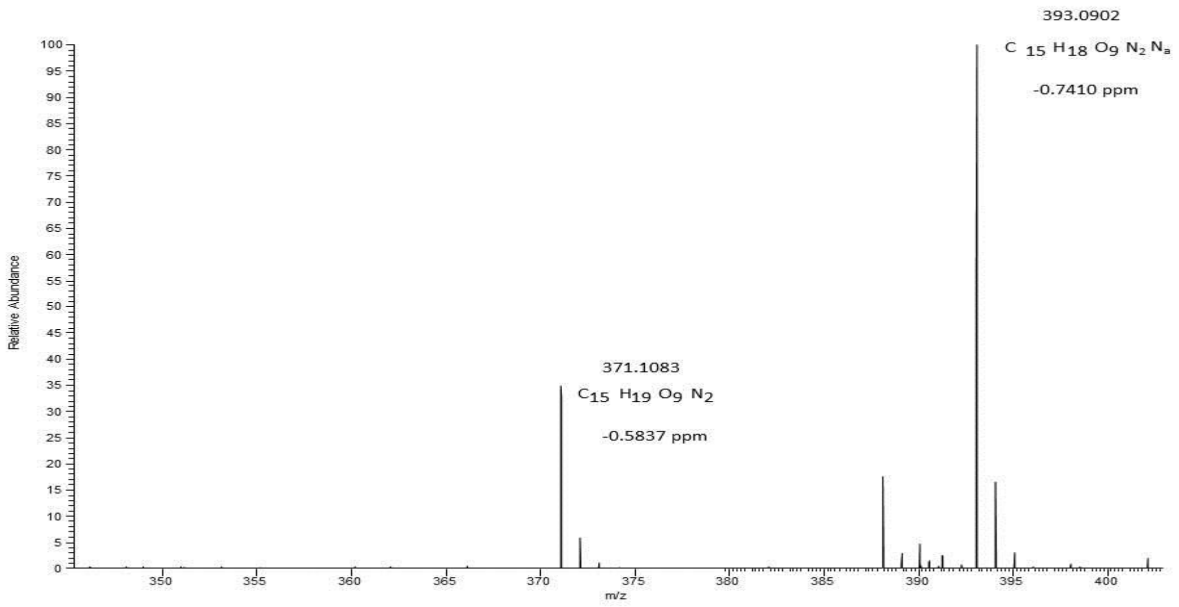

Peracetylated uridine: experimental value $[M + Na]^+$ m/z 393.0902 (theoretical $[M + Na]^+$ m/z 393.0904). and $[M + H]^+$ m/z 371.1083 (theoretical $[M + H]^+$ m/z 371.1085).

Sample **13a**

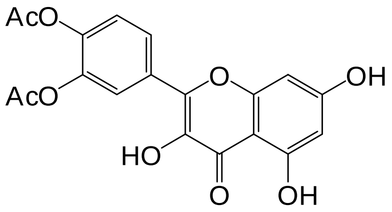

(***major product***)

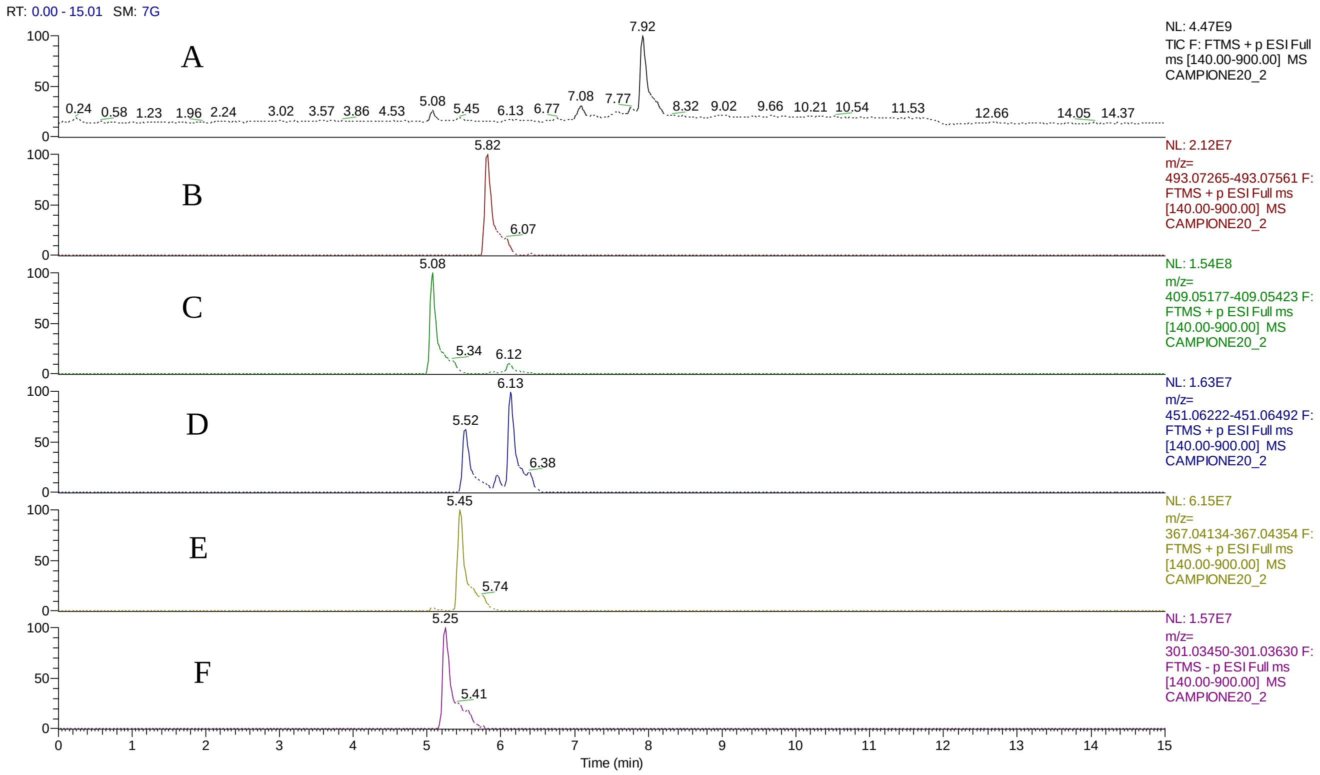

LCHRMS (*m*/*z*, $[M + Na]^+$ and $[M - H]^-$ only for entry F) spectrum of *O*-acetylated quercetin (reaction mix) in total ion current (TIC, entry **A**) and extract ion CURRENT (XIC, entry **B**–**F**) relative to main acetylated forms: tetra-*O*-acetylated quercetin (8% of the mix, entry **B**), di-*O*-acetylated-quercetin (60% of the mix, entry **C**), tri-*O*-acetylated quercetin (7% of the mix, entry **D**), mono-*O*-acetylated quercetin (25% of the mix, entry **E**). The conversion was estimated around 96%, because of the presence of 6% of unreacted quercetin (entry **F**).

HRMS (m/z, $[M+Na]^+$; accuracy (ppm)) spectra of *O*-acetylated Quercetin:

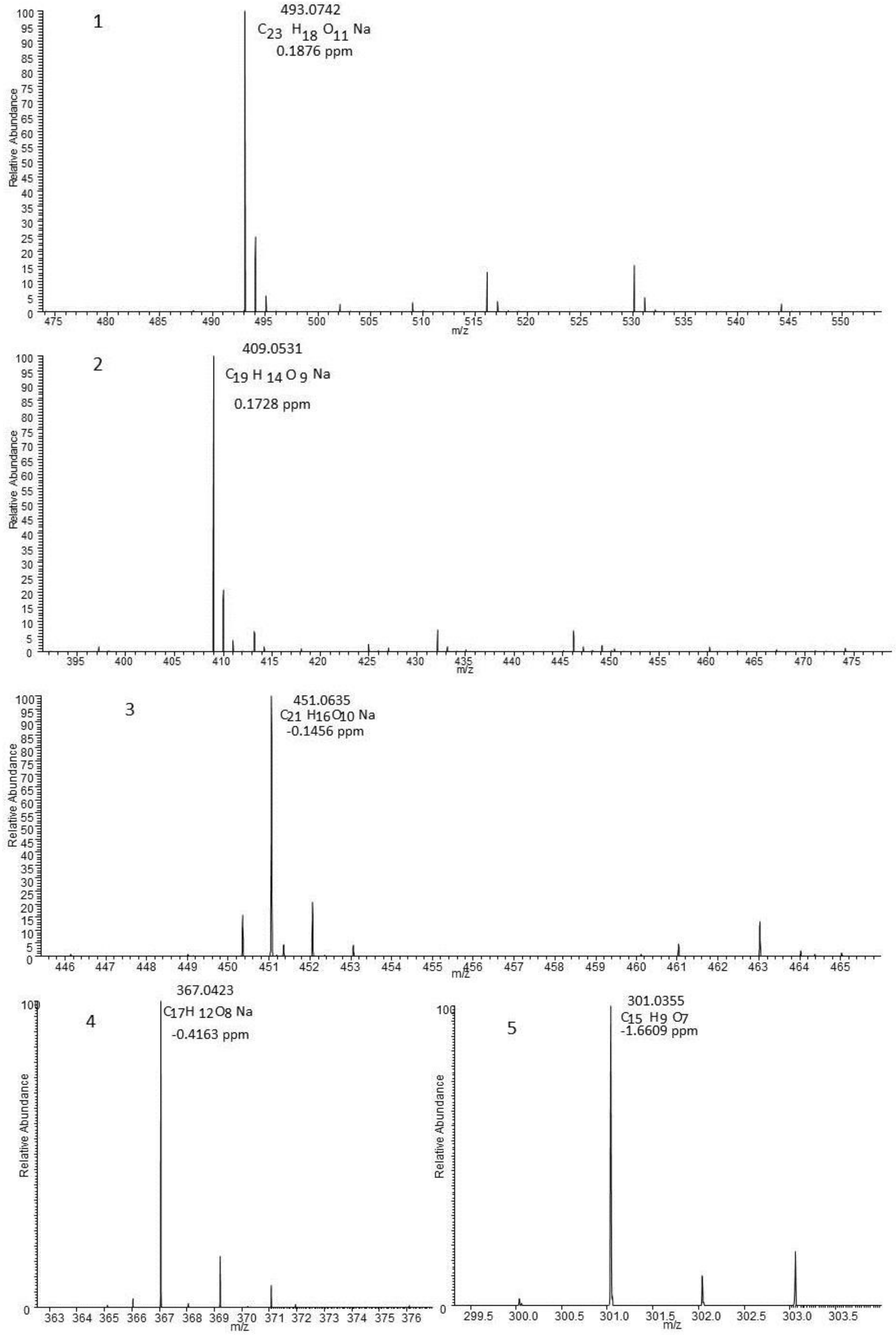

(**1**) Tetra-*O*-acetylated quercetin: experimental value $[M + Na]^+$ *m*/*z* 493.0742 (theoretical $[M + Na]^+$ *m*/*z* 493.0741) (**2**) Di-*O*-acetylated-quercetin: experimental value $[M + Na]^+$ *m*/*z* 451.0635 (theoretical $[M + Na]^+$ *m*/*z* 451.0636) (**3**) Tri-*O*-acetylated quercetin: experimental value $[M + Na]^+$ *m*/*z* 451.0635 (theoretical $[M + Na]^+$ *m*/*z* 451.0636) (**4**) Mono-*O*-acetylated quercetin: experimental value $[M + Na]^+$ *m*/*z* 367.0423 (theoretical $[M + Na]^+$ *m*/*z* 367.04244) (**5**) Quercetin: experimental value $[M - H]^-$ *m*/*z* 301.

Sample **16a**

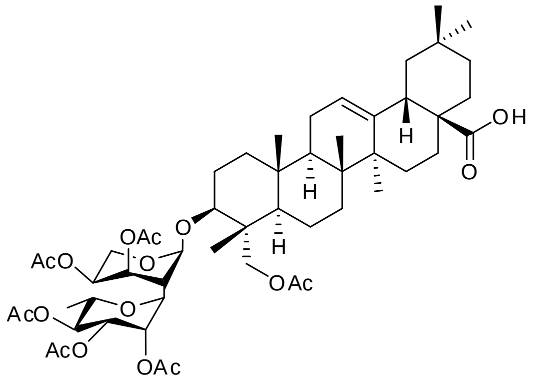

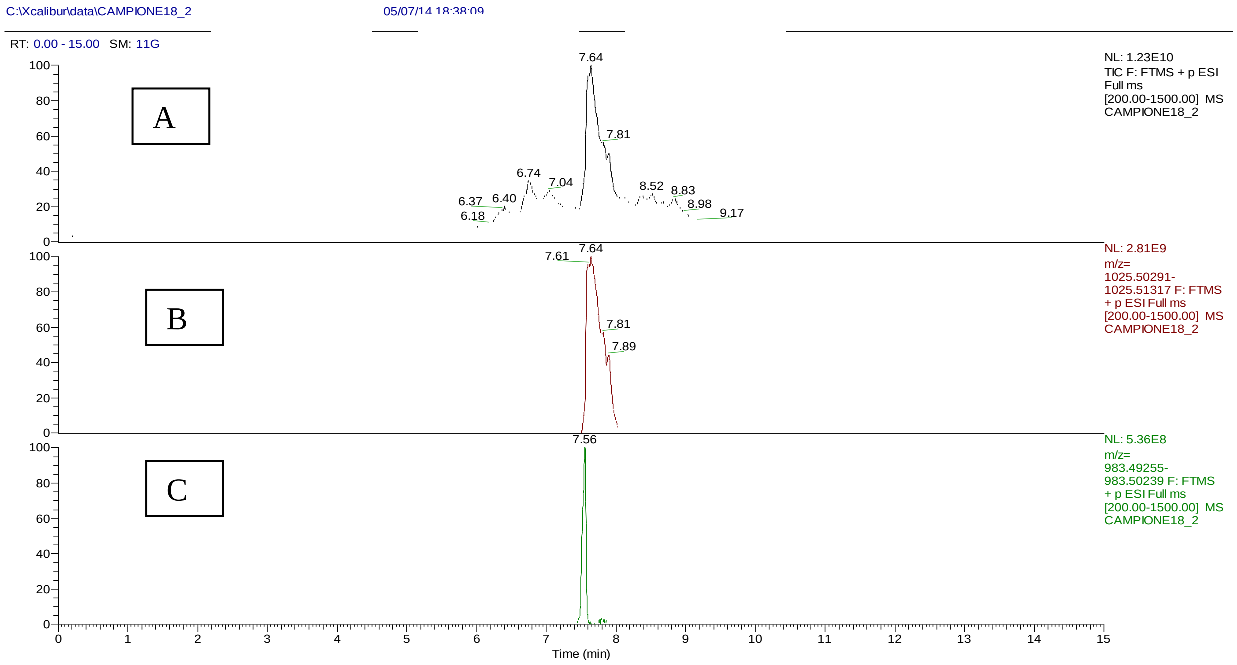

LCHRMS (*m*/z, [M + Na]$^+$) spectrum of *O*-acetylated α-ederin (reaction mix) in total ion current (TIC, entry **A**) and extract ion current (XIC, entry **B**,**C**) relative to main acetylated-forms: Esa-*O*-acetylated α-ederin (85% of the mix, entry **B**), penta-*O*-acetylated α-ederin (15% of the mix, entry **C**).

HRMS (*m*/*z*, [M + Na]$^+$; R = operative resolution; accuracy (ppm)) spectra of *O*-acetylated α-ederin:

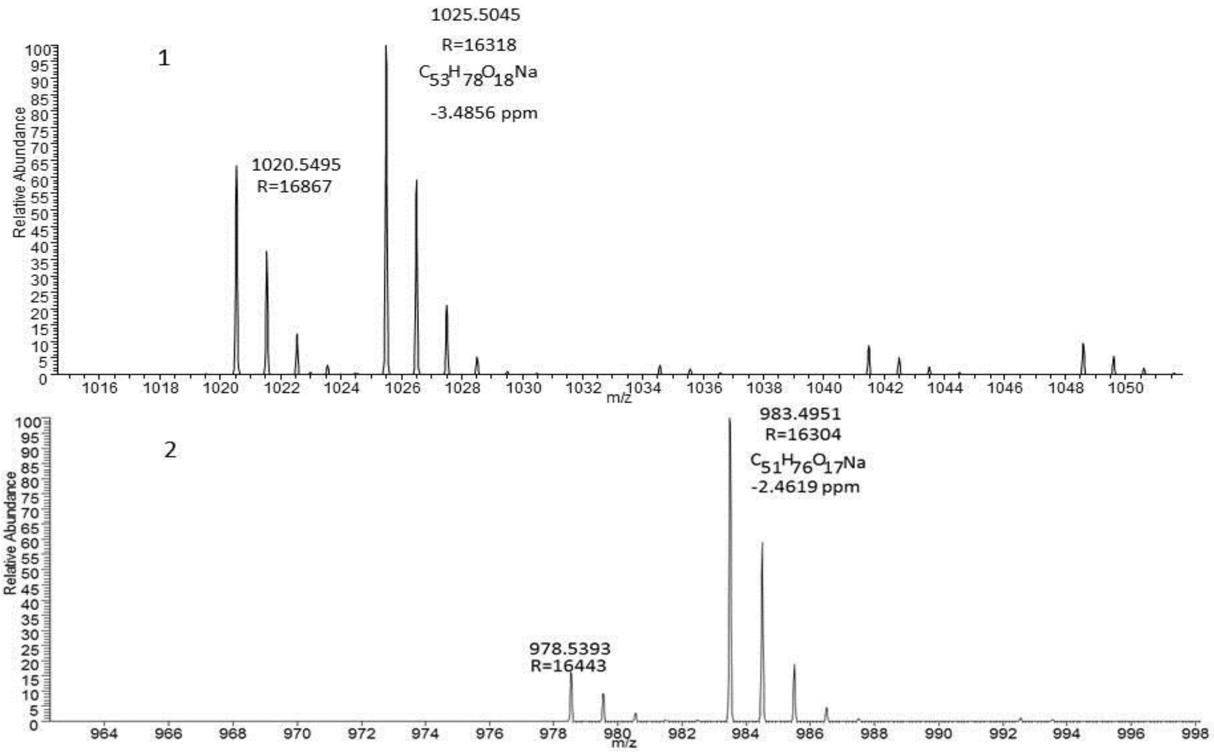

(**1**) Esa-*O*-acetylated α-hederin: experimental value [M + Na]$^+$ *m*/*z* 1025.5045 (theoretical [M + Na]$^+$ *m*/*z* 1025.5080). Presence of ammonium adduct at *m*/*z* 1020.5495 (**2**). Penta-*O*-acetylated α-hederin: experimental value [M + Na]$^+$ *m*/*z* 983.4951 (theoretical [M + Na]$^+$ *m*/*z* 983.4974). Presence of ammonium adduct at *m*/z 978.5393.

Sample 17a

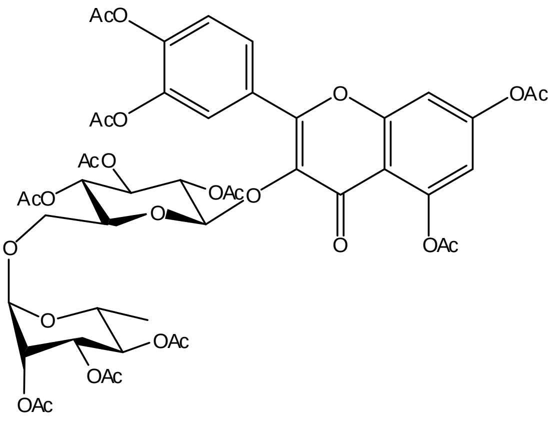

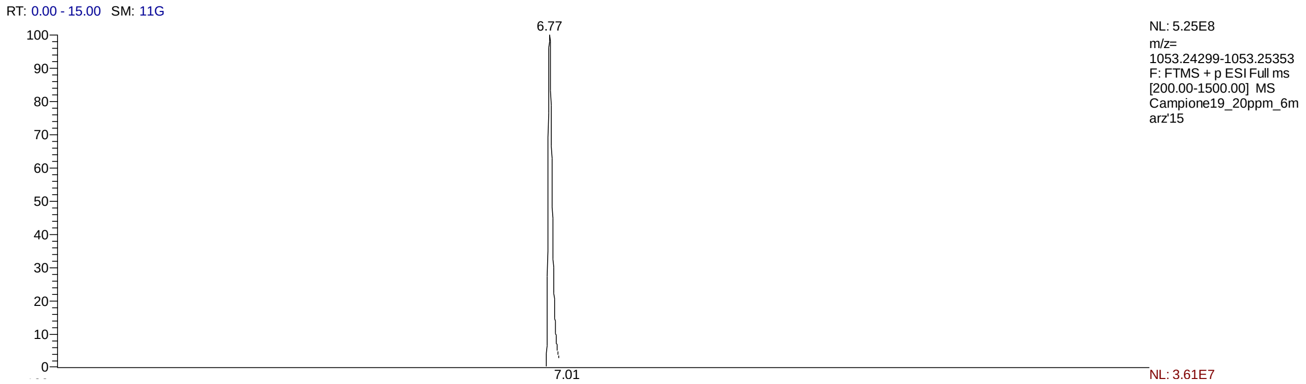

LCHRMS (*m*/*z*, [M + Na]$^+$) of spectrum of *O*-acetylated rutin (isolated product) in extract ion (XIC) of Peracetylated-rutin.

HRMS (m/z, $[M + Na]^+$; R = operative resolution; accuracy (ppm)) spectra of peracetylated rutin:

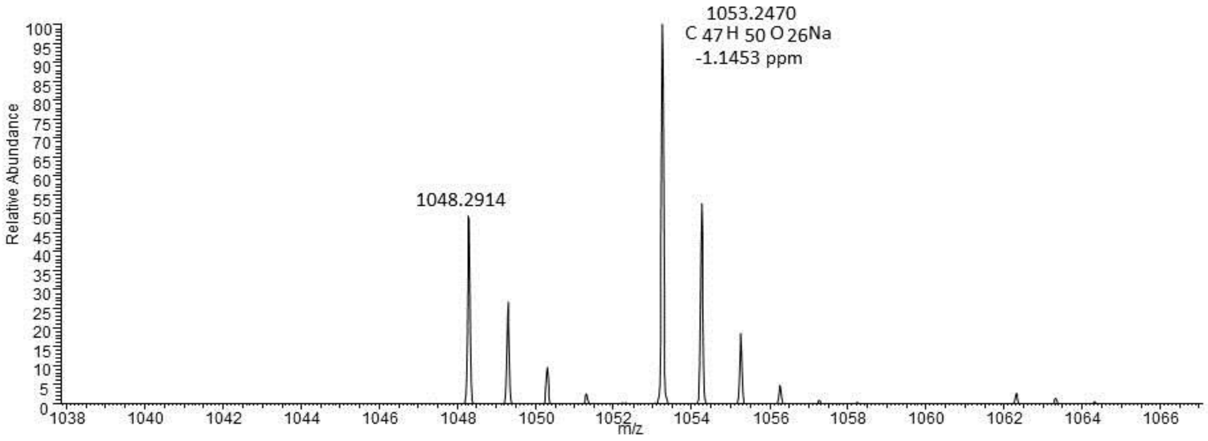

Peracetylated-rutin: experimental value $[M + Na]^+$ *m/z* 1053.2470 (theoretical $[M + Na]^+$ *m/z* 1053.2482). Presence of ammonium adduct at *m/z* 1048.2914.

^{1}H NMR spectra

Sample **3a**

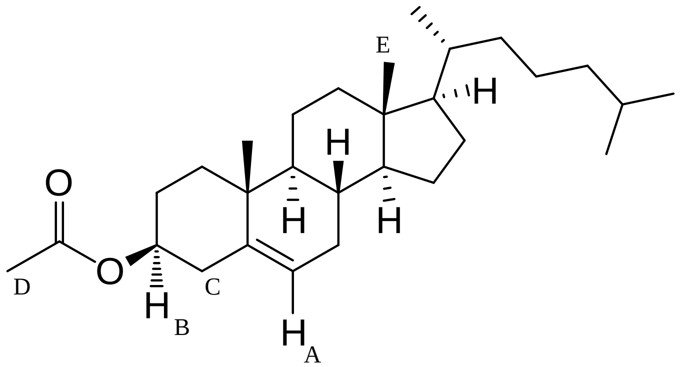

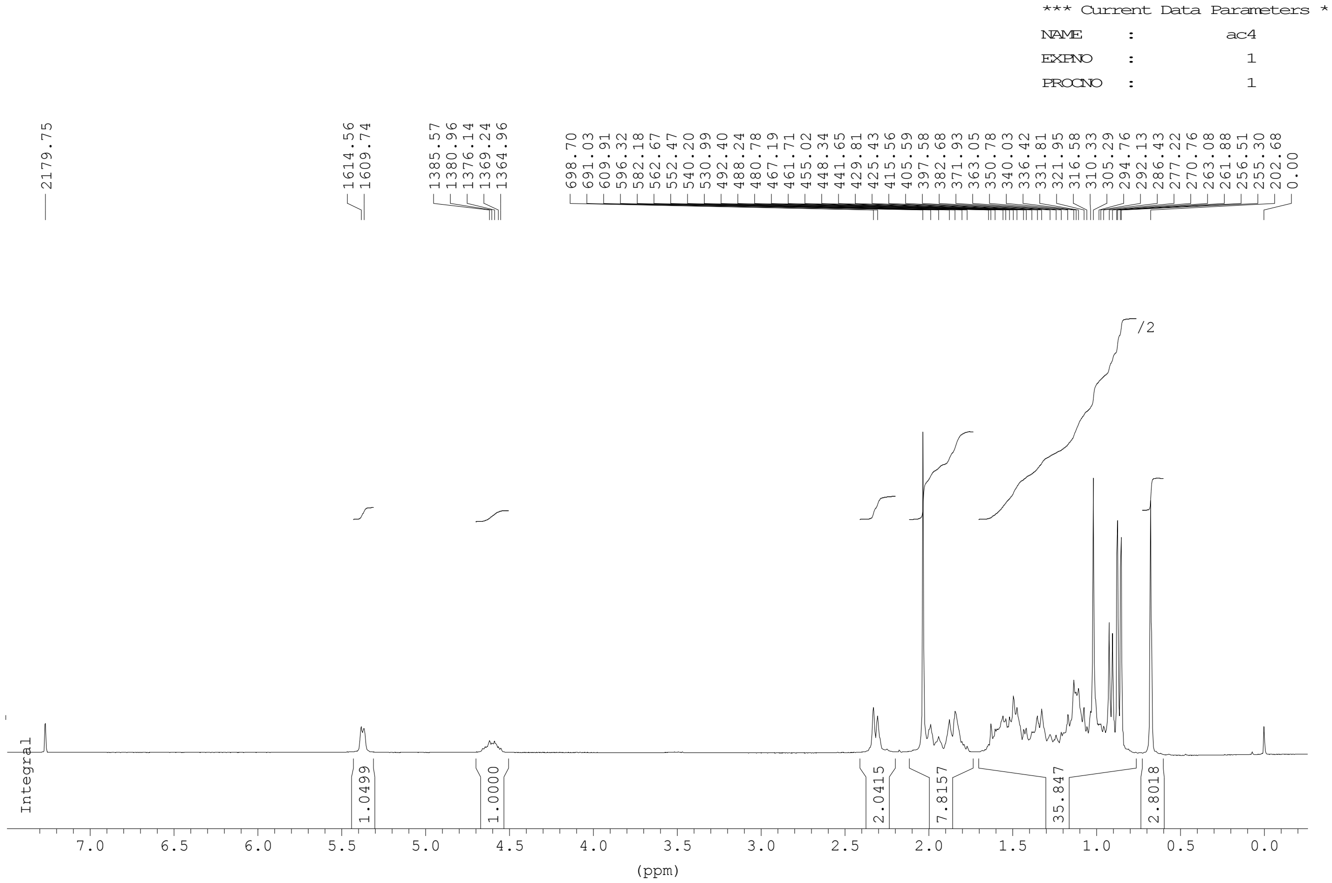

Sample **5a**

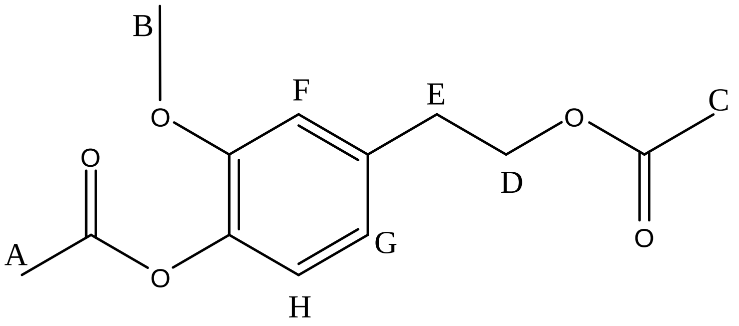

A
B
C
D
E
F
G
H
O

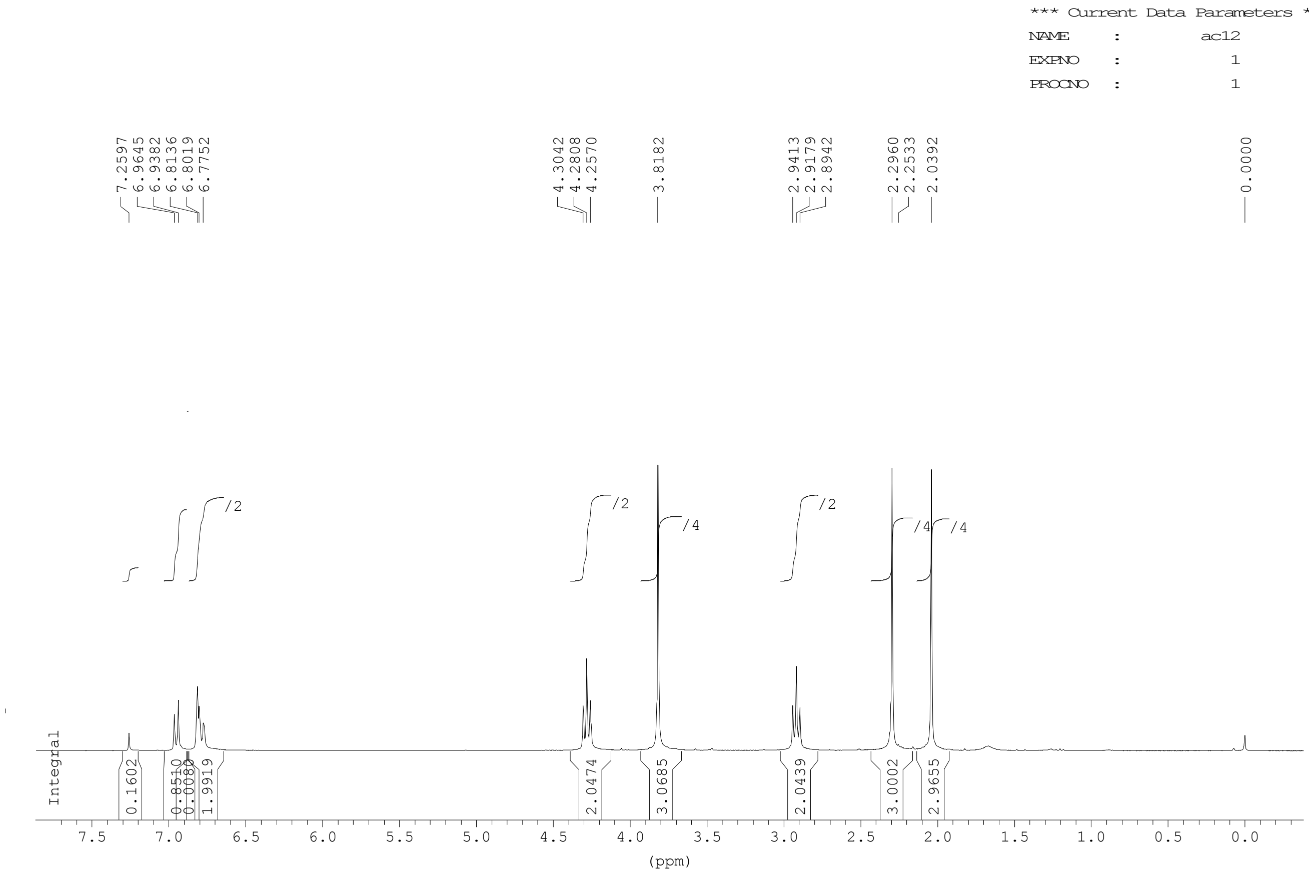

*** Current Data Parameters *
NAME : ac12
EXPNO : 1
PROCNO : 1
7.2597
6.9645
6.9382
6.8136
6.8019
6.7752
4.3042
4.2808
4.2570
3.8182
2.9413
2.9179
2.8942
2.2960
2.2533
2.0392
0.0000
Integral
0.1602
0.8510
0.0080
1.9919
2.0474
3.0685
2.0439
3.0002
2.9655
(ppm)

Sample **6a**

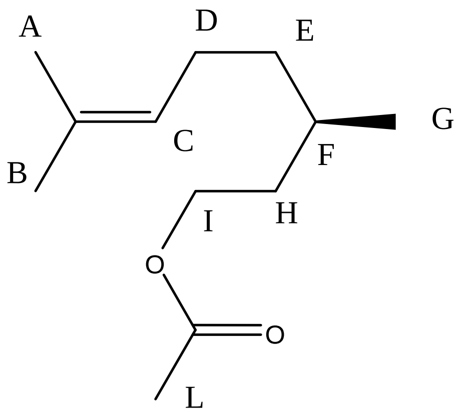
A
B
C
D
E
F
G
H
I
O
O
L

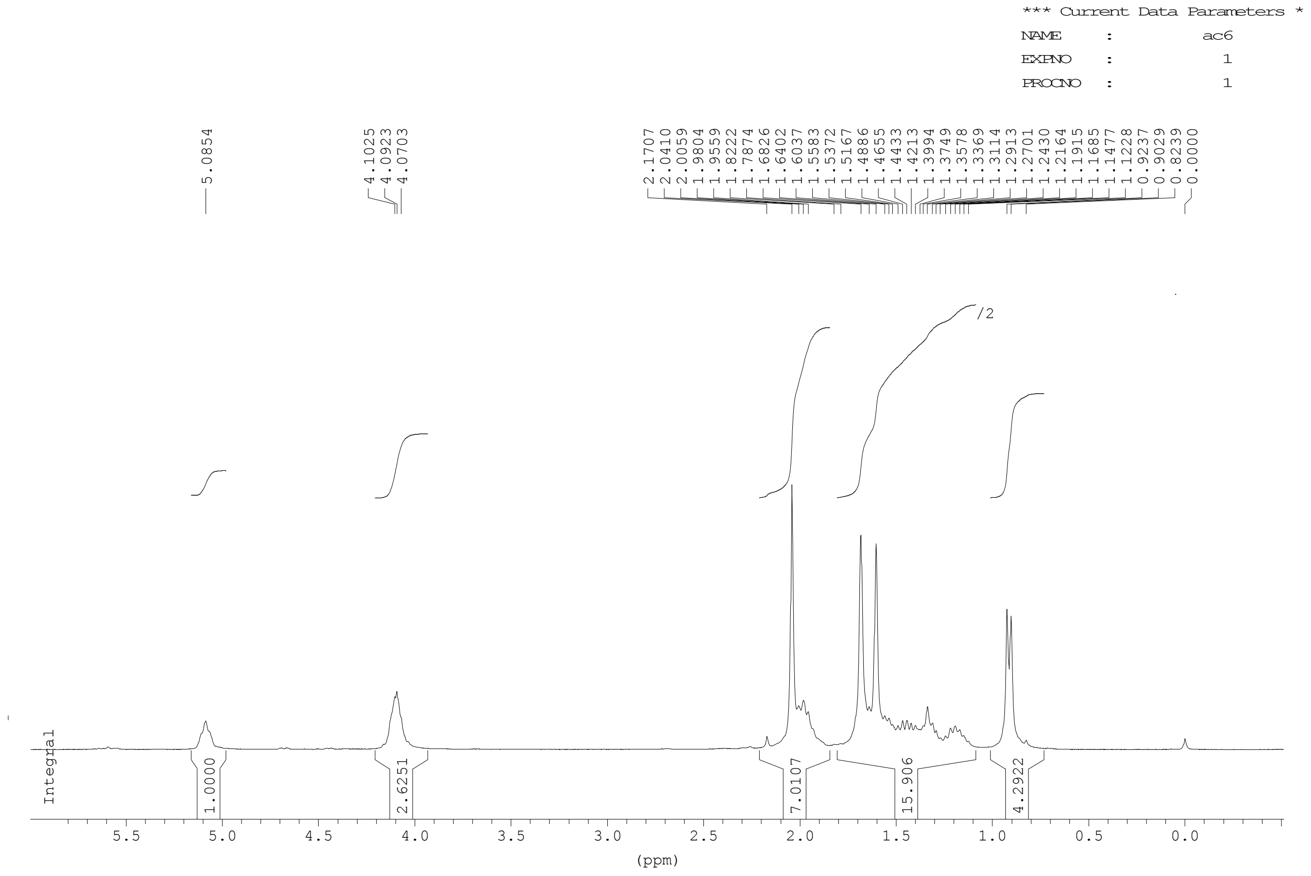
*** Current Data Parameters *
NAME : ac6
EXPNO : 1
PROCNO : 1
5.0854
4.1025
4.0923
4.0703
2.1707
2.0410
2.0059
1.9804
1.9559
1.8222
1.7874
1.6826
1.6402
1.6037
1.5583
1.5372
1.5167
1.4886
1.4655
1.4433
1.4213
1.3994
1.3749
1.3578
1.3369
1.3114
1.2913
1.2701
1.2430
1.2164
1.1915
1.1685
1.1477
1.1228
0.9237
0.9029
0.8239
0.0000
/2
Integral
1.0000
2.6251
7.0107
15.906
4.2922
5.5
5.0
4.5
4.0
3.5
3.0
2.5
2.0
1.5
1.0
0.5
0.0
(ppm)

Sample **7a**

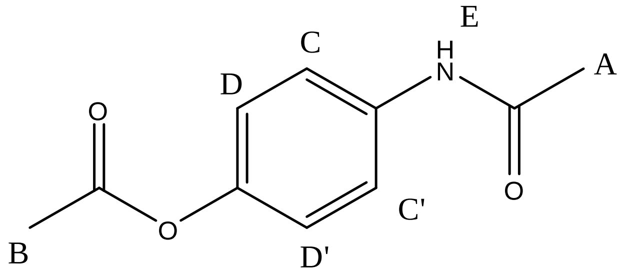
E
H
N
A
C
D
O
O
C'
O
B
D'

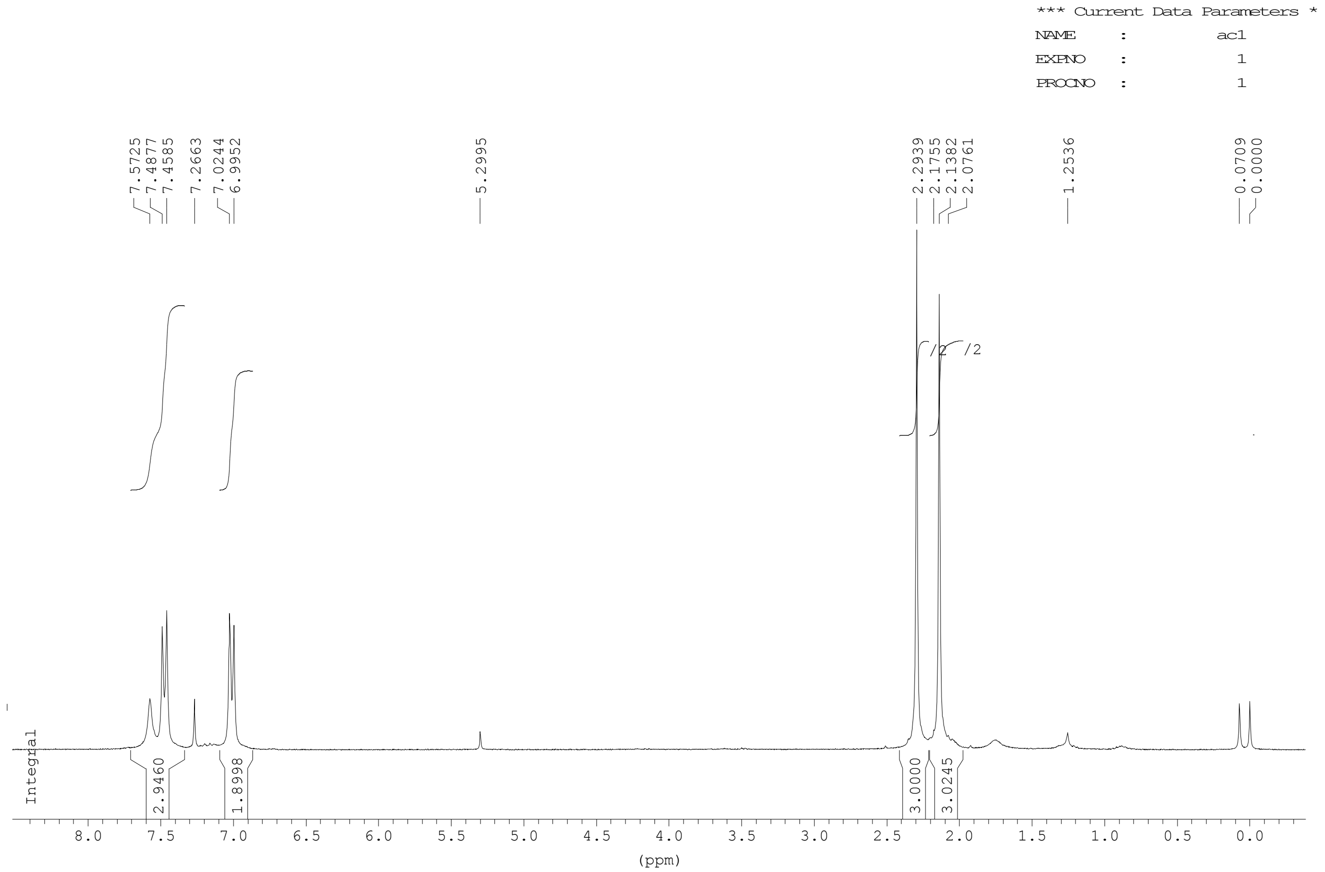
*** Current Data Parameters *
NAME : ac1
EXPNO : 1
PROCNO : 1
7.5725
7.4877
7.4585
7.2663
7.0244
6.9952
5.2995
2.2939
2.1755
2.1382
2.0761
1.2536
0.0709
0.0000
Integral
2.9460
1.8998
3.0000
3.0245
8.0 7.5 7.0 6.5 6.0 5.5 5.0 4.5 4.0 3.5 3.0 2.5 2.0 1.5 1.0 0.5 0.0
(ppm)

Sample **8a**

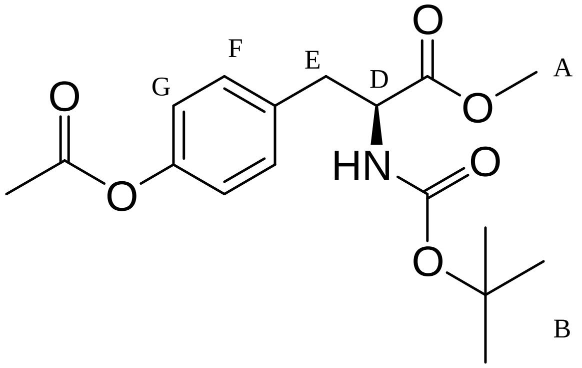
A
B
C
D
E
F
G
HN
O

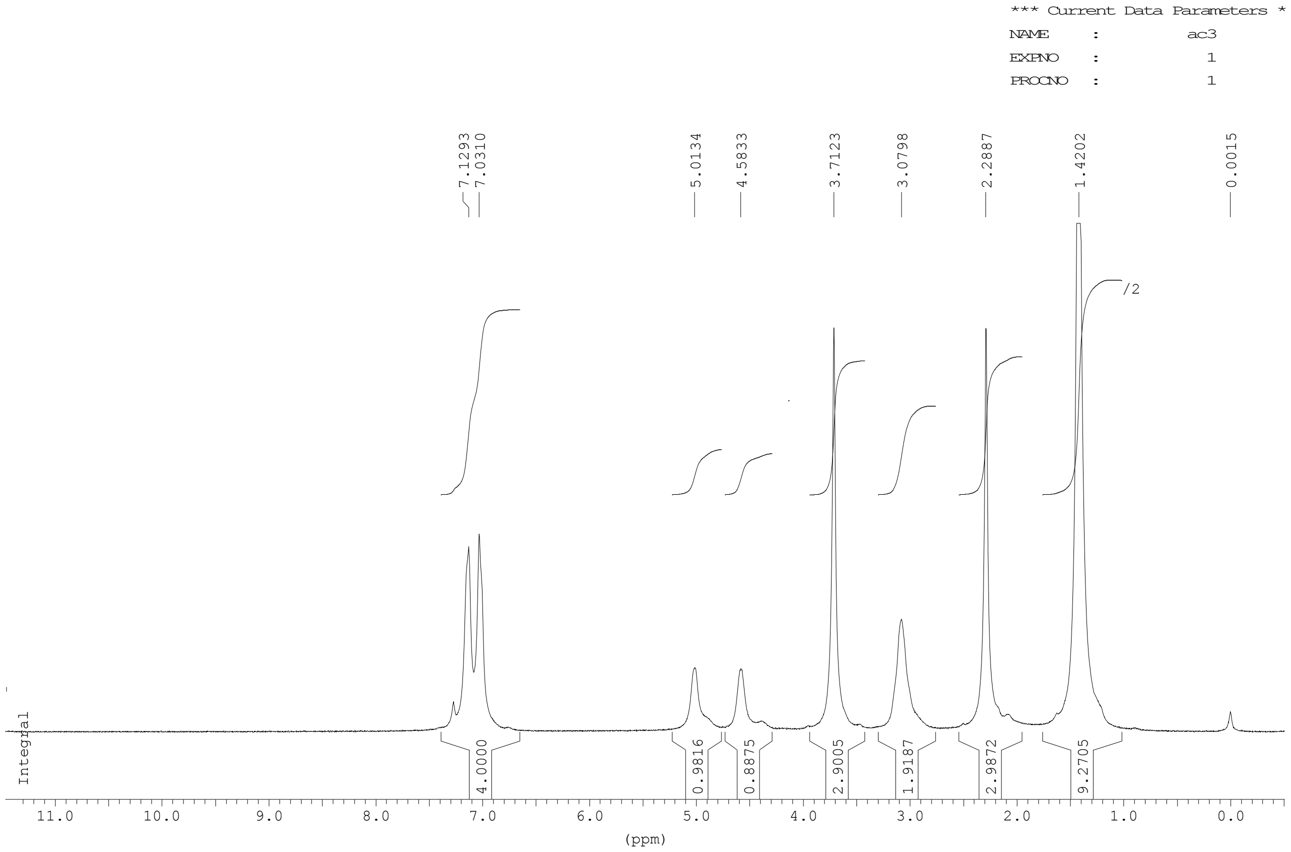
*** Current Data Parameters *
NAME : ac3
EXPNO : 1
PROCNO : 1
7.1293
7.0310
5.0134
4.5833
3.7123
3.0798
2.2887
1.4202
0.0015
/2
Integral
4.0000
0.9816
0.8875
2.9005
1.9187
2.9872
9.2705
11.0
10.0
9.0
8.0
7.0
6.0
5.0
4.0
3.0
2.0
1.0
0.0
(ppm)

Sample **9a**

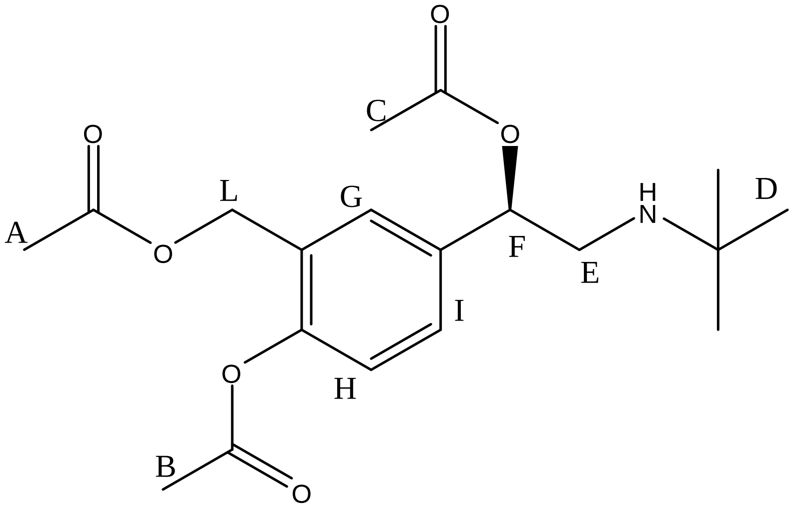
A
B
C
D
E
F
G
H
I
L

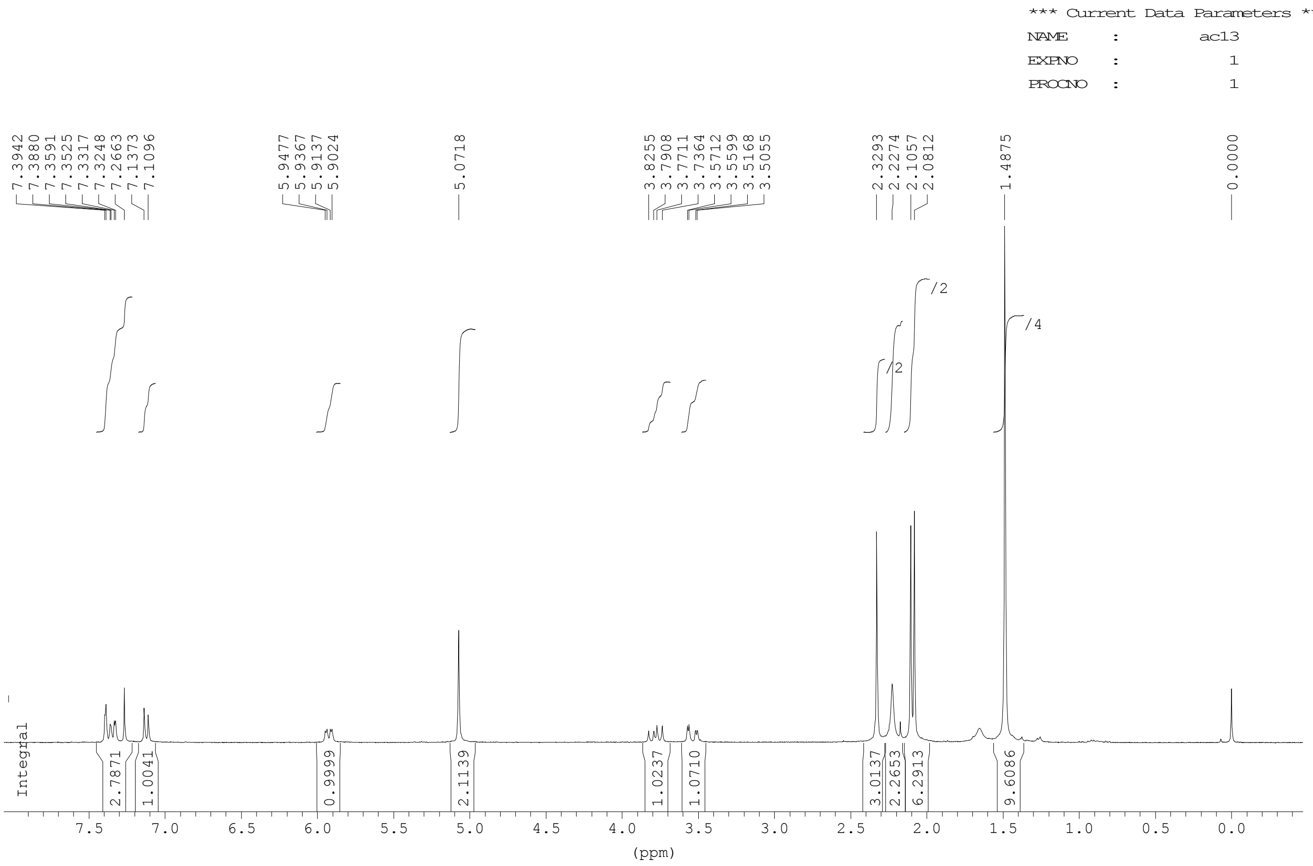
*** Current Data Parameters **
NAME : ac13
EXPNO : 1
PROCNO : 1
7.3942
7.3880
7.3591
7.3525
7.3317
7.3248
7.2663
7.1373
7.1096
5.9477
5.9367
5.9137
5.9024
5.0718
3.8255
3.7908
3.7711
3.7364
3.5712
3.5599
3.5168
3.5055
2.3293
2.2274
2.1057
2.0812
1.4875
0.0000
/2
/2
/4
Integral
2.7871
1.0041
0.9999
2.1139
1.0237
1.0710
3.0137
2.2653
6.2913
9.6086
(ppm)

Sample **10a**

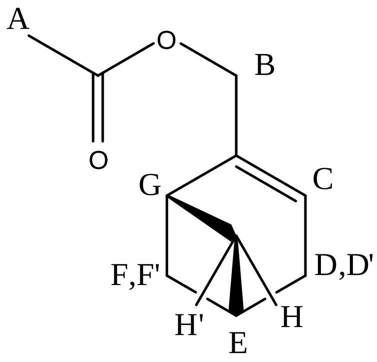
A
O
B
O
G
C
F,F'
D,D'
H'
H
E

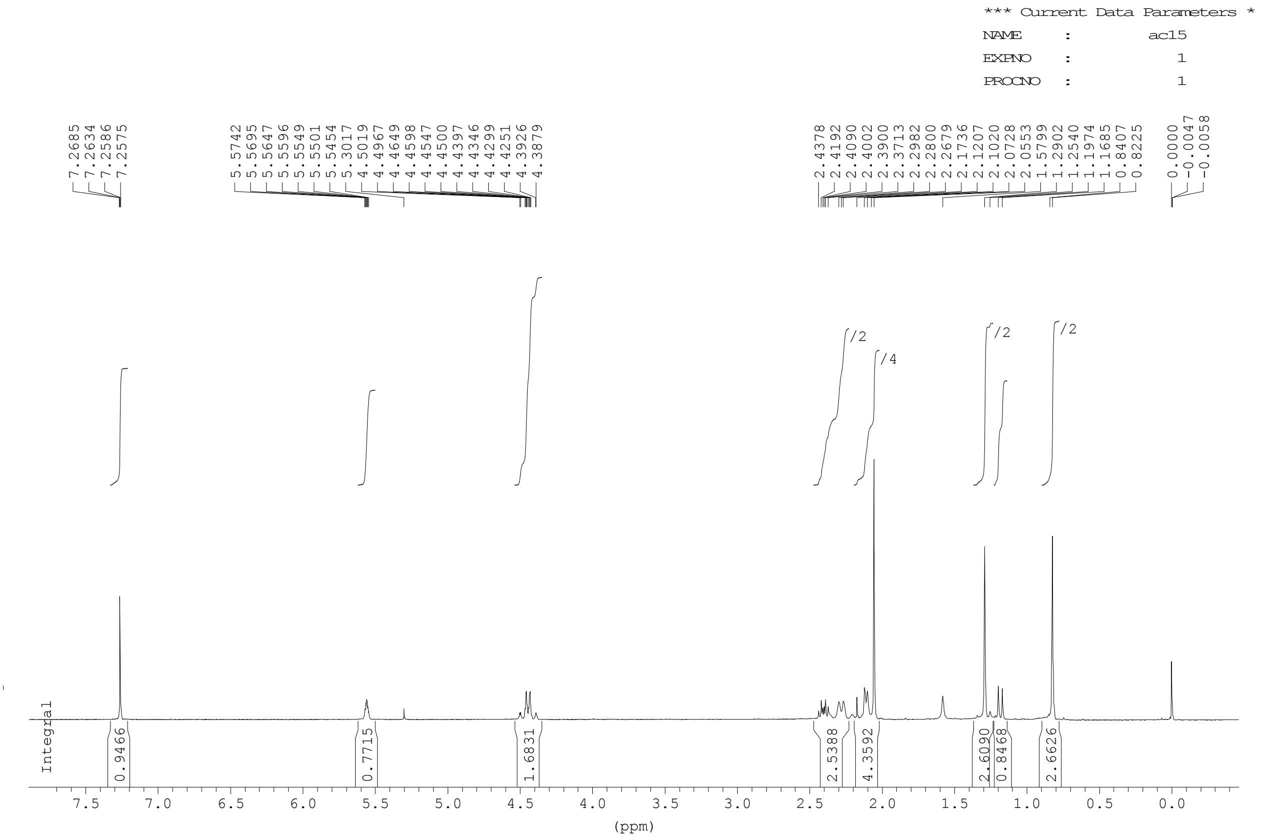
*** Current Data Parameters *
NAME : ac15
EXPNO : 1
PROCNO : 1
7.2685 7.2634 7.2586 7.2575
5.5742 5.5695 5.5647 5.5596 5.5549 5.5501 5.5454 5.3017 4.5019 4.4967 4.4649 4.4598 4.4547 4.4500 4.4397 4.4346 4.4299 4.4251 4.3926 4.3879
2.4378 2.4192 2.4090 2.4002 2.3900 2.3713 2.2982 2.2800 2.2679 2.1736 2.1207 2.1020 2.0728 2.0553 1.5799 1.2902 1.2540 1.1974 1.1685 0.8407 0.8225
0.0000 -0.0047 -0.0058
/2
/4
/2
/2
Integral
0.9466
0.7715
1.6831
2.5388
4.3592
2.6090
0.8468
2.6626
7.5 7.0 6.5 6.0 5.5 5.0 4.5 4.0 3.5 3.0 2.5 2.0 1.5 1.0 0.5 0.0
(ppm)

Sample **12°**

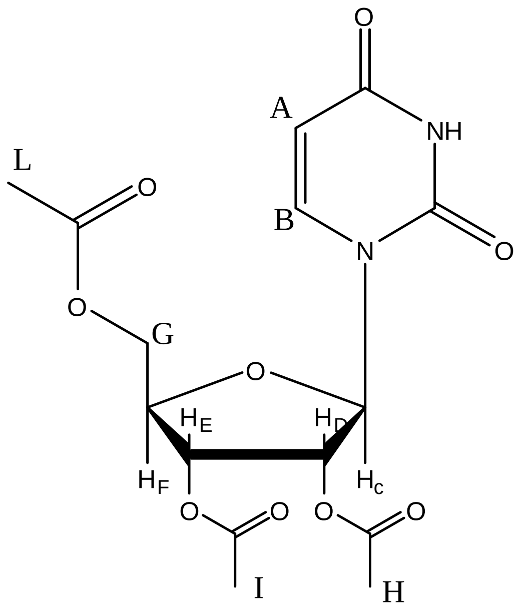

O
A
NH
L
O
B
O
N
O
G
O
HE
HD
HF
Hc
O
O
O
O
I
H

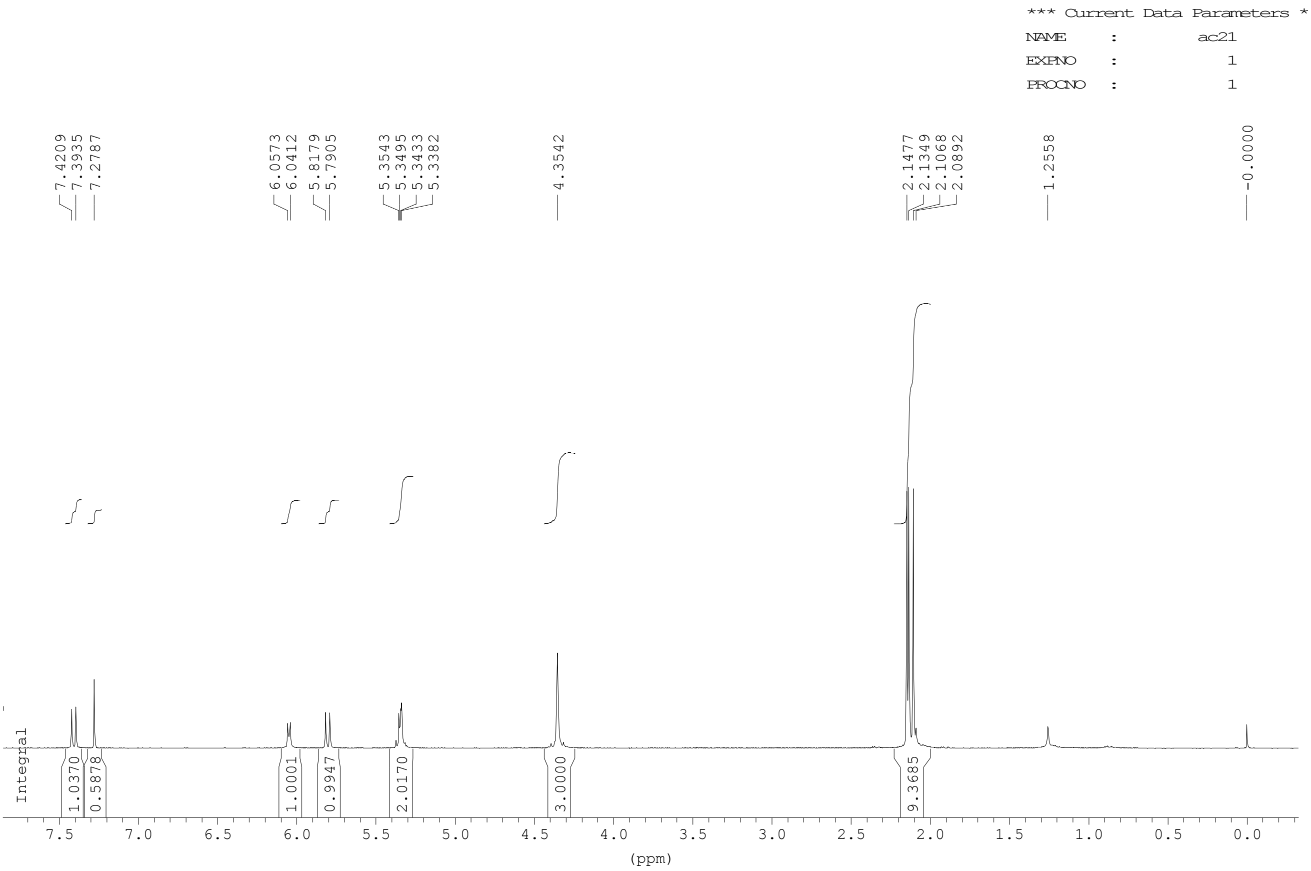

*** Current Data Parameters *
NAME : ac21
EXPNO : 1
PROCNO : 1
7.4209
7.3935
7.2787
6.0573
6.0412
5.8179
5.7905
5.3543
5.3495
5.3433
5.3382
4.3542
2.1477
2.1349
2.1068
2.0892
1.2558
-0.0000
Integral
1.0370
0.5878
1.0001
0.9947
2.0170
3.0000
9.3685
7.5
7.0
6.5
6.0
5.5
5.0
4.5
4.0
3.5
3.0
2.5
2.0
1.5
1.0
0.5
0.0
(ppm)

Sample **13a**

(mix, characterization of the major product)

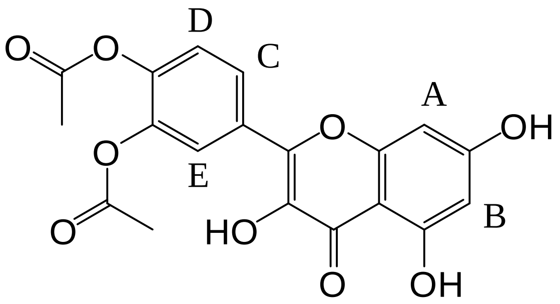

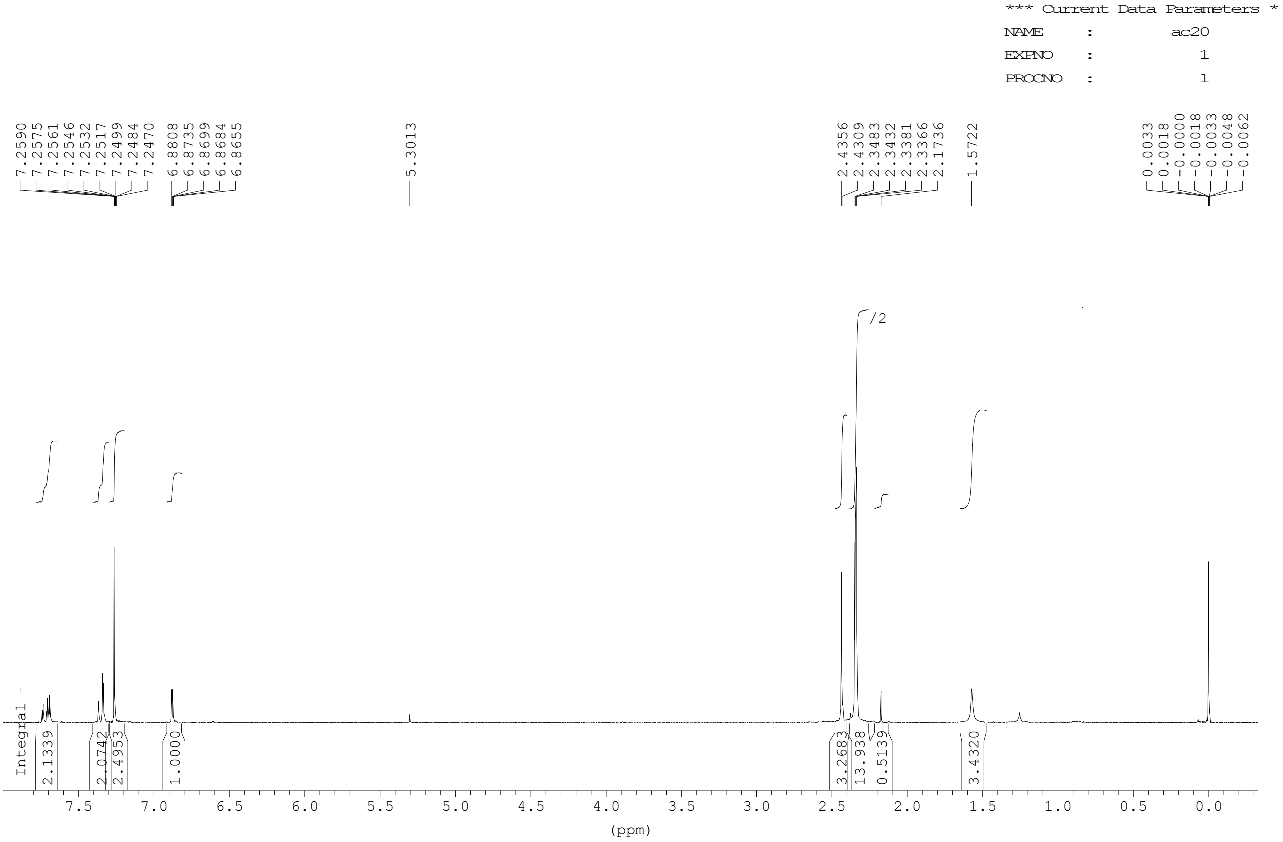

Sample **16a**

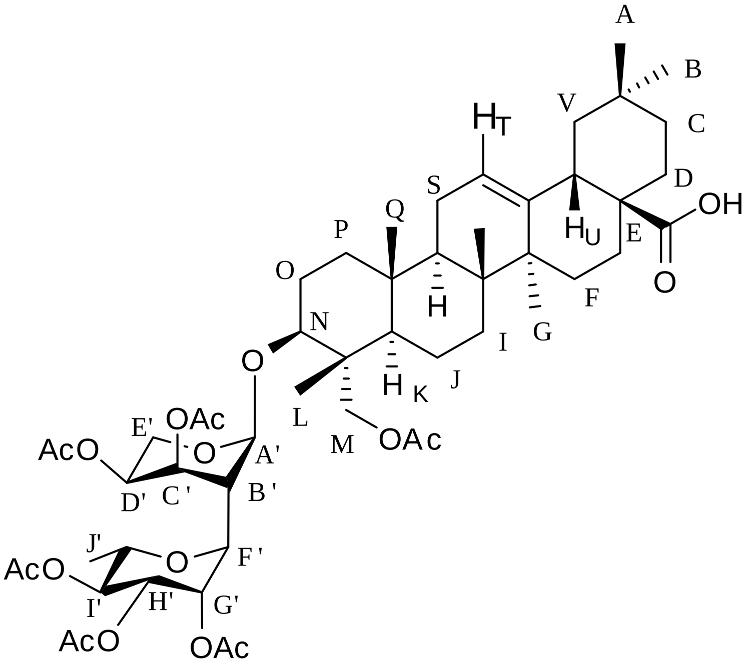
A
B
V
C
H_T
D
S
OH
Q
H_U
E
P
O
O
F
H
N
I
G
O
J
H K
L
E'
OAc
M
OAc
AcO
O
A'
D'
C'
B'
J'
AcO
O
F'
I'
H'
G'
AcO
OAc

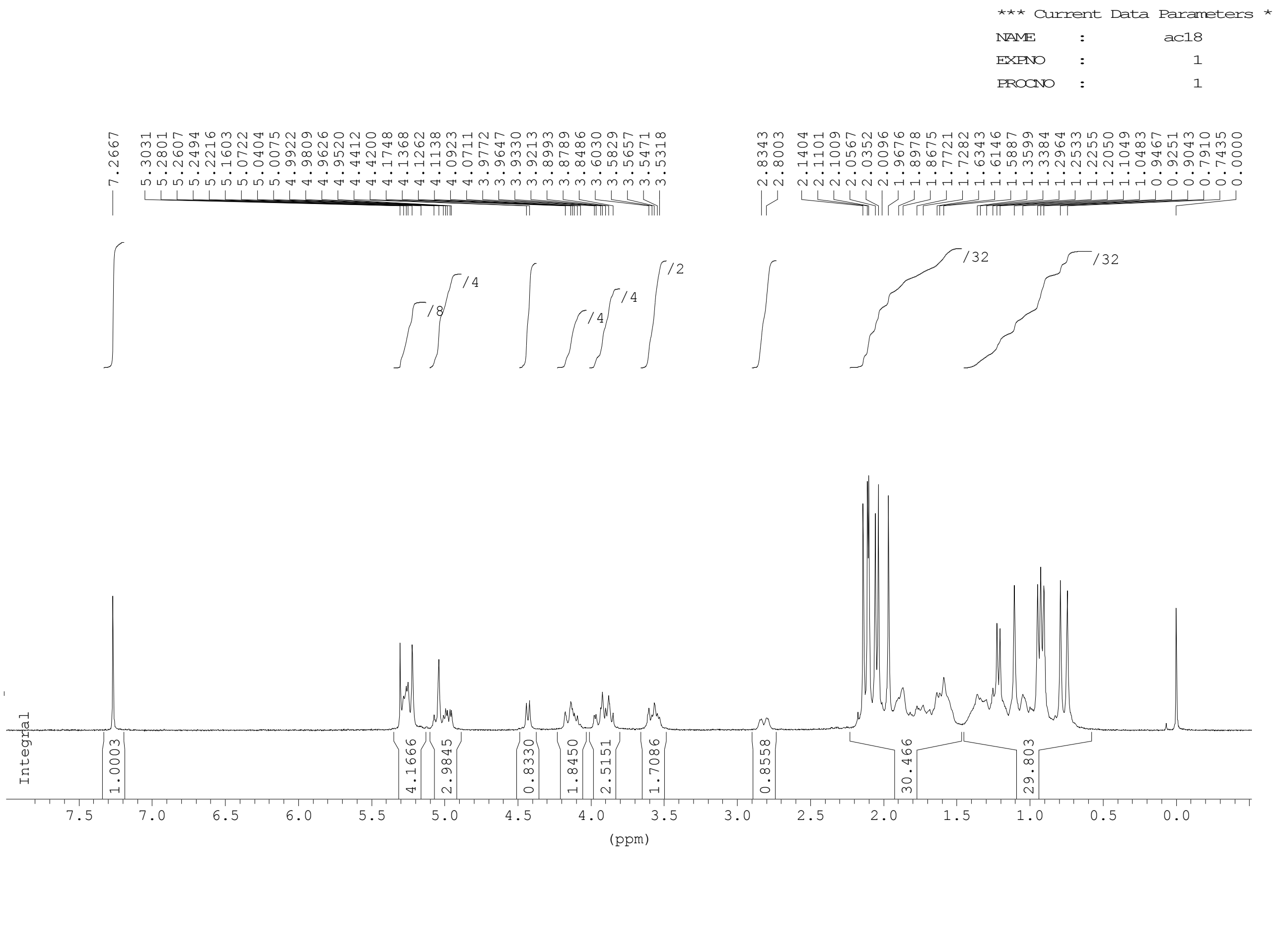
*** Current Data Parameters *
NAME : ac18
EXPNO : 1
PROCNO : 1
7.2667
5.3031
5.2801
5.2607
5.2494
5.2216
5.1603
5.0722
5.0404
5.0075
4.9922
4.9809
4.9626
4.9520
4.4412
4.4200
4.1748
4.1368
4.1262
4.1138
4.0923
4.0711
3.9772
3.9647
3.9330
3.9213
3.8993
3.8789
3.8486
3.6030
3.5829
3.5657
3.5471
3.5318
2.8343
2.8003
2.1404
2.1101
2.1009
2.0567
2.0352
2.0096
1.9676
1.8978
1.8675
1.7721
1.7282
1.6343
1.6146
1.5887
1.3599
1.3384
1.2964
1.2533
1.2255
1.2050
1.1049
1.0483
0.9467
0.9251
0.9043
0.7910
0.7435
0.0000
/8
/4
/4
/4
/2
/32
/32
Integral
1.0003
4.1666
2.9845
0.8330
1.8450
2.5151
1.7086
0.8558
30.466
29.803
7.5
7.0
6.5
6.0
5.5
5.0
4.5
4.0
3.5
3.0
2.5
2.0
1.5
1.0
0.5
0.0
(ppm)

Sample **17a**

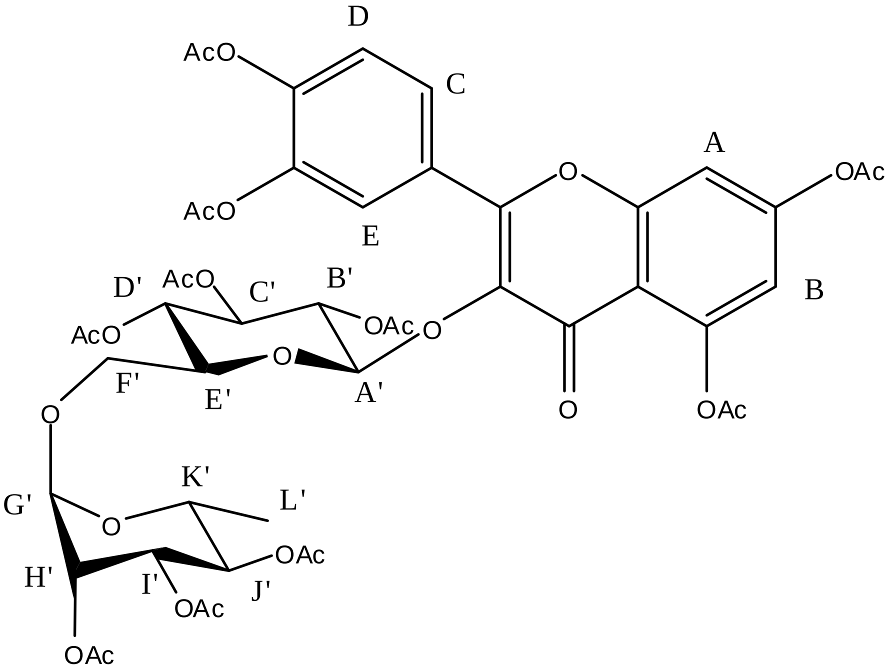

AcO
D
C
AcO
E
A
O
OAc
B
D'
AcO
C'
B'
AcO
OAc
O
O
F'
E'
A'
O
OAc
O
K'
G'
L'
O
OAc
H'
I'
J'
OAc
OAc

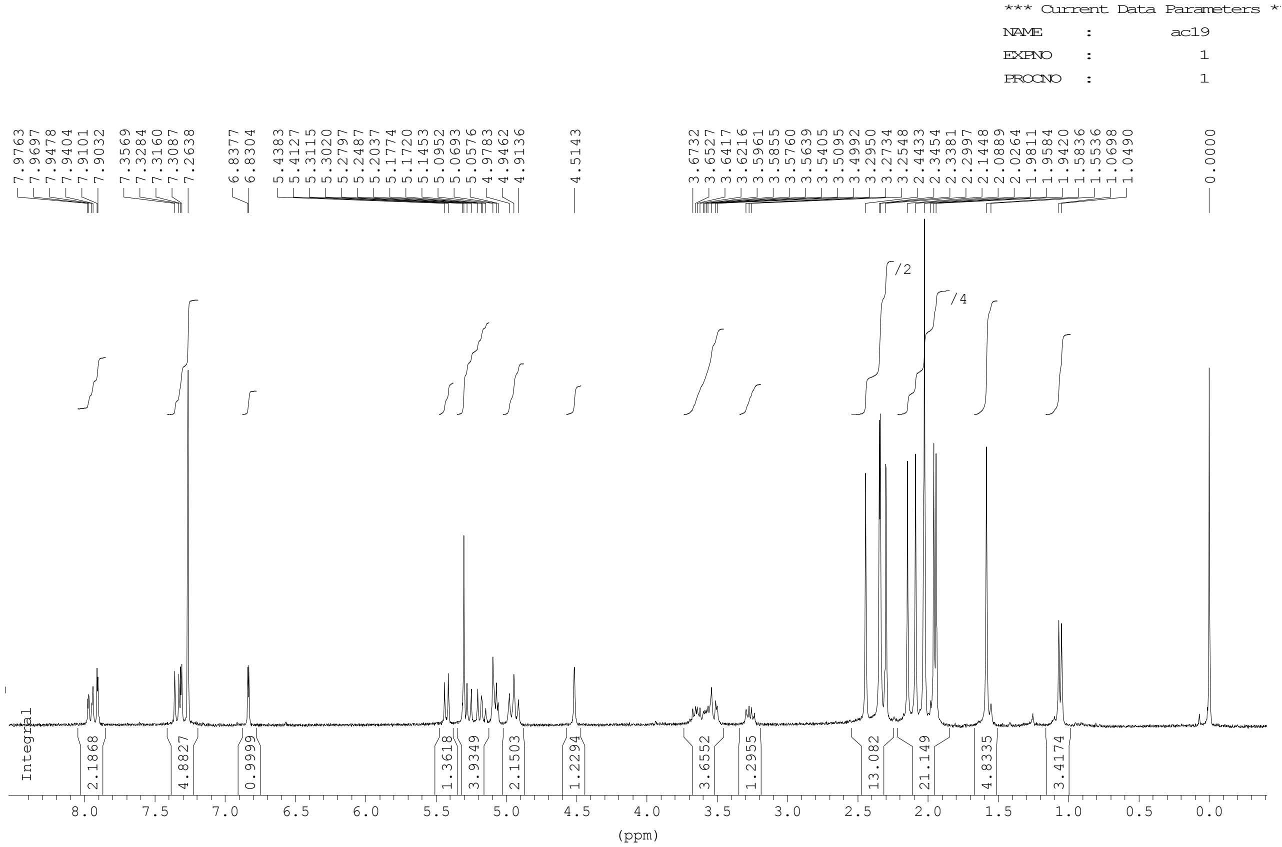

*** Current Data Parameters *
NAME : ac19
EXPNO : 1
PROCNO : 1
7.9763
7.9697
7.9478
7.9404
7.9101
7.9032
7.3569
7.3284
7.3160
7.3087
7.2638
6.8377
6.8304
5.4383
5.4127
5.3115
5.3020
5.2797
5.2487
5.2037
5.1774
5.1720
5.1453
5.0952
5.0693
5.0576
4.9783
4.9462
4.9136
4.5143
3.6732
3.6527
3.6417
3.6216
3.5961
3.5855
3.5760
3.5639
3.5405
3.5095
3.4992
3.2950
3.2734
3.2548
2.4433
2.3454
2.3381
2.2997
2.1448
2.0889
2.0264
1.9811
1.9584
1.9420
1.5836
1.5536
1.0698
1.0490
0.0000
/2
/4
Integral
2.1868
4.8827
0.9999
1.3618
3.9349
2.1503
1.2294
3.6552
1.2955
13.082
21.149
4.8335
3.4174
8.0
7.5
7.0
6.5
6.0
5.5
5.0
4.5
4.0
3.5
3.0
2.5
2.0
1.5
1.0
0.5
0.0
(ppm)

^{13}C NMR spectra

Sample **3a**

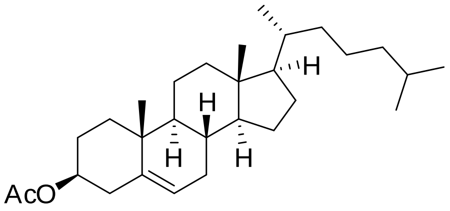

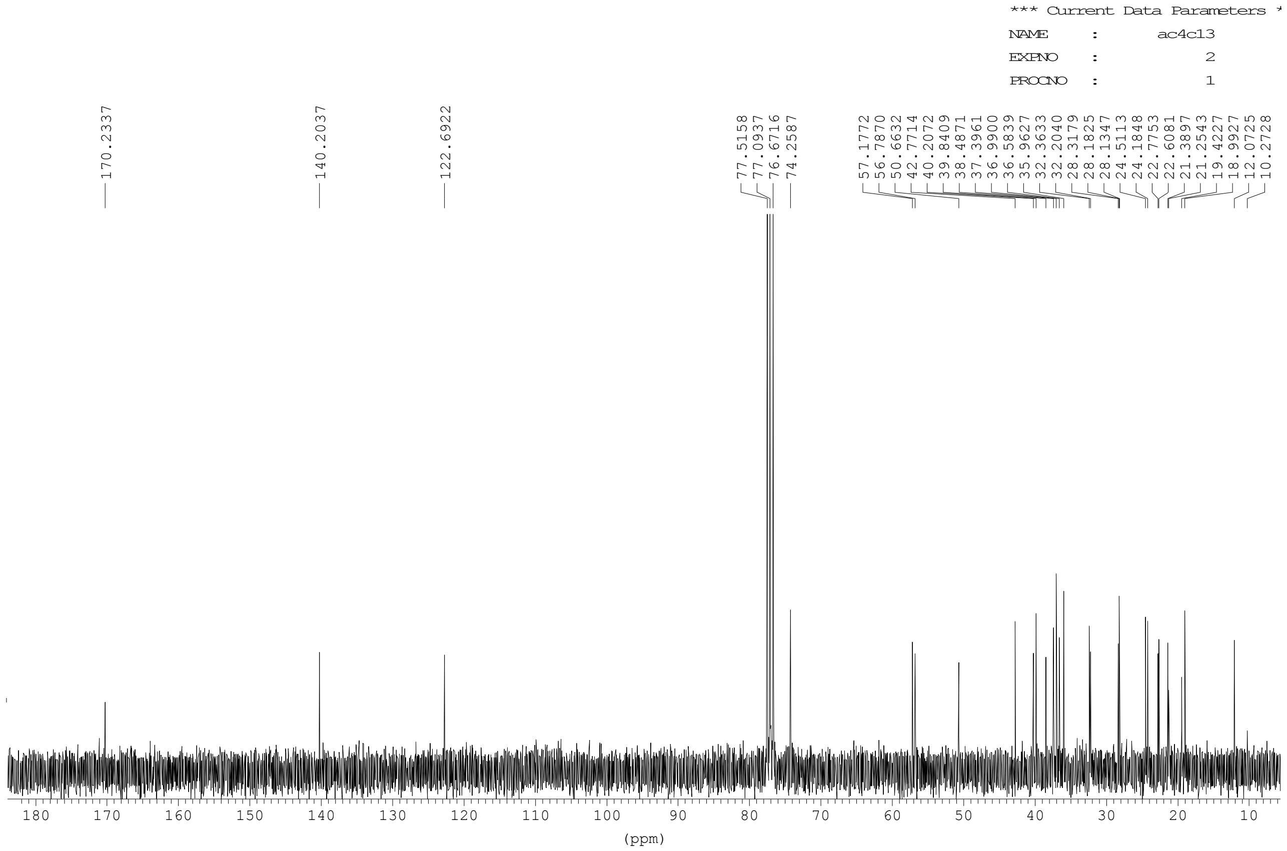

Sample **5a**

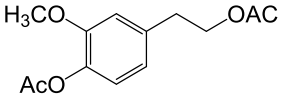
H3CO
OAC
AcO

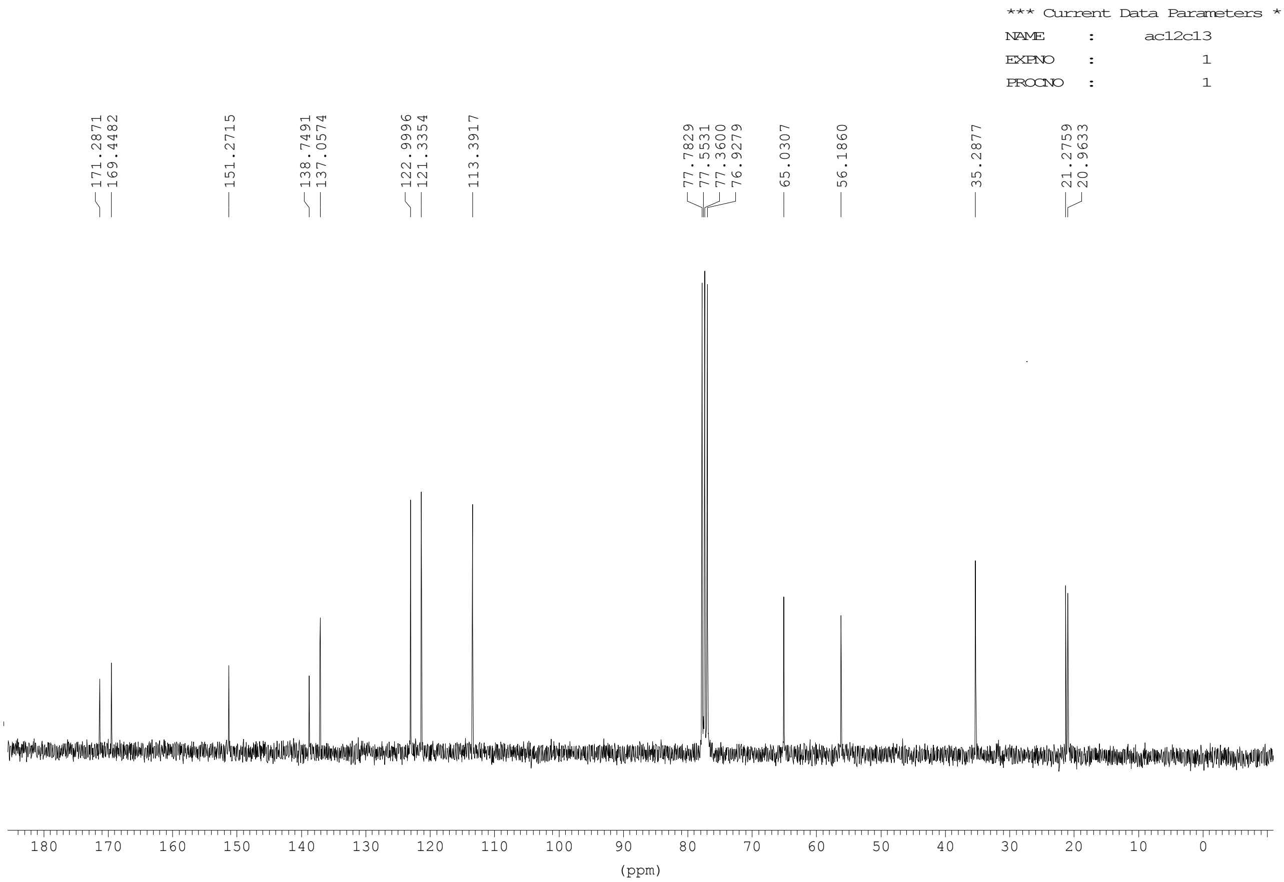
*** Current Data Parameters *
NAME : ac12c13
EXPNO : 1
PROCNO : 1
171.2871
169.4482
151.2715
138.7491
137.0574
122.9996
121.3354
113.3917
77.7829
77.5531
77.3600
76.9279
65.0307
56.1860
35.2877
21.2759
20.9633
180 170 160 150 140 130 120 110 100 90 80 70 60 50 40 30 20 10 0
(ppm)

Sample **6a**

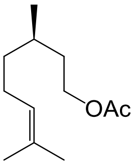
OAc

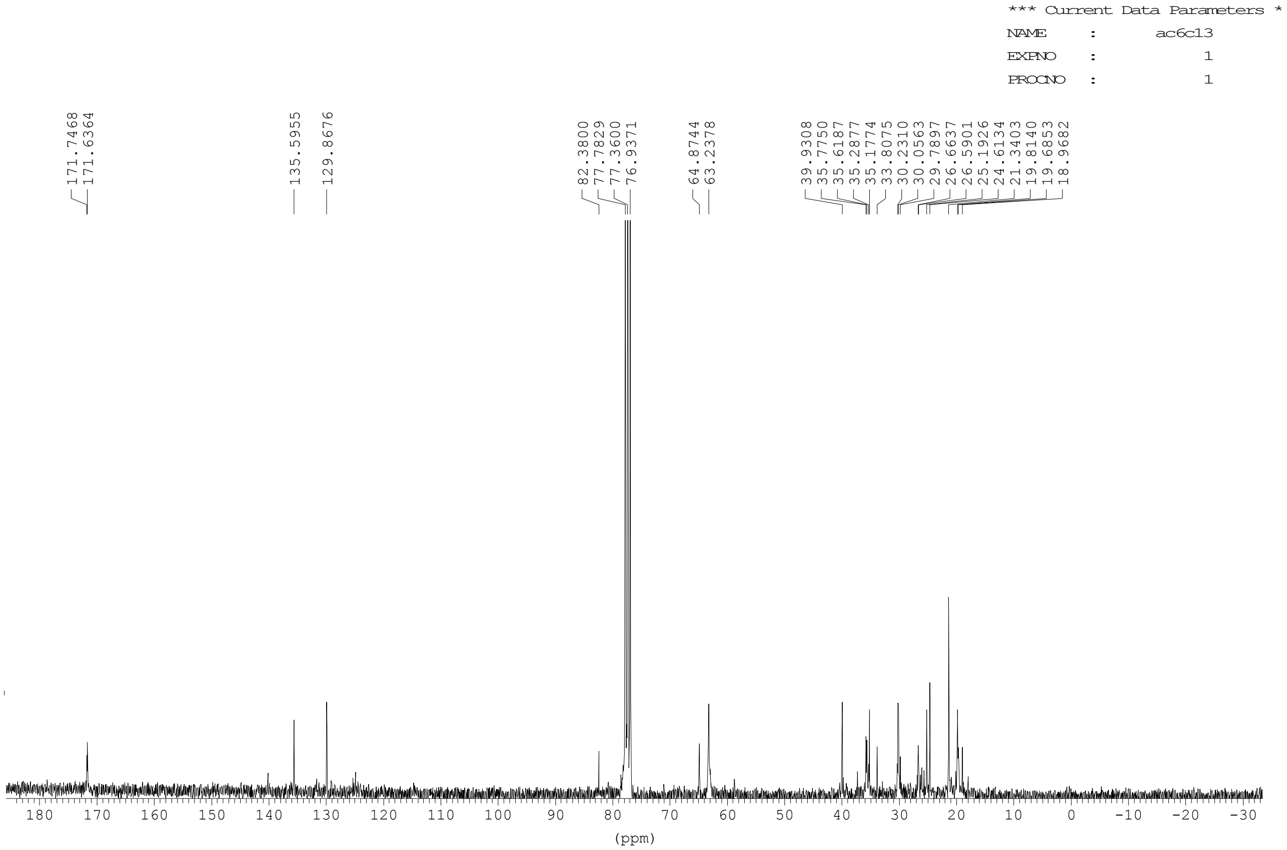
*** Current Data Parameters *
NAME : ac6c13
EXPNO : 1
PROCNO : 1
171.7468
171.6364
135.5955
129.8676
82.3800
77.7829
77.3600
76.9371
64.8744
63.2378
39.9308
35.7750
35.6187
35.2877
35.1774
33.8075
30.2310
30.0563
29.7897
26.6637
26.5901
25.1926
24.6134
21.3403
19.8140
19.6853
18.9682
180
170
160
150
140
130
120
110
100
90
80
70
60
50
40
30
20
10
0
-10
-20
-30
(ppm)

Sample **7a**

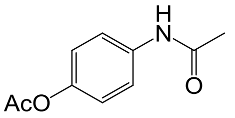
H
N
O
AcO

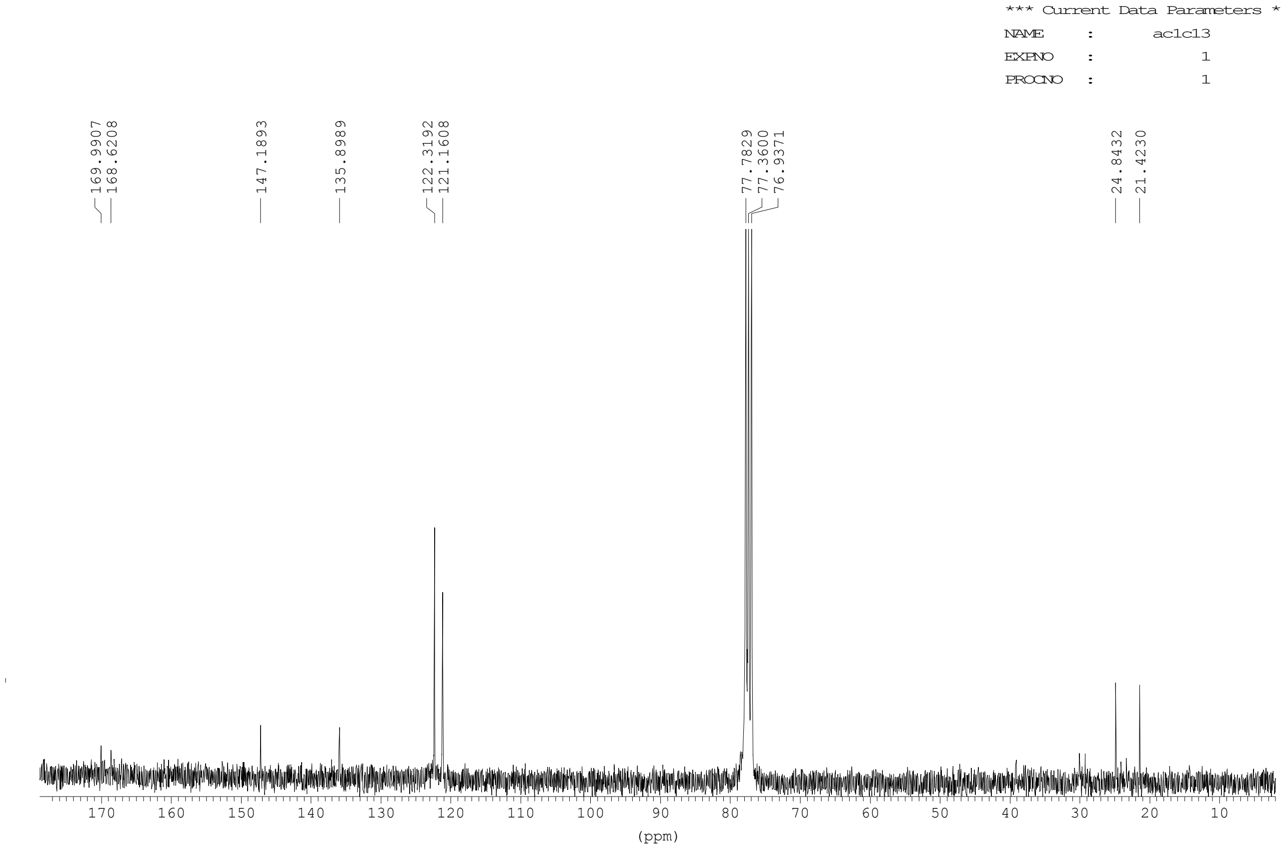
*** Current Data Parameters *
NAME : ac1c13
EXPNO : 1
PROCNO : 1
169.9907
168.6208
147.1893
135.8989
122.3192
121.1608
77.7829
77.3600
76.9371
24.8432
21.4230
170 160 150 140 130 120 110 100 90 80 70 60 50 40 30 20 10
(ppm)

Sample **8a**

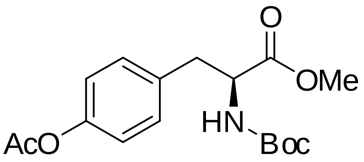
O
OMe
AcO
HN
Boc

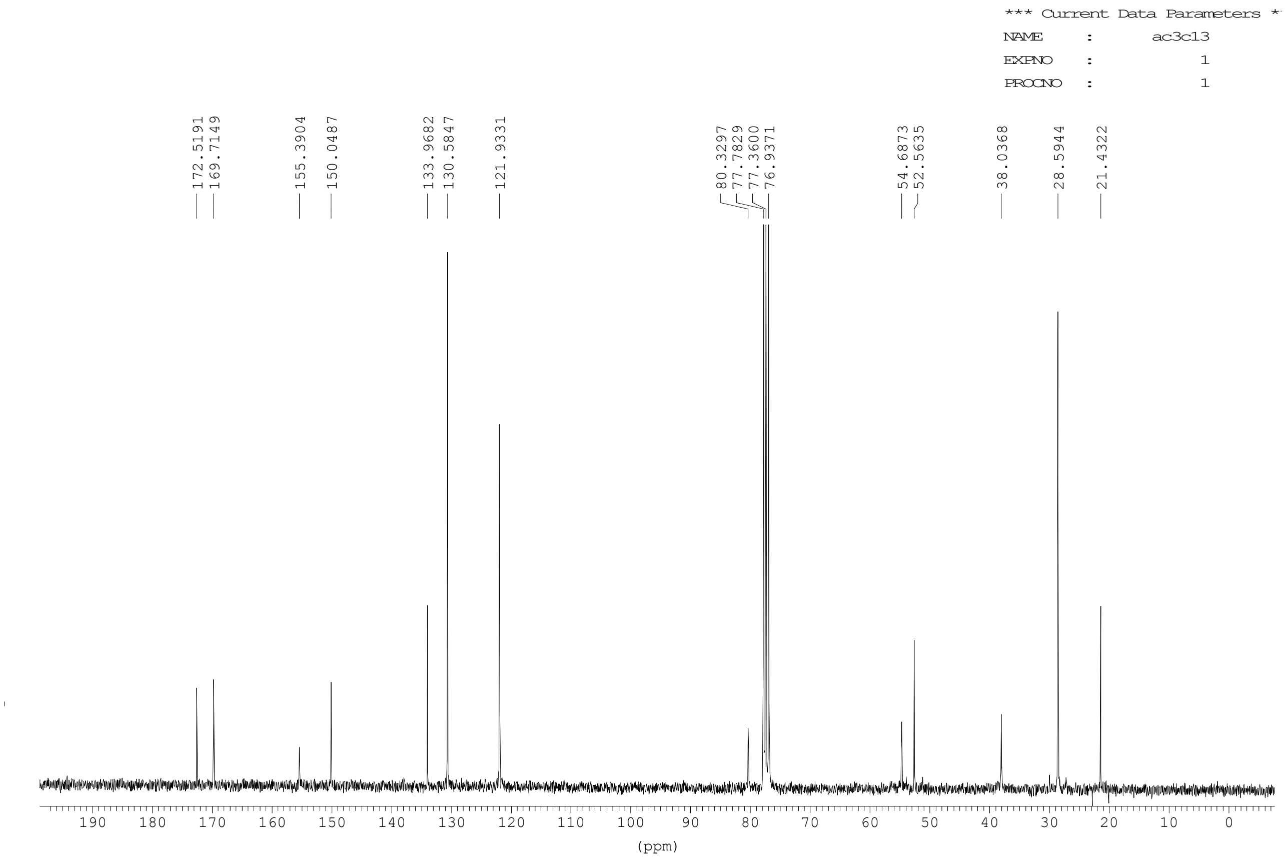
*** Current Data Parameters *
NAME : ac3c13
EXPNO : 1
PROCNO : 1
172.5191
169.7149
155.3904
150.0487
133.9682
130.5847
121.9331
80.3297
77.7829
77.3600
76.9371
54.6873
52.5635
38.0368
28.5944
21.4322
190 180 170 160 150 140 130 120 110 100 90 80 70 60 50 40 30 20 10 0
(ppm)

Sample **9a**

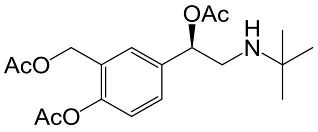
OAc
H
N
AcO
AcO

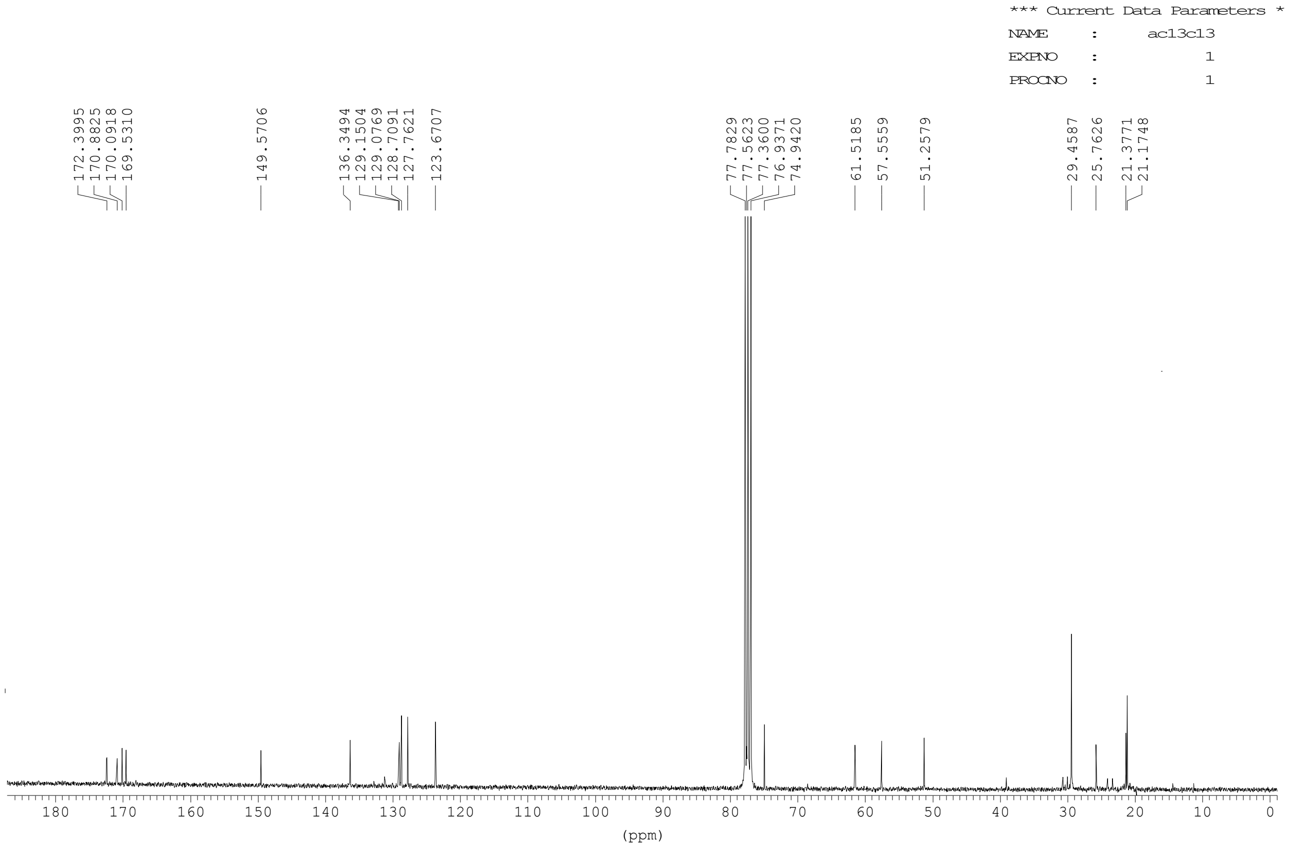
*** Current Data Parameters *
NAME : ac13c13
EXPNO : 1
PROCNO : 1
172.3995
170.8825
170.0918
169.5310
149.5706
136.3494
129.1504
129.0769
128.7091
127.7621
123.6707
77.7829
77.5623
77.3600
76.9371
74.9420
61.5185
57.5559
51.2579
29.4587
25.7626
21.3771
21.1748
180
170
160
150
140
130
120
110
100
90
80
70
60
50
40
30
20
10
0
(ppm)

Sample **10a**

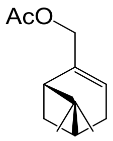
AcO

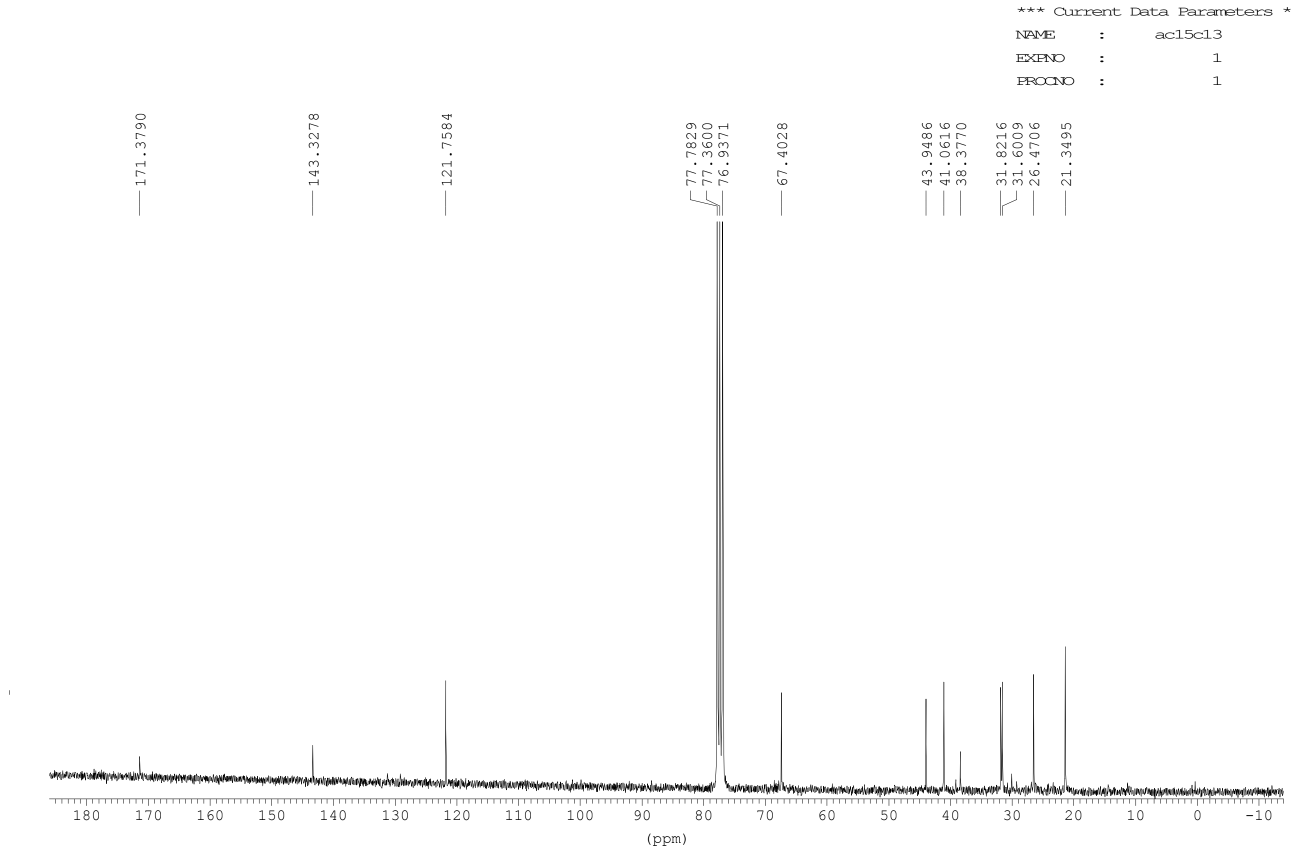
*** Current Data Parameters *
NAME : ac15c13
EXPNO : 1
PROCNO : 1
171.3790
143.3278
121.7584
77.7829
77.3600
76.9371
67.4028
43.9486
41.0616
38.3770
31.8216
31.6009
26.4706
21.3495
180
170
160
150
140
130
120
110
100
90
80
70
60
50
40
30
20
10
0
-10
(ppm)

Sample **12a**

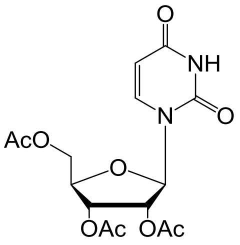
O
NH
N
O
AcO
O
OAc
OAc

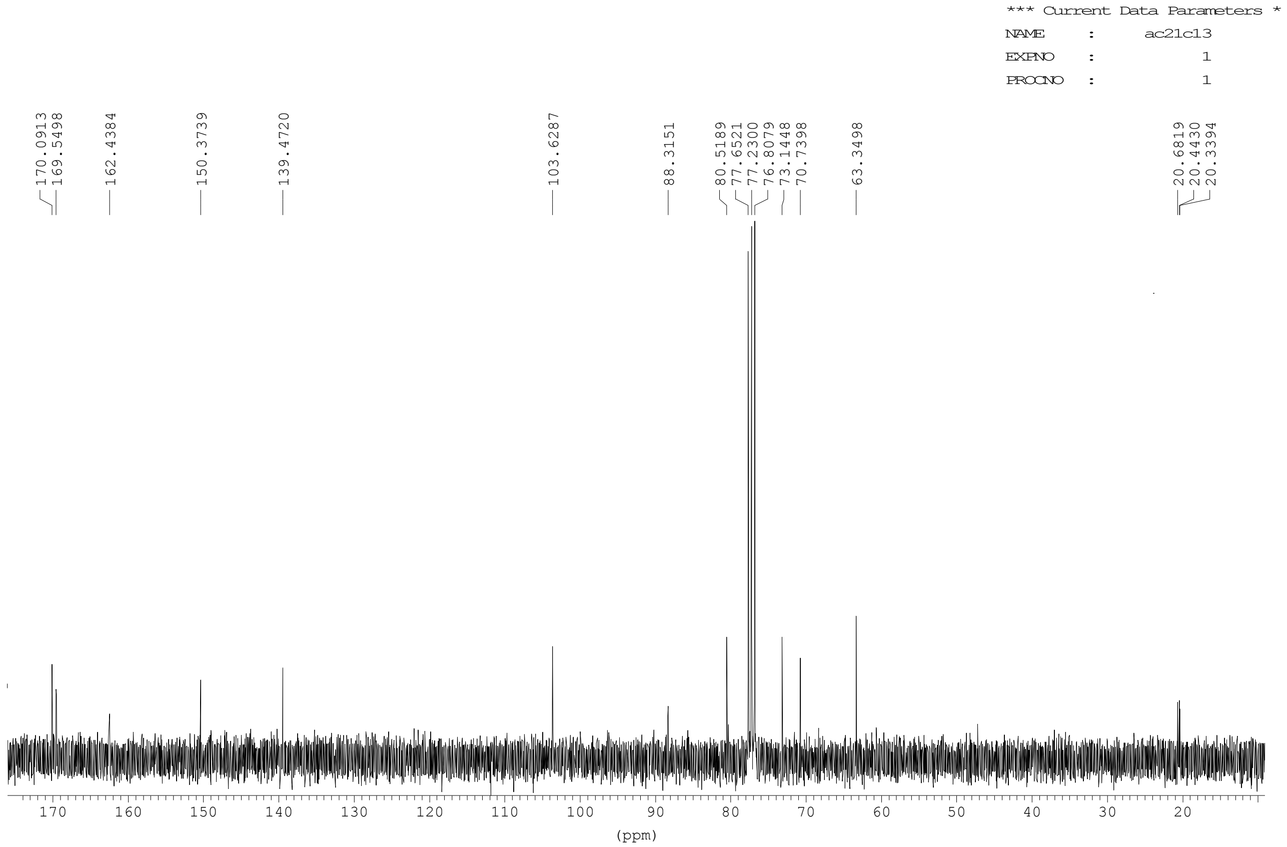
*** Current Data Parameters *
NAME : ac21c13
EXPNO : 1
PROCNO : 1
170.0913
169.5498
162.4384
150.3739
139.4720
103.6287
88.3151
80.5189
77.6521
77.2300
76.8079
73.1448
70.7398
63.3498
20.6819
20.4430
20.3394
170
160
150
140
130
120
110
100
90
80
70
60
50
40
30
20
(ppm)

Sample **13a**

(mix, characterization of the major product)

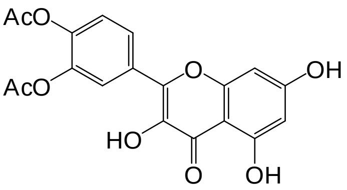

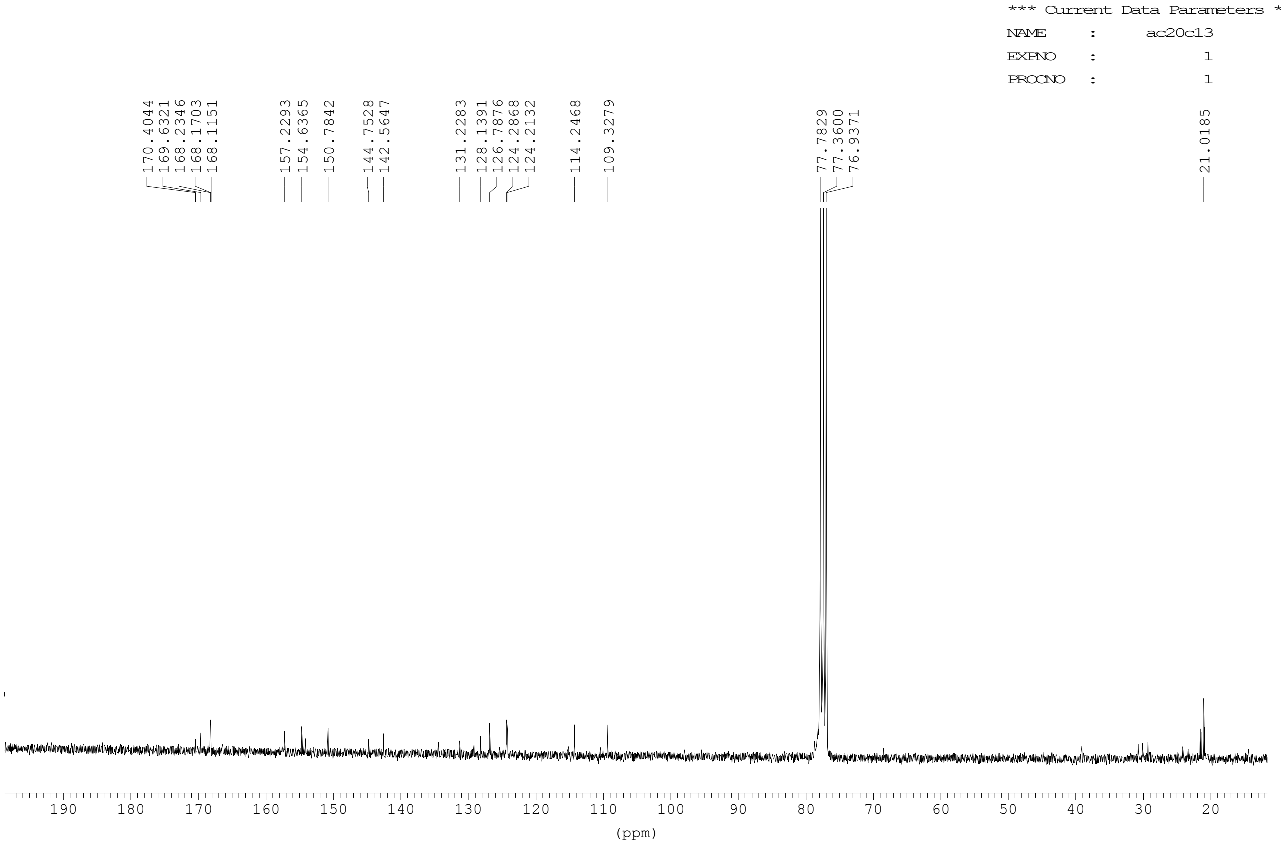

Sample **16a**

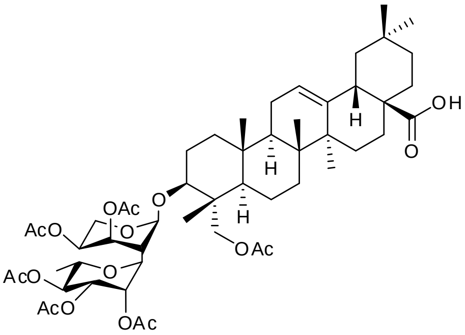
OH
H
O
H
O
OAc
AcO
O
H
OAc
AcO
O
AcO
OAc

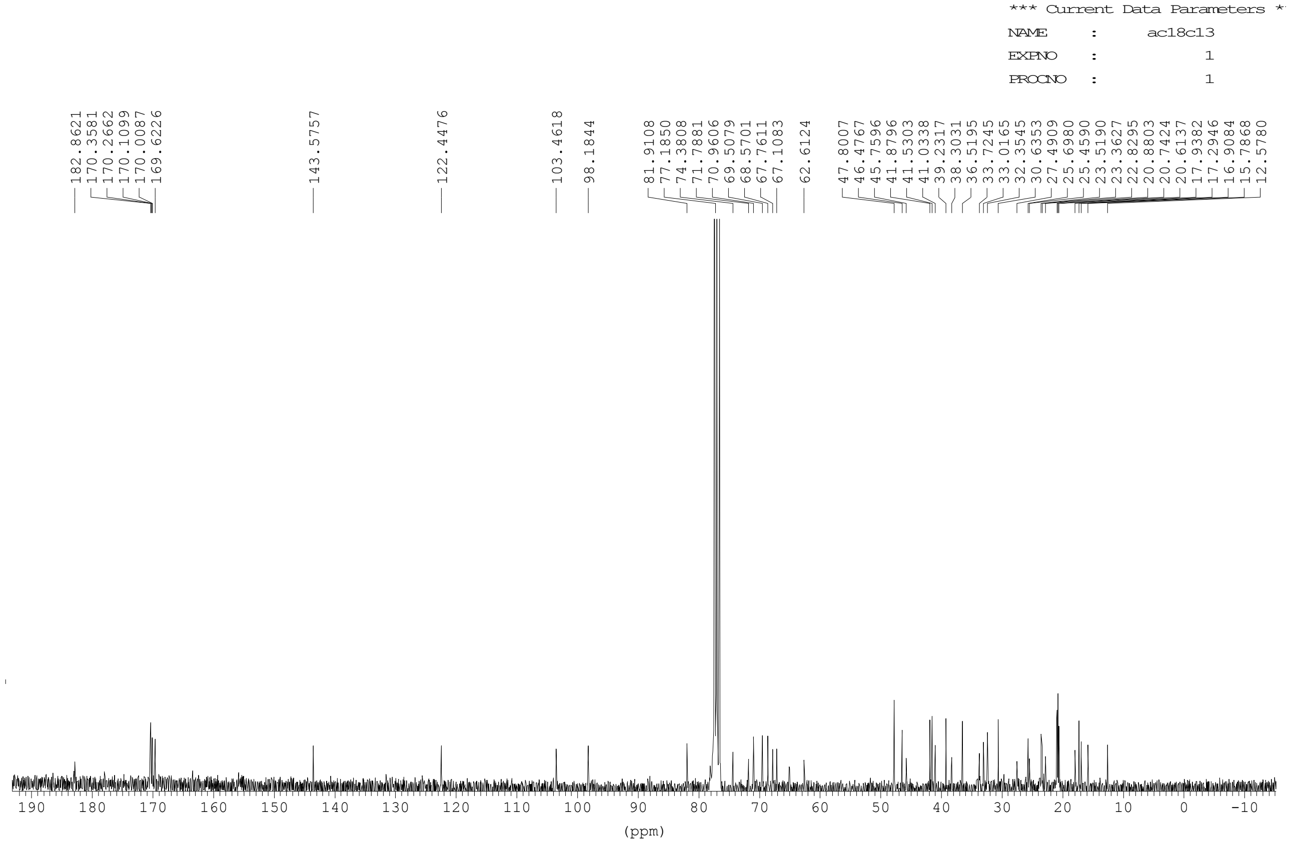
*** Current Data Parameters *
NAME : ac18c13
EXPNO : 1
PROCNO : 1
182.8621
170.3581
170.2662
170.1099
170.0087
169.6226
143.5757
122.4476
103.4618
98.1844
81.9108
77.1850
74.3808
71.7881
70.9606
69.5079
68.5701
67.7611
67.1083
62.6124
47.8007
46.4767
45.7596
41.8796
41.5303
41.0338
39.2317
38.3031
36.5195
33.7245
33.0165
32.3545
30.6353
27.4909
25.6980
25.4590
23.5190
23.3627
22.8295
20.8803
20.7424
20.6137
17.9382
17.2946
16.9084
15.7868
12.5780
190 180 170 160 150 140 130 120 110 100 90 80 70 60 50 40 30 20 10 0 -10
(ppm)

Sample **17a**

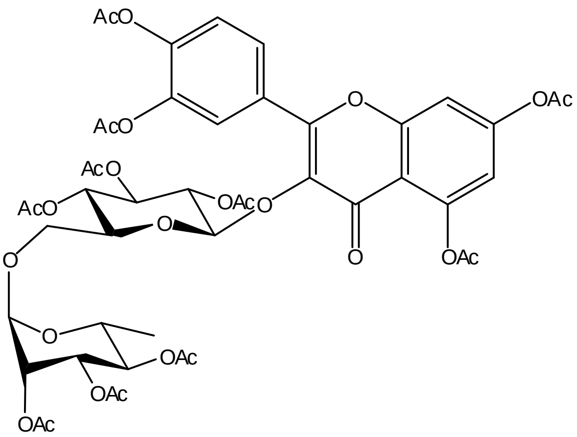

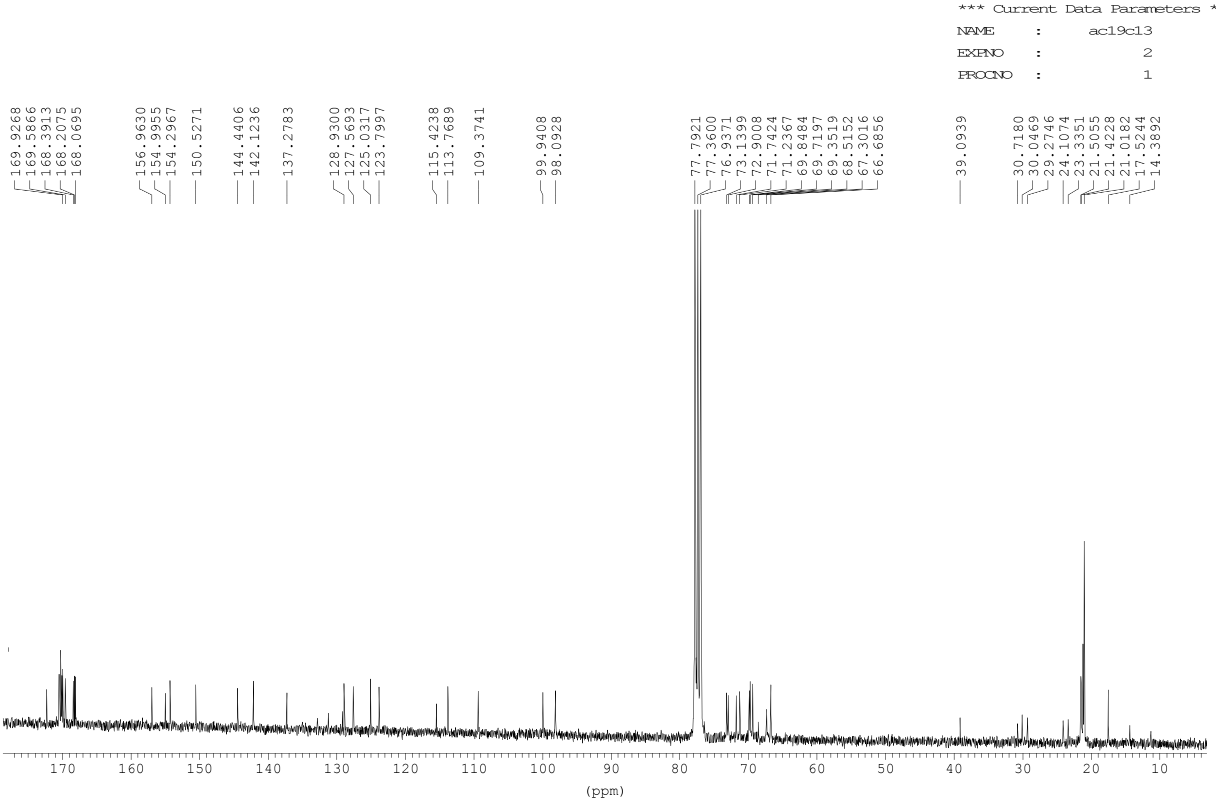

References

1. SDBS Web:http://sdbs.db.aist.go.jp (National Institute of Advanced Industrial Science and Technology, 02/03/2015)
2. Procopio, A.; Alcaro, S.; Nardi, M.; Oliverio, M.; Ortuso, F.; Sacchetta, P.; Pieragostino, D.; Sindona, G. *J. Agric. Food Chem.* **2009,** *57,* 11161–11167
3. Horton, D.; Lauterback, J. H. *J. Org. Chem.* **1969,** *34*, 86–92.
4. Shull, B. K.; Wu, Z.; Koreeda, M. *J. Carbohydr. Chem.* **1996**, *15*, 955–964.
5. Constable, D. J. C.; Curzonsb, A. D.; Cunningham, V. L. *Green Chem.* **2002,** *4,* 521–527.
